# Preserving the Biotransformation Potential of Activated Sludge in Time: Toward Reproducible Incubation Experiments for Persistence Assessment

**DOI:** 10.1021/acs.est.4c08657

**Published:** 2025-02-25

**Authors:** Martina Kalt, Chloé Iris Udressy, Yaochun Yu, Axelle Colliquet, Kathrin Fenner

**Affiliations:** † Eawag, Swiss Federal Institute of Aquatic Science and Technology, 8600 Dubendorf, Switzerland; ‡ Department of Chemistry, University of Zurich, 8057 Zurich, Switzerland; § Department of Environmental Systems Science, ETH Zurich, 8092 Zurich, Switzerland; ∥ Department of Chemistry and Applied Biosciences, ETH Zurich, 8092 Zurich, Switzerland

**Keywords:** biotransformation, persistence assessment, activated sludge, preservation, OECD 301, OECD 314

## Abstract

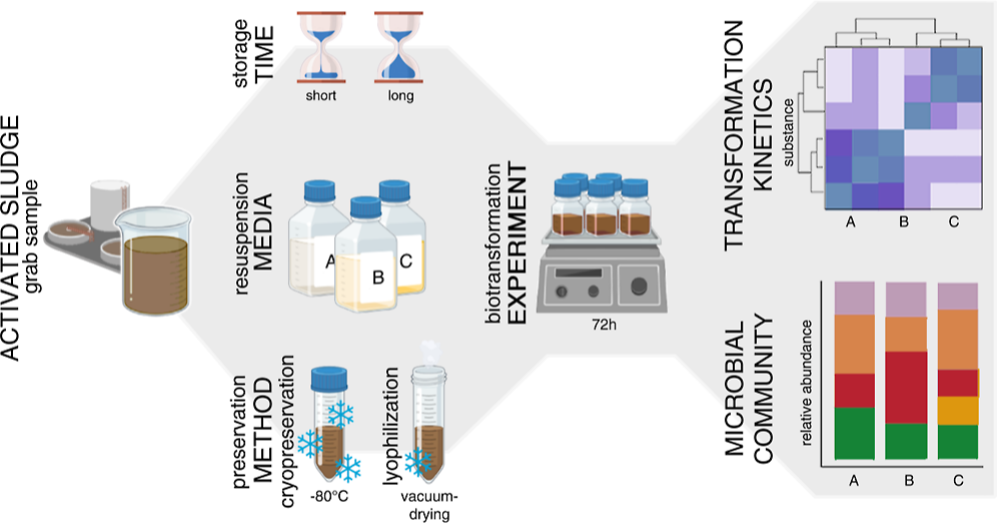

Biotransformation assays conducted in activated sludge (AS) from wastewater treatment plants (WWTPs) offer various benefits, most notably the high microbial density and comparably high bioavailability of the chemicals, enabling short experimental times of 72 h. Moreover, rate constants determined in AS experiments have shown the potential to be used as predictors for half-lives in other environmental compartments, such as soil. Therefore, biotransformation experiments with AS could serve as a valuable basis for developing standardized, high-throughput persistence tests used for screening purposes, e.g., in a benign-by-design framework, if reproducibility of experimental outcomes can be ensured. Here, we tested protocols for the preservation of AS microbial communities using lyophilization or cryopreservation. Their preservation performance was evaluated for 36 representative micropollutants (MPs) in 72 h, lab-scale batch experiments, with fresh AS as a reference. Cryopreservation, using either DMSO or glycerol as a protective agent, preserved the biotransformation potential for most of the MPs (∼65%), showing significant deviations in biotransformation kinetics almost exclusively for amine-containing substances. Lyophilization, however, performed worse with over ∼89% of MPs exhibiting significantly decreased or enhanced biotransformation compared to fresh AS. We further demonstrate nonsignificant impacts of storage time and the possibility of using artificial instead of preserved native supernatant. Major shifts in community composition based on 16S rRNA gene sequencing results aligned with biotransformation outcomes. Overall, the results suggest that our optimized cryopreservation protocol holds promise to preserve the biotransformation potential of AS and, upon further refinement and testing, might effectively support long-term reproducibility in persistence assessment.

## Abstract 2

Reproducibility, brevity, and simplicity are crucial for high-throughput persistence tests, especially for screening. Our cryopreservation method holds promise to preserve the biotransformation potential of activated sludge and may ultimately support reliable persistence assessment.

## Introduction

Due to their omnipresence, (pseudo)persistence, and potential ecotoxicological effects, micropollutants (MPs) represent a growing environmental concern.^1,2^MPs refer to synthetic organic and inorganic compounds found in the environment at concentrations ranging from ng L^–1^ to μg L^–1^, including pharmaceuticals, personal care products, pesticides, household and industrial chemicals, surfactants, and heavy metals. Source control measures (e.g., restricted use, user information, or incentive taxes) or advanced wastewater treatment^3−6^ are established approaches of mitigating MP emissions and reducing environmental exposure. An additional avenue recently promoted by the European Commission involves preventing potentially harmful environmental effects from chemical use and manufacturing by developing more sustainable chemicals with low environmental hazard potential (safe-and-sustainable-by-design (SSbD)^7^). In consequence, environmental hazard assessments of MPs should therefore be implemented not only for already marketed substances but also at the earliest stages of chemical design.

Environmental persistence of MPs is considered a key hazard and is typically evaluated for environmental compartments such as water, soil, and sediments.^8^ However, given the fact that wastewater treatment plants (WWTPs) constitute a primary release pathway into aquatic systems,^9^ the degradation of MPs during wastewater treatment significantly influences environmental exposure, particularly for down-the-drain chemicals. During activated sludge (AS) treatment, organic compounds and nutrients are aerobically degraded by microorganisms in aerated tanks. AS communities have a large biological diversity and contain a variety of viruses, bacteria, archaea, fungi, algae, protozoa, and metazoans.^10,11^ From an experimental perspective, biotransformation experiments with AS sourced from WWTPs offer several advantages: (i) AS is easy to handle and readily available; (ii) the solids-to-water ratio is lower than in soil and sediment systems, yet microbial biomass density is high, leading to relatively short experimental durations needed to observe biotransformation, ranging from 24 to 72 h; and (iii) biotransformation rates determined in AS experiments have been shown to be good predictors of half-lives in soil if corrected for differences in bioavailability.^12^ Taken together, these aspects suggest that degradation experiments with AS could potentially provide a useful basis for designing more high-throughput persistence tests for screening purposes, e.g., in a benign-by-design context.^13^

Under the Registration, Evaluation, Authorisation and Restriction of Chemicals (REACH) regulation,^14^ two testing guidelines, i.e., Organization for Economic Cooperation and Development (OECD) 301^15^ and OECD 314,^16^ use AS to evaluate the biodegradability of chemicals. Despite standardized procedures, the reliability of the outcomes of such tests with AS is compromised by their limited reproducibility caused by inocula diversity.^17^ Changes in the composition and functioning of AS microbial communities have indeed been reported on geographical and temporal scales due to variations in wastewater composition and operational conditions.^18,19^ For instance, the solids retention times (SRTs) directly modulate the diversity of a microbial community by determining how well slow-growing microorganisms such as nitrifying bacteria can establish.^20^ Under standard storage conditions (4 °C), the stability and representativeness of AS can, however, not be maintained for more than a few days at maximum. Yet, to the best of our knowledge, alternative preservation strategies for AS have not been reported in the literature to date.

In a chemical hazard assessment context, the long-term preservation of AS microbial communities would be an important methodological contribution to ensure the reproducibility of biotransformation testing and ultimately to provide more reliable conclusions. However, considering that the success rate of different preservation methods has been reported to be highly strain-dependent,^21^ preserving a diverse microbial community such as AS raises many challenges. Despite the increasing relevance of mixed microbial communities for biotechnological applications (e.g., bioconversion of lignocellulose to liquid fuels,^22^ fecal transplants and probiotics research,^23^ and active biomass backup for denitrification systems^24^), the development of long-term preservation protocols for various types of mixed microbial communities is an emerging field of research in comparison to long-term preservation methods for axenic cultures.^23^ For the latter, the two major long-term preservation methods include cryopreservation (cryo) (storage at subfreezing temperatures, e.g., at −80 °C or in liquid nitrogen) and lyophilization (lyo) (vacuum desiccation of frozen cell suspensions and storage at temperatures above 0 °C).^21,25^ In order to achieve higher survival rates, protective agents such as dimethyl sulfoxide (DMSO), glycerol (gly), peptone, methanol (MeOH), or sorbitol are typically added to cells prior to freezing for both cryo and lyo.^26^ Although lyo requires specialized equipment and follows a more detailed protocol, it offers significant advantages in terms of simplicity in transportation and storage (in the dark at 5–15 °C) compared to cryo.^21^

This study aimed to develop and evaluate the performance of long-term preservation and resuscitation methods for AS microbial communities. To this end, a literature review of mixed microbial community preservation methods was performed to determine preservation and resuscitation parameters presumably suited for the long-term storage of AS microbiomes. The effectiveness of selected methods in maintaining AS communities and the biotransformation potential was then assessed through laboratory-scale batch incubation assays with 36 representative test substances, including artificial sweeteners, pesticides, and pharmaceuticals, in freshly sampled and preserved AS. Biotransformation rate constants and community composition based on 16S rRNA gene amplicon sequencing were compared across treatments and test substances to identify preservation strategies that are suited to maintain the MP biotransformation potential of AS over extended periods of time.

## Materials and Methods

### Preservation Method

In order to evaluate the effect of preservation procedures and storage duration on the biotransformation potential of AS, three sets of biotransformation experiments (freshly collected AS and preserved AS after short- and long-term storage) of 72 h each were performed. A fraction of native AS, sampled from a nearby WWTP from the aerated nitrifying treatment basin, was directly used in the first incubation experiment (fresh) to assess the reference biotransformation potential of freshAS (details in Supporting Information (SI) Section S2.2.1). The other portion of native AS was treated for preservation in 50 mL portions using the various methods described below. After storage durations of 1 week (short) and 17 weeks (long), preserved AS was reactivated and prepared for the respective biotransformation experiment.

For the preservation procedure, native AS was centrifuged (4000 rcf, 5 min), and the activated sludge supernatant (SN) was kept for later use as a resuspension medium. AS pellets centrifuged from 50 mL portions were then resuspended with protecting agents before preservation. For the cryopreservation method (cryo), two different cryoprotective solutions were prepared by diluting each cryoprotective agent (CPA), i.e., DMSO (Sigma-Aldrich) and glycerol (Fisher Bioreagents), to final concentrations of 5% (v/v) and 10% (v/v), respectively, in autoclaved groundwater (121 °C, 2 bar, 20 min). Groundwater was preferred over nanopure water to avoid inducing osmotic stress on microorganisms during the preservation treatment. AS pellets were mixed with 5 mL of cryoprotective solution per gram of wet biomass pellet. Whereas the biomass was left to equilibrate with the glycerol solution for 60 min to allow for cellular uptake, DMSO-treated samples were frozen immediately after the addition of the cryoprotective solution (approximately 15 min) to avoid the potential cytotoxic effects of DMSO. Biomass resuspended in the cryoprotective solution was mixed thoroughly by vigorous manual shaking before being transferred to a–80 °C freezer. The lyoprotective solution was prepared by dissolving skimmed milk powder (Sigma-Aldrich) and trehalose dihydrate (Sigma-Aldrich) in autoclaved groundwater (121 °C, 2 bar, 20 min) at final concentrations of 12% (w/w) and 7% (w/w), respectively. AS pellets were mixed with 2.5 mL of lyoprotective solution per gram of wet biomass pellet. Mixtures were homogeneously mixed by vigorous manual shaking before being placed in a–80 °C freezer for about 3 h. Upon complete freezing, the frozen mixtures were rapidly transferred to a GT2 Basic SRK System Technik lyophilizer (SRK-Systemtechnik GmbH, Riedstadt, Germany). After desiccation for 5 d, freeze-dried samples were stored at 4 °C in the dark.

After storage durations of 1 week (short) or 17 weeks (long), preserved AS samples were thawed at room temperature (RT), followed by three washing steps and subsequent resuspension in the corresponding resuspension media [SN, artificial wastewater (AW), or artificial effluent (AE)]. The suspensions were equilibrated in the batch reactors for 16 h (overnight) at RT on an orbital laboratory shaker operating at 150 rpm before the start of the biotransformation experiments. We used preserved SN (filtered and frozen at −20 °C) as one resuspension medium since it was expected to most closely mimic biotransformation experiments conducted with native AS. However, due to the significant efforts required for SN storage, we also explored synthetic wastewater supernatant as a highly reproducible alternative resuspension medium. As AW is a well-established and widely used artificial medium for laboratory wastewater experiments,^27−29^ it was tested as a possible replacement for resuspension media in the first two sets of experiments (fresh and short experiments). However, since AW was significantly higher in carbon and phosphorus contents compared to SN and therefore most likely too nutrient-rich, we additionally developed AE with a composition more similar to SN and used it as an artificial resuspension medium in the third (long) experimental set (details in SI Tables S4 and S3). The complete resuscitation procedure is described in detail in SI Section S2.2.

### Experimental Setup of Sludge Reactors

Following the initial biotransformation experiment conducted with fresh, untreated AS in SN, two subsequent experiments were performed to test the effects of short and long-term storage on preserved AS. Each of these experiments involved samples and two cryo samples preserved with either DMSO or gly as the CPA. Each preserved sample was resuscitated by using either native SN or artificial media (AW or AE). In total, 12 different treatment conditions were tested and compared to the biotransformation potential of freshAS. The experimental setup of the sludge reactors was adapted from Trostel et al.^30^ Briefly, 100 mL aerated bottle reactors (Schott bottles) containing 50 mL of the different AS suspensions (each treatment conducted in triplicate) were spiked simultaneously with a test substance mixture at an initial concentration of 10 nM each (details provided in SI Section S2.1). The 36 test substances were selected based on their well-documented transformation behavior in AS, their expected detectability using HPLC-HRMS/MS, and their structural diversity and range of functional groups. To verify our previous knowledge of the sorption behavior of our test substances, a sorption control (SC) experiment was conducted along with the first biotransformation assay. For SC, AS was autoclaved to deactivate the biomass to observe potential sorption onto organic matter (for details and results, see SI Section S2.5).

Samples were collected from the biotransformation reactors at −1 h (before spiking), 0 h (immediately after spiking), and 1, 2, 4, 8, 24, 48, and 72 h. These samples were centrifuged, and the supernatant was transferred to LC–MS amber vials, spiked with an internal standard solution (final concentration of 2 μg L^–1^), and stored at −20 °C until analysis. Calibration curves were prepared in Evian water with concentrations of MPs ranging from 0.01 to 50 nM. A more detailed description of the experimental setup can be found in SI Section S2. The experiments were monitored by taking daily pH measurements of each reactor and by measuring ambient temperature at 5 min intervals. Samples for volatile suspended solids (VSS), total suspended solids (TSS), flow cytometry, and 16S rRNA gene amplicon sequencing were taken before (−1 h) and after (72 h) the experiment for each treatment. For details on methods and results, see SI Section S3.

### Micropollutant Analysis

To determine the chemical concentration and biotransformation rate constant for each test substance, the procedure described by Desiante et al.^31^ was adapted (detailed in SI Section S2.4). Briefly, reversed-phase liquid chromatography coupled to a QExactive Plus mass spectrometer (Thermo Fisher Scientific) was used to obtain positive and negative full-scan MS spectra. This was followed by data-dependent MS2 analysis (top 3 for positive and top 1 for negative scans) with the [M + H]^+^ and [M–H]^−^ masses included in the inclusion list. Target quantification of test substances was performed by using TraceFinder 5.1 software (Thermo Fisher Scientific). Further data evaluation was conducted using R version 4.1.1. Concentration–time series averaged over the experimental triplicates were used to calculate first-order rate constants, assuming pseudo-first-order kinetics (refer to SI Section S4). For the applicability of the kinetic model on the linearized data, at least three points in time had to be quantifiable, defining an indicative maximal quantifiable rate constant *k*_max_ as 90% biotransformation within 2 h (1.151 h^–1^). The corresponding minimal rate constant that could reliably be quantified given experimental, instrumental, and measurement evaluation uncertainty was set at *k*_lim_ = 0.0015 h^–1^, corresponding to less than 10% dissipation over the experimental time of 72 h. Additional quality tests for the estimated rate constants are described in SI Section S5. To compare biotransformation trends across the fresh treatment and preservation methods, heatmaps were created using the R package pheatmap.^32^

### Microbial Community Analysis

For 16S rRNA amplicon sequencing, DNA was extracted from the pellet of 1 mL sludge samples using the DNeasy PowerBiofilm kit (QIAGEN). For gene sequencing, the 16S rRNA gene was amplified with universal primer sets 314F/806R targeting the V3–V4 regions. Purified and pooled polymerase chain reaction (PCR) amplicons were sequenced on an Illumina NovaSeq platform (250 bp paired-end reads) at Novogene. For details on the bioinformatics workflow, see SI Section S3.5.

## Results and Discussion

### Quality Assurance

The three sets of biotransformation experiments (fresh, short, and long) are considered reliable and comparable based on the low variability of all assessed variables (pH, VSS, TSS, viable cell count, and test substance concentrations; see SI Section S3) across triplicates. The generated concentration–time series underwent a quality check, after which five MPs were excluded from further evaluation, namely, atrazine, climbazole, dextromethorphan, emtricitabine, and levetiracetam (see SI Section S5). The treatment-specific biotransformation rate constants for the remaining 31 substances are summarized in an overview heatmap in SI Section S9. Four MPs, namely, acesulfame, atorvastatin, pravastatin, and propachlor, were excluded from further analysis as the rate constants exceeded *k*_max_ in most of the samples. For the remaining 27 test substances, which met the quality criteria and fell within the range of *k*_min_ (0.001 h^–1^) to *k*_max_ (1.151 h^–1^), the rate constants ranged from 0.002 to 1.119 h^–1^, corresponding to half-lives between 37 min and 13.9 d. Individual rate constants that could not be determined and/or did not fulfill quality criteria were replaced with *NA*.

### Overall Recovery of Biotransformation Potential upon Preservation

**Figure 1:**
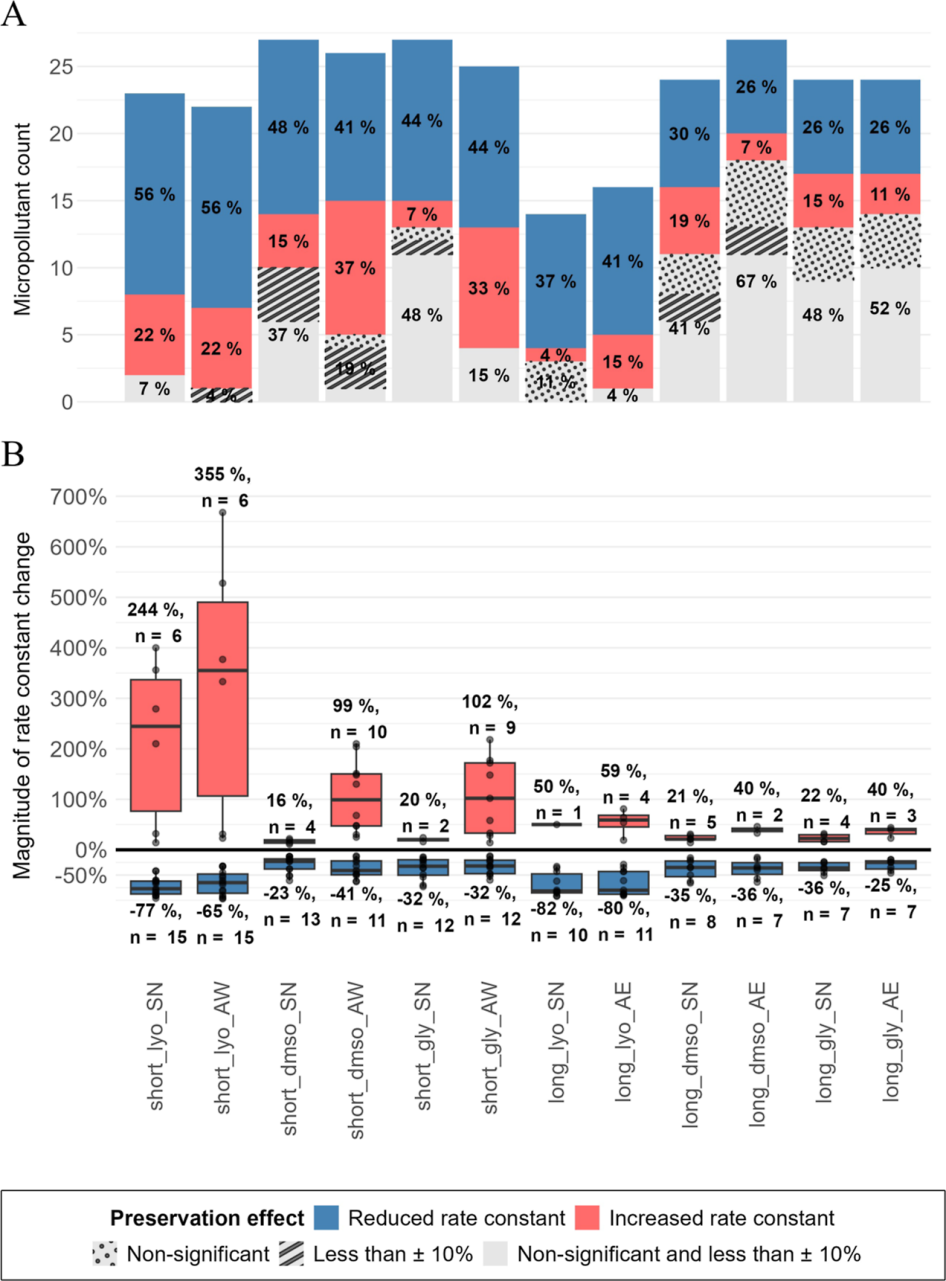
(A) Overview of preservation effects: number and percentage of rate constants (expressed relative to the total of 27 test substances) that were changed significantly (*p*-value <0.05) and by more than 10% (reduced (blue) and increased (red)), as well as rate constants that were not significantly changed (white and circles) or by less than 10% (stripes) compared to the rate constants from the fresh AS experiment. Order is according to preservation treatment. Rate constants that do not fulfill quality criteria and hence were not quantifiable (*NA*) are not displayed, leading to different total counts of MPs with quantified rate constants for each treatment. Note that many of the *NA* cases were due to nonsignificant degradation (<*k*_min_), suggesting that the number of significantly reduced rate constants, e.g., for long_lyo, is actually higher than suggested by the quantifiable rate constants displayed. (B) Magnitude of relative deviations of rate constants in each preservation treatment compared to the fresh AS experiment for test substances with rate constants that differ significantly (*p*-value <0.05) and by more than 10% from those in the control experiment. n indicates the numbers of MPs accounted per treatment.

In total, 12 different preservation treatments were tested, representing different combinations of the three resuspension media (SN, AW, and AE), preservation methods (lyo with trehalose and skimmed milk, cryo with DMSO, and cryo with gly), and preservation durations (short and long). The preservation methods significantly altered the biotransformation kinetics of 33% (long_dmso_AE) to 78% (short_lyo_AW) of the study compounds (Figure 1A). Within each treatment, the majority of significantly affected micropollutants exhibited slower biotransformation after preservation. On average across all treatments, a fold change of roughly a factor of 2 was observed for both compounds exhibiting significantly increased and decreased relative rate constants, i.e., average increases and decreases of +85 ± 95% and −47 ± 17%, respectively (Figure 1B). However, extreme values for individual cases (i.e., specific MPs and treatments) are observed for only increased relative rate constants. The number of rate constants that deviated by less than ±10% and/or showed nonsignificant changes (*p*-value >0.05) compared to biotransformation experiments with fresh AS was clearly higher for cryo compared to lyo. Within cryo, the use of gly as CPA appeared to offer a slight advantage in terms of the number of nonsignificantly altered rate constants. However, the influence of the resuspension medium used (SN, AW, or AE) on these numbers was more decisive and mostly masked minor differences due to different CPAs or storage times (Figure 1B).

### Differential Effects of Preservation Methods on MP Biotransformation Potential

Among the two preservation methods tested, lyo induced the most substantial changes in MP biotransformation potential in both the number of significantly affected substances and the magnitude of change of those significantly altered rate constants (Figure 1B). For experiments conducted with SN, for instance, the percentage of MPs whose biotransformation kinetics were not significantly affected and/or by less than 10% amounted to only 11% of study compounds for the lyo method, whereas 40% to 52% of substances showed a recovered biotransformation potential similar to fresh AS in the cryo samples (SI Figure S10). Moreover, the absolute magnitude of changes in biotransformation potential ranged from −73% to +218% in cryo samples but was as large as −97% to +668% in lyo samples (Figure 1B). It is further worth noting that for many compounds, no rate constants could be determined in the long_lyo treatments at all, mostly due to the fact that they were not significantly different from 0, suggesting that the proportion of reduced rate constants was even higher in long_lyo than indicated by the quantifiable rate constants given in Figure 1A. These findings align with the visual observation that lyo samples were severely deteriorated after the long storage period.

The most extreme increases in biotransformation potential (210–668%) were all observed for the same four sulfonamides (sulfamethazine, sulfamethoxazole, sulfadiazine, and sulfapyridine) in lyo samples (SI Figure S10). In contrast, all other test substances (except for atenolol) showed either comparable or significantly slower biotransformation in lyo samples compared to fresh AS. In AS, co-metabolic biotransformation of sulfonamide antibiotics has been shown to proceed via a pterin-conjugation pathway as a result of their inhibiting action on folic acid synthesis.^33^ Since folic acid synthesis is fundamental to cellular production and maintenance for a broad diversity of microorganisms, the biotransformation rate constants of these four sulfonamides have been suggested to be intrinsically dependent on microbial growth.^20^ Indeed, we have previously shown that biomass-normalized biotransformation rate constants for the same four sulfonamides decrease along an increasing SRT gradient, which aligns with slower biomass growth rates at higher SRTs.^20^ Here, we observed substantially larger rates of increase in viable cell concentrations in our lyosamples than in fresh and cryo samples (SI Section S3.4), which might indeed explain the observed increases in biotransformation rate constants of those same four sulfonamides in lyoAS suspensions. We do note, however, that while growth was similar for short and long storage periods (SI Section S3.4), sulfonamide biotransformation rates increased less for long_lyo_SN than for short_lyo_SN samples. This suggests that additional phenomena, such as differences in microbial community shifts discussed further below, could also contribute to the changes in the biotransformation behavior. The fifth sulfonamide antibiotic, sulfathiazole, was not significantly faster biotransformed in lyo samples and hence responded differently to experimental changes than the other four sulfonamides. This singular behavior of sulfathiazole has also been noted by Achermann et al.^33^ and Meynet et al.,^34^ who suggested a differing biotransformation route including an oxidation of the thiazole group, which has been previously reported to be an active site of biotransformation.^35^

The faster biotransformation of the four sulfonamides in the lyo samples, which hence can presumably be attributed to the observed substantial microbial growth, provides important insights into the effects of lyoas a preservation method. The observed growth, most likely related to low nutrient limitations in combination with the initially low concentrations of viable cells after lyoand resuscitation (SI Section S3.4), points toward severe cell rupturing and thus a considerably negative impact of the lyo method on a large number of microorganisms in AS. This interpretation of the sulfonamide and biomass data is then also consistent with the significantly lower biotransformation rate constants of the vast majority of the remaining MPs in lyo samples relative to those in fresh AS. As a consequence of the substantial freezing and vacuum-drying stresses of lyo, a prolonged lag phase after the rehydration of lyo samples could furthermore be expected according to literature.^21^ As a matter of fact, lag phases in concentration–time series were observed for valsartan in short_lyo_AW samples and for metoprolol in short_lyo_SN and short_lyo_AW samples, although these lag phases were less significant for metoprolol (see SI Section S4).

### Clusters of Biotransformation Responses Highlight Effects of Cryopreservation

**Figure 2:**
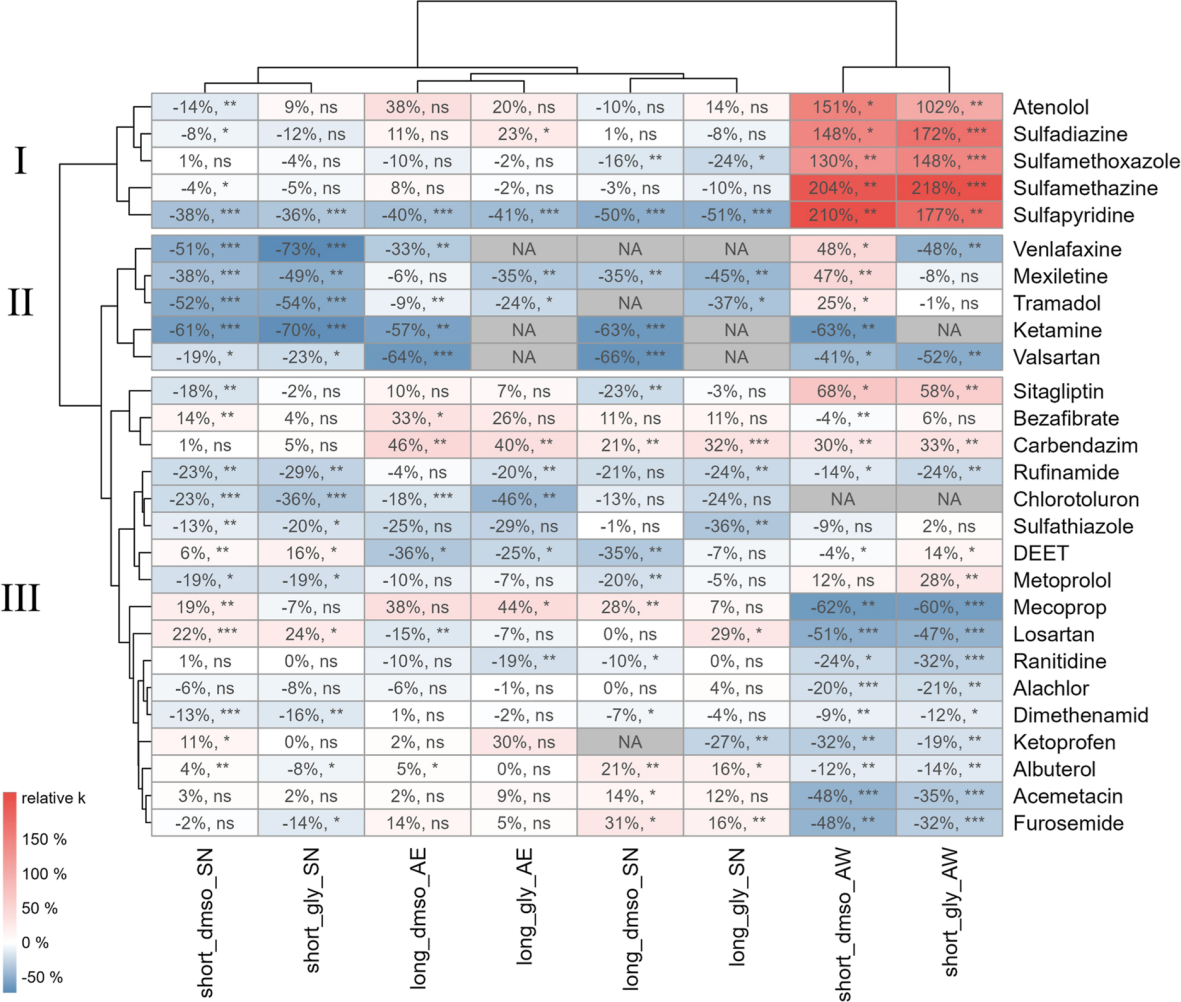
Relative deviation of rate constants in cryopreservation treatments compared to the fresh AS control experiment, including indication of statistical significance of differences: *p* > 0.05 (ns, not significant), *p* ≤ 0.05 (*), *p* ≤ 0.01 (**), *p* ≤ 0.001 (***), and *NA*: not applicable, i.e., rate constant could not be quantified. Clustering according to relative deviations highlights three distinct groups of MPs (e.g., I, II, and III), each exhibiting a unique response of biotransformation rate constants to preservation methods, preservation time, and resuspension media used.

To further investigate the effects of cryo on different test substances without the growth dependency of sulfonamides biotransformation acting as a bias, a clustering analysis was performed with cryo samples only (Figure 2). The hierarchical clustering according to relative deviations of biotransformation rate constants compared to those of fresh AS revealed three distinct responses of MP biotransformation as a function of CPA, storage duration, and resuspension media used. Table 1 summarizes the effects of CPA, storage duration, and resuspension media on the biotransformation potential for each of the three clusters.

**Table 1:** Overall Fingerprints of Micropollutant Clusters per Medium and Preservation Time^a^

cluster	short_SN	long_AE	long_SN	short_AW
I	ns	ns	–/ns	++
II	– –	–/NA	–/NA	–/+
III	ns	ns	ns	– –

a ++: ≥50%, +: 10–50%, ns: <±10% or nonsignificant, −: −10 to −50%,– – : ≤−50%, and *NA*: not applicable. Percentages refer to the relative deviations of rate constants derived in the fresh AS experiment.

Substances in cluster II showed the most notable decreases in biotransformation rate constants, independent of the applied CPA or resuspension medium (Table 1 and Figure 2, cluster II). Within this cluster, three out of five substances (mexiletine, tramadol, and venlafaxine) are compounds for which Gulde et al.^36,37^ reported bioaccumulation by protozoa was reported to be a relevant fate process in AS. For these and other aliphatic amine-containing compounds featuring an acid dissociation constant (p*K*_a_) in the range of 7–10 and log*P* values between 0.4 and 4.2, the authors describe an “ion-trapping mechanism”, which revolves around the differential speciation of these substances at environmentally relevant pH values. Predominantly neutral in the extracellular phase of AS (pH ≈ 8), these compounds readily diffuse through prokaryotic and eukaryotic cell membranes. In eukaryotic microorganisms such as protozoa, they potentially further reach acidic vesicles (pH < 5), where they get trapped following protonation, which impedes their diffusion back to the cytosol (pH ≈ 7). The presence of the ion-trapping mechanism has been shown to lead to deviation from first-order kinetics, with aqueous concentrations gradually decreasing at first and stabilizing upon equilibration of the bioaccumulation process.

In the present study, this expected pattern was apparent in the concentration–time series of tramadol, venlafaxine, and ketamine in fresh AS (SI Section S4). For the former two, protozoic bioaccumulation in native AS had already been reported in Gulde et al.^36^ Ketamine is another aliphatic amine-containing compound, with a p*K*_a_ of 7.3 and an experimental log*P* value of 2.18.^38^ The similarity of the concentration–time series of ketamine to those of tramadol and venlafaxine (SI Section S4) supports the hypothesis that the ion-trapping mechanism is also relevant for this substance. Less clear deviation from first-order kinetics was observed for mexiletine, most likely as a result of the predominance of bacterial biotransformation over protozoic bioaccumulation (SI Section S4). Similarly, ranitidine, metoprolol, albuterol, and atenolol, another four aliphatic amines, did not even fall into cluster II. Again, this deviating behavior may presumably be attributed to a larger contribution of bacterial biotransformation relative to protozoic bioaccumulation in the overall fate of this substance. This is supported by the absolute rate constants reported in SI Table S14 and Figure S9, which confirm that these four compounds are biotransformed faster than the amines grouped into cluster II (except for mexiletine, which seems to be a borderline case in terms of clustering).

Overall, the significantly lower biotransformation rate constants after cryo for the four aliphatic amine-containing test substances in cluster II together with the disappearance of deviation from first-order kinetics in cryo provide strong evidence for the loss of the ion-trapping mechanism upon cryopreservation of AS. We assume that this is due to the known low resistance of protozoa—which account for around 5–15% of AS biomass^39^—to freezing and thawing procedures as well as to shear stresses.^36^ As a consequence, rate constants measured after cryopreservation for slowly biotransformed aliphatic amines represent the extent of their actual microbial biotransformation rather than loss due to a combination of bioaccumulation and biotransformation.^37^

### Impact of Resuspension Media on Biotransformation Potential

In contrast to cluster II, the biotransformation potential of the test substances in clusters I and III was generally not significantly affected by the cryo method using both SN and AE resuspension media (nonsignificantly altered biotransformation kinetics for 54 ± 6% and 69 ± 5%, respectively, of the test substances, SI Figure S11). Similarly to substances in clusters I and III, atorvastatin, acesulfame, pravastatin, and propachlor did not show any signs of significantly faster or slower depletion in cryo treatments compared to fresh AS, based on visual inspection of their concentration–time series (SI Section S4 and Figure S9). However, these four substances were excluded from the heatmap shown in Figure 2 due to their generally rapid depletion, which impeded proper quantification of rate constants.

In contrast, when the AW medium was used for resuspension of the cryo samples, the biotransformation rate constants of at least half of the MPs in cluster III were significantly decreased, while the rate constants of all substances in cluster I were significantly increased, suggesting a relevant change in the microbial communities’ biotransformation potential upon resuspension with AW but not with SN or AE. AW is one to two orders of magnitude more concentrated in carbon, nitrogen, and phosphorus compared to SN and AE (SI Section S1.3). It is therefore likely to promote bacterial growth more effectively than SN and AE and to lead to shifts in the microbial communities by specifically promoting fast-growing microorganisms, as will be outlined later. Indeed, we again observed increases in biotransformation rate constants for the sulfonamides, which make up four out of five substances in the first cluster and have already shown growth-dependent increases in biotransformation following the lyo procedure, as detailed earlier. Yet, based on the cell counts from flow cytometry, no clear differences in growth during incubation as a function of the resuspension medium could be confirmed. In contrast to the effects seen for cluster I, observations for cluster III suggest that using AW as a resuspension medium disfavors the biotransformation of several MPs, most notably mecoprop, losartan, ranitidine, alachlor, acemetacine, and furosemide (Figure 2). We currently do not have further insights into the mechanisms underlying these observations, except for speculating that shifts in the microbial community composition and/or diversity have led to the observed declines in biotransformation potential for these substances. In summary, the results for MPs in clusters I and III suggest that using nutrient-rich AW as a resuspension medium significantly affects the observed biotransformation rate constants.

In contrast, the other variations of treatment conditions, such as replacing SN with AE as the resuspension medium, using DMSO or gly as CPAs, and varying storage durations, had minimal effects on the observed biotransformation rate constants following cryo. For the 22 test substances from clusters I and III, approximately 60% of the biotransformation rate constants were not significantly different after preservation for all remaining treatment conditions (except for cryo in combination with AW, see SI Figure S11), and those significantly changed deviated by +27 ± 10% (increased rate constant) and −23 ± 3% (reduced rate constant) on average. These results indicate that these treatments would be viable options for preserving the biotransformation potential of AS over time.

Of note, valsartan was grouped into cluster II, i.e., the cluster of substances consistently negatively affected by any preservation procedure, despite the fact that it is not a basic, amine-containing compound and would therefore not be expected to be subject to protozoic bioaccumulation. This result suggests the existence of other mechanisms leading to a strong negative effect of preservation on the biotransformation of valsartan. One possible explanation relates to the fact that valsartan biotransformation in WWTPs has been previously described to be a function of the presence of, most likely, rather specific degraders. This has been postulated based on the observation that valsartan is more strongly biotransformed by biofilms collected downstream of a WWTP compared to those upstream, likely due to colonization by specialized wastewater microorganisms contained in WWTP effluents.^31,40^ The need for specific degraders to be present in sufficient numbers for valsartan biotransformation to occur is also consistent with our findings that valsartan was one of only two of 36 study compounds that displayed a lag phase in one of the lyo samples (short_lyo_AW, SI Section S4). Our results, therefore, suggest the possibility that cryo samples might in some cases experience a loss in the biotransformation potential for substances that are catabolically degraded by specialized microorganisms. Along those lines, rufinamide, one of the substances in cluster III that also seems to be consistently negatively affected by cryo, albeit to a lesser extent than valsartan, has been previously described to show a strikingly strong dependence of biotransformation rate constants on solids retention time in WWTPs, suggesting that its degradation might also be linked to rare and slowly growing bacteria.^20^ A similar link may exist for sulfathiazole, which is proposed to undergo biotransformation involving oxidation of the thiazole ring,^33,34^ potentially explaining its distinct behavior compared to other sulfonamides.

### 16S rRNA Data Underline the Shift in Microbial Communities

AS samples collected before (point in time −1 h, unicum) and after (point in time 72 h, averaged from triplicates) the biotransformation experiment were subjected to DNA extraction and 16S rRNA gene amplicon sequencing to elucidate potential shifts in microbial community composition induced by preservation. The alpha-diversity of samples was assessed by evaluating sample richness, indicated by the number of observed species per sample, and by measuring diversity and evenness, represented by the Shannon diversity index. Consistent with results from biotransformation rate constant evaluations, where lyo samples exhibited the most significantly different biotransformation potential compared to fresh AS, a substantially lower alpha-diversity was observed in the lyo samples compared to all other samples (SI Figures S7 and 3A).

**Figure 3:**
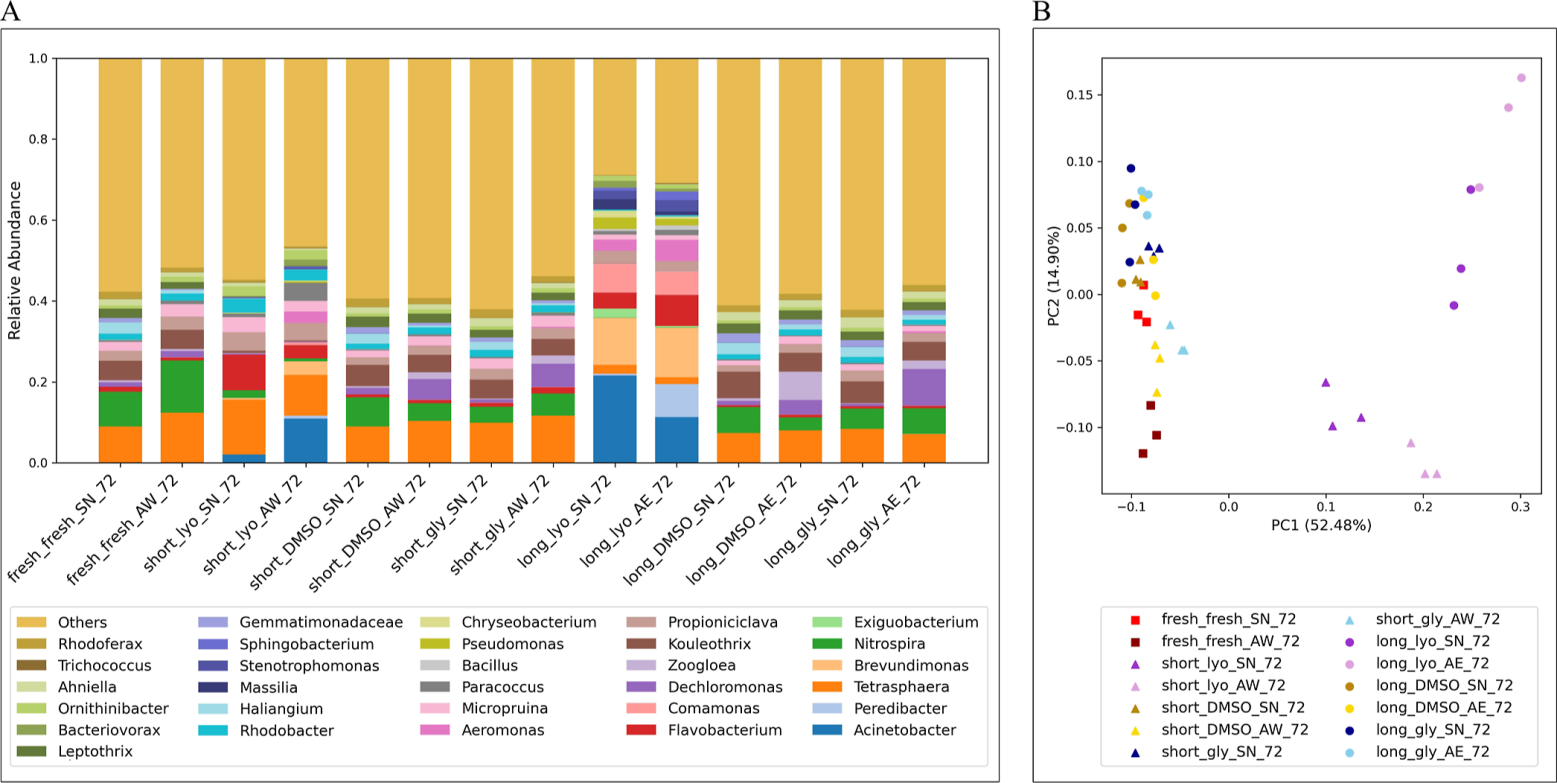
(A) Barplot of genus diversity of freshAS and of all samples at point in time 72 h. A corresponding barplot of genus diversity at point in time −1 h can be found in SI Figure S7. (B) Principal coordinate analysis (PCoA) plot of all samples after the biotransformation experiment (point in time 72 h, averaged from triplicates), color-coded by preservation method (fresh samples: red, lyo samples: purple, cryo samples with DMSO: yellow, and cryo samples with gly: blue) and symbol-coded by storage duration (square: fresh, triangle: short, and circle: long).

We further examined the changes in the composition of microbial communities in different AS samples before and at the end of the biotransformation experiments (points at −1 and 72 h). Compared to the freshly collected native AS sample, the community structures after the cryo treatments remained similar both before (SI Figure S7) and after chemical exposure (Figure 3A). In contrast, regardless of the resuspension medium used (i.e., SN, AE, and AW) and the storage duration (i.e., long and short), lyo samples exhibited significant community shifts. *Acinetobacter* was identified as the most abundant taxon in nearly all −1 h lyo samples (except for short_lyo_SN_-1) but was comparably underrepresented in fresh and cryo samples (SI Figure S7). After the biotransformation experiments (point in time 72 h, Figure 3A), besides *Acinetobacter*, *Pseudomonas* was also enriched and almost exclusively present in the samples subjected to lyo (Figure 3A). These two bacterial genera, i.e., *Acinetobacter* and *Pseudomonas*, have been identified previously as responsible for degrading sulfonamides in AS.^41−43^ Their increased presence might offer an alternative or additional explanation for growth dependence for the observed strongly increased biotransformation rate constants for the sulfonamides in lyosamples. Furthermore, the abundance of other microbial genera, including *Nitrospira* and *Tetrasphaera*, was strongly diminished at the end of the biotransformation experiment in lyo samples (Figure 3A). These reductions of certain microbial species in lyo samples compared to other treatments might explain why lyo exhibited most change in biotransformation potential compared to the fresh AS (SI Figure S10).

In the previous section, we highlighted that using nutrient-rich AW as a resuspension medium significantly affected the biotransformation rate constants of MPs, specifically those belonging to clusters I and III (SI Figure S10 and Table 1). While the use of AW visibly impacted the biotransformation potential, its effect on the microbial community structure was less pronounced. For cryo samples after the biotransformation experiments, the community composition in AW showed only slight differences compared to SN (Figure 3A), with a relatively higher abundance of genera like *Dechloromonas* and *Zoogloea*.

Given that a substantial proportion of the operational taxonomic units (OTUs) are still unclassified at the genus level (assigned as “others”), by focusing solely on assigned genera, we might overlook important information embedded in these unclassified groups. To further elucidate the differences in microbial community structures while considering the entire observed OTUs, we conducted a principal coordinate analysis (PCoA) based on the beta diversity of different samples at the end of the biotransformation experiment (point in time 72 h, Figure 3B). In line with previous observations, lyo samples were clearly separated from all other samples along the first principal component (PC1), which explained 52% of the observed variability. Additionally, the preservation time appeared to affect the microbial community in lyo samples, with longlyo samples shifted further right along the PC1 axis. No major differences were observed between cryo and fresh samples along PC1, regardless of preservation time (fresh, short, and long) or resuspension media (AW, AE, or SN) used (Figure 3B). However, the use of AW led to community shifts in the cryo and fresh samples as these samples are separated apart from the remaining samples along the second principal component axis (PC2, 15% explained variability). This result might indicate that although there are no substantial differences among AW, AE, and SN in the top abundant classified microbial genera (Figure 3A), yet-to-be-determined, most likely rare members of the microbial community might contribute to the observed differences in MP biotransformation in AW.

### Relevance and Implications for Persistence Assessment

In summary, the cryopreservation protocols tested in the present study led to nonsignificant deviations in the biotransformation kinetics for 45–73% (59 ± 9%) of 22 test substances with quantifiable kinetics, amine-containing compounds excluded. For MPs showing significant deviations, the range was from −23 ± 3% to +27 ± 10% on average. With a few exceptions (e.g., valsartan, rufinamide, and sulfathiazole, as detailed earlier), these deviations of rate constants fall within the variations observed in repeated experiments and/or across different laboratories, where higher deviations up to ±25% have been noted.^20,44^ Another aspect worth highlighting is that using either SN or AE as resuspension media resulted in nonsignificant differences in both community composition and biotransformation kinetics for the majority of test substances. In contrast, AW, whose macronutrient composition is closer to wastewater influent than AS supernatant, induced changes in the bacterial community beta diversity and its observed biotransformation potential. From a practical point of view, this means that there is no need to freeze large volumes of SN along with cryo samples as AE can be freshly produced when thawing and resuscitating preserved AS pellets. While lyo preservation procedures would be more advantageous to cryo in terms of energy saving and transportation, as lyosamples do not require storage at −80 °C, resuscitated lyocommunities were not representative of fresh AS in terms of biotransformation potential and bacterial community composition. The observed deviations in lyosamples are likely due to significant microbial community loss from freezing and vacuum-drying stresses, insufficiently mitigated by a lyoprotective agent (LPA) like skimmed milk and trehalose. Overall, we conclude that our cryopreservation strategies are good candidates for an effective preservation protocol for maintaining the bacterial community composition and biotransformation potential of AS.

Nevertheless, several caveats need to be addressed. First, the efficiency of the preservation methods was assessed for only 36 test substances. Although they cover a broad range of possible microbial transformation reactions, the selected substances likely do not fully represent the considerable diversity of environmentally relevant MP biotransformation reactions. Furthermore, our results provide some evidence that for MPs that are likely catabolically transformed by specific degraders (e.g., valsartan),^20,31^ the developed preservation methods might not sufficiently preserve biotransformation potential. These phenomena warrant further investigation, e.g., by analyzing the transformation products (TPs) of the investigated MPs to gain insights into their degradation pathways for different preservation methods. Also, protist members of AS are likely diminished during cryo, reducing bioaccumulation through ion trapping in protists. This latter preservation-induced change can be interpreted as revealing the actual extent of microbial biotransformation pathways in AS for amine-containing substances and hence as providing a clearer picture of their biotransformation potential, as previously achieved through inactivation of protists using digitonin.^36^ However, other environments targeted in read-across,^12^ such as soils, might also contain protists. How and whether the loss of protists in cryopreservation hampers read-across for amine-containing substances would therefore need to be addressed further.

A second limitation of our study is that the preservation methods were tested on a single AS microbiome from one WWTP at a specific time, so the results may not be fully generalizable to other AS samples. Nevertheless, this study provides initial insights into how various preservation and experimental factors affect MP biotransformation kinetics, which is crucial information for further optimizing preservation protocols for mixed microbial communities. We tested two storage times (1 and 17 weeks) and observed no significant effect on the biotransformation potential in cryo samples. Based on other studies on the effect of storage duration in the literature, we do not expect that longer storage durations of up to 5 years would change this finding,^21^ although lower freezing temperatures (either −135 °C or even in liquid nitrogen) could be additionally considered to improve the storage conditions. To further address the potential loss of rare and specialized bacteria in AS during preservation, additional refinements of the protocols suggested in the scientific literature could be considered.^21,45^ Possible refinements include concentrating the biomass before preservation and adjusting the TSS after resuscitation. Further, controlled slow cooling rates, snap-freezing in liquid nitrogen, different warming rates, or storage temperatures could be tested to further optimize the recovery of biotransformation potential.

In conclusion, our results suggest that cryopreservation-based protocols appear to be a viable option to preserve the biotransformation potential of AS for a majority of MPs over extended periods of time. Therefore, they should be further explored to support increased reproducibility in persistence assessment, whether in the context of regulatory hazard assessment, persistence screening during the design of novel chemicals, or when developing read-across strategies to derive half-lives in soil.^12^

## Supporting Information Available

Literature review of preservation methods for microbial communities, notes on preservation method development, and preservation media recipes; experimental details for biotransformation experiments; information on additionally quantified experimental variables; and concentration–time series and information on kinetic fits (PDF)
