# Supplementary material for: Preserving the Biotransformation Potential of Activated Sludge in Time: Toward Reproducible Incubation Experiments for Persistence Assessment
Source: Environ Sci Technol. 2025 Feb 25;59(9):4597–607. doi: 10.1021/acs.est.4c08657 (PMC11912309; doi:10.1021/acs.est.4c08657)
Supplement: Supplementary file 1 — es4c08657_si_001.pdf [file es4c08657_si_001.pdf]

Preserving the Biotransformation Potential of Activated Sludge in Time: Towards Reproducible Incubation Experiments for Persistence Assessment

Martina Kalt,[†,‡] Chloé Iris Udressy,[†,¶] Yaochun Yu,[†] Axelle Colliquet,[†,§] and Kathrin Fenner[*,†,‡]

†*Eawag, Swiss Federal Institute of Aquatic Science and Technology, 8600 Dubendorf, Switzerland*

‡*University of Zurich, Department of Chemistry, 8057 Zurich, Switzerland*

¶*ETH Zurich, Department of Environmental Systems Science, 8092 Zurich, Switzerland*

§*ETH Zurich, Department of Chemistry and Applied Biosciences, 8092 Zurich, Switzerland*

E-mail: kathrin.fenner@eawag.ch

Supporting Information

Number of pages: 76

Number of figures: 12

Number of tables: 14

Contents

S1 Selection of Preservation Methods **S5**

S1.1 Literature Review of Preservation Methods for Microbial Communities . . S5

S1.2 Method Development S8

S1.3 Experimental Resuspension Media S10

S2 Biotransformation Experiment **S12**

S2.1 Test Substances S12

S2.2 Preparation of Test Reactors S15

S2.3 Sampling for Test Substance Concentration-Time Series S17

S2.4 Quantification of Test Substance Concentrations S17

S2.5 Sorption Control S19

S3 Assessed Experimental Variables and Measurement Protocols **S21**

S3.1 pH Evolution During Biotransformation Assay S21

S3.2 Ambient Temperature S23

S3.3 VSS and TSS S24

S3.4 Viable Cell Counts from Flow Cytometry S26

S3.5 Community Structure from 16S rRNA Amplicon Gene Sequencing S30

S4 Test Substance Concentration-Time Series **S37**

S5 Biotransformation Profiling **S48**

S5.1 Validation Parameters for First-Order Rate Constants Derivation S48

S5.2 Results of Biotransformation Profiling S48

References **S72**

Glossary **S76**

List of Figures

Fig. S1 Ambient Temperature Recordings S23

Fig. S2 TSS Evolution throughout the Experiment S26

Fig. S3 VSS Evolution throughout the Experiment S27

Fig. S4 Total Growth of Intact Bacterial Cells Measured with Flow Cytometry S30

Fig. S5 OTUs S33

Fig. S6 Shannon Diversity S34

Fig. S7 Genus Diversity at Point in Time -1 h S35

Fig. S8 Concentration-Time Series of the Test Substances S47

Fig. S9 Heatmap with the Averaged Absolute Rate Constants S68

Fig. S10 Heatmap with the Averaged Relative Rate Constants S70

Fig. S11 Count of Relative Rate Constants S71

Fig. S12 Relative Magnitude Shifts of Rate Constants S72

List of Tables

Tbl. S1 Literature Review of Lyophilization of Microbial Communities S6

Tbl. S2 Literature Review of the Cryopreservation of Microbial Communities . S7

Tbl. S3 Content of Artificial Resuspension Media S10

Tbl. S4 Measured Chemical Parameters of Resuspension Media S11

Tbl. S5 Test Substances S12

Tbl. S6 Chemical Structures of Test Substances S13

Tbl. S7 Gradient Flow for Liquid Chromatography of HPLC-MS/MS S19

Tbl. S8 pH Measurements S21

Tbl. S9 Total Suspended Solids (TSS) and Volatile Suspended Solids (VSS) . . S25

Tbl. S10 Intact Cell Concentration Measured with Flow Cytometry S29

Tbl. S11 Results of Pairwise T-Tests on the OTUs S36

Tbl. S12 Results of the Pairwise T-Tests on the Shannon Diversity Index S36

Tbl. S13 Excluded Test Substances S49

Tbl. S14 Absolute Rate Constants S51

S1 Selection of Preservation Methods

S1.1 Literature Review of Preservation Methods for Microbial Communities

To the best of our knowledge, no methodology for preserving an activated sludge (AS) microbiome has been reported in the literature to date. Consequently, the development of long-term preservation approaches for AS was based on a review of conservation methods used for other types of mixed microbial communities. These included a switchgrass-degrading microbial community,[1] methane-oxidizing bacteria (MOB),[2,3] heterotrophic bacteria,[2] autotrophic ammonia-oxidizing bacteria (AOB),[4] oxygen-limited autotrophic nitrification/denitrification (OLAND) biofilm,[2] and human fecal microbiota.[2,5] Microbial preservation studies were primarily screened for the most frequently employed preservation methods, which suggest a successful recovery of a wider range of microorganisms. The criteria for selection included overall performance, adaptability of the technique to the preservation of AS biomass, and ease of reproduction. The two major long-term preservation methods identified were cryopreservation (CRYO) (at −80 °C) and lyophilization (LYO), both with the addition of different protective agents. A comprehensive table summarizing the reviewed literature on the long-term preservation methods of mixed microbial communities is presented below. Despite frequent reports of changes in community structure, several preservation protocols allowed for the successful recovery of microbial activity after preservation. Treatments listed as conclusive are essentially indicative of the potential performance of preservation methods. Defining a successful treatment was indeed challenging considering the high variability in experimental design and recovery assessment across reviewed studies.

Tbl. S1: Literature review of the lyophilization of microbial communities for long-term storage. Conclusive treatments in brackets were evaluated as less effective.

Reference	Preserved microbiome	Recovery evaluation	Lyoprotective solution	Freezing temperature	Storage duration	Conclusive treatments[a]
Yarberry et al.[6]	Anaerobic digestion inoculum for wastes to CH_4 conversion	Activity	**1.** 10 % (w/v) skim milk medium<br>**2.** 10 % (v/v) glycerol<br>**3.** 10 % (v/v) glycerol with 10 % skim milk	−20 °C	1 day to 3 weeks	**1**
Hoefman et al.[4]	MOB[b]	Viability<br>Culturability	**1.** 20 % sucrose/ 10 % BSA[c]<br>**2.** 20 % sucrose/ 1 0% BSA[c]with TT[d]<br>**3.** 7.5 % trehalose in horse serum<br>**4.** 7.5% trehalose in horse serum with TT[d]<br>**5.** 10 % skimmed milk<br>**6.** 12 % glycine betaine	−50 °C	3, 6, 12 months	(**1**), **2**, **3**, **4**, (**5**)
Rothrock et al.[7]	Anammox bacteria[f]	Activity<br>Community structure	**1.** 25 % (v/v) glycerol in skim milk medium<br>**2.** Skim milk medium	−60 °C<br>−200 °C[g]	4 months	**2**
Bellali et al.[5]	Human fecal microbiota	Viability	**1.** Saline solution<br>**2.** Skim milk 10 %, surcrose 10 %, trehalose 5 % and antioxidants	−80 °C	30 days	**2**

[a] Culturability not taken into account.
[b] Methane-oxidizing bacteria.
[c] Bovine serum albumin.
[d] Ten-fold diluted trypticase soy broth medium supplemented with 1 % trehalose.
[e] Conclusive treatment: $\geq$ 80 % of viability retained after preservation.
[f] Anaerobic ammonium-oxidizing bacteria.
[g] Liquid nitrogen.

Tbl. S2: Literature review of the cryopreservation of microbial communities for long-term storage. Not necessarily all combinations of preservation media and cryoprotective agents were tested as cryoprotective solutions. Conclusive treatments in brackets were evaluated as less effective.

Reference	Preserved microbiome	Recovery evaluation	preservation medium	CPA	Freezing temperature	Storage duration	Conclusive treatments[a]
Yu et al.[1]	Switchgrass-degrading microbial community	Activity community structure	**1.** Distilled-deionized water	**A.** 10 % (v/v) glycerol<br>**B.** 5 % (v/v) DMSO<br>**C.** None	−80 °C	3 weeks	**1A, 1B**
Kerckhof et al.[2]	MOB[b] and heterotrophs<br>OLAND[c] biofilm<br>Human fecal microbiota	Activity community structure	**1.** Distilled autoclaved tap water<br>**2.** TT[d] medium	**A.** 5 % (v/v) DMSO<br>**B.** None	−80 °C	3 months	(**1A**), **2A** for all. **1B** for fecal biota.
Hoefman et al.[4]	AOB[e]	Activity	**1.** Std growth medium<br>**2.** Std growth medium with TT[d]	**A.** 5 % (v/v) DMSO	−80 °C<br>−200 °C[f]	3 months	(**1A**), **2A**
Hoefman et al.[3]	MOB[b]	Viability<br>Culturability	**1.** Std growth medium<br>**2.** TT[d] medium	**A.** 15 % (v/v) glycerol<br>**B.** 5 % (v/v) DMSO<br>**C.** 10 % MeOH<br>**D.** 20 % sucrose<br>**E.** 12 % glycine betaine<br>**F.** Microbank beads	−80 °C<br>−200 °C[f]	3, 6, 12 months	**1A**, **1B**, (**1C**), (**1D**), **1E**, **2A**, **2B**[h]
Heylen et al.[8]	Anammox bacteria[i]	Activity	**1.** Mineral medium<br>**2.** Mineral medium with TT[d]<br>**3.** Mineral medium with 1 % trehalose	**A.** 5 % (v/v) DMSO<br>**B.** None	−80 °C	29 weeks	(**1A**), **2A**, **3A**
Laurin et al.[9]	Denitrifying microbial biofilm from the Montreal Biodome	Activity<br>Community structure	**1.** Artificial seawater	**A.** 15 % (v/v) glycerol<br>**B.** None	−20 °C<br>−80 °C	1 week, 1, 6, 17 months	**1A**
Rothrock et al.[7]	Annamox bacteria[i]	Activity<br>Community structure	**1.** Skim milk medium	**A.** 25 % (v/v) glycerol<br>**B.** None	−60 °C	4 months	**1A**, **1B**

[a] Culturability not taken into account.
[b] Methane-oxidizing bacteria.
[c] Oxygen limited autotrophic nitrification/denitrification.
[d] 0.3 % trypticase soy broth supplemented with 1 % trehalose.
[e] Autotrophic ammonia-oxidizing bacteria.
[f] Liquid nitrogen.
[g] Ten-fold diluted trypticase soy broth medium supplemented with 1 % trehalose.
[h] Conclusive treatment: ≥ 80 % of viability retained after preservation.
[i] Anaerobic ammonium-oxidizing bacteria.

S1.2 Method Development

S1.2.1 Lyophilization

In order to protect cells against LYO stresses, lyoprotective agents (LPAs) were added to AS pellets before the freezing and desiccation steps. A mixture of powdered skimmed milk and trehalose dissolved in autoclaved groundwater was selected for this purpose. Commonly used for the LYO of axenic cultures,[10] skimmed milk was frequently reported to be efficient at preserving the activity, viability and/or community structure of microbiomes by surrounding microbial cells with a protective viscous layer.[4–7] Since a combination of LPAs was shown to provide a greater protection of microorganisms due to potential additive or synergistic protective effects,[4] trehalose, one of the most common disaccharides used as LPAs, was additionally included in the LYO solution.[5] As a non-permeating protective agent similar to skimmed milk, trehalose was suggested to protect cells from freezing and vacuum-drying stresses by stabilizing cell membranes and proteins as well as inhibiting excessive intracellular ice formation by means of osmotic dehydration.[5,6] Concentrations of skimmed milk (12 % w/w) and trehalose (7 % w/w) were determined based on typical protective suspension media used for the protection of cells against damage during LYO referenced by the Common Access to Biological Resources and Information (CABRI) project.[11] A dosage of 2.5 mL of LYO solution per gram wet weight of biomass was selected according to the range of concentrations reported in literature (2.5 – 5 $\mathrm{mL\,g^{-1}}$ wet weight of biomass).[5–7]

S1.2.2 Cryopreservation

The most commonly used cryoprotective agents (CPAs) for microorganisms preservation are dimethyl sulfoxide (DMSO) and glycerol (GLY), usually employed in concentrations of 5 – 10 % and 10 – 15 %, respectively.[12] These cell-permeating CPAs are reported to protect microbial cells by lowering the freezing point of water and preventing excessive dehydration by increasing intracellular solute concentration.[10,13] DMSO additionally inhibits intracellular

ice formation and osmotic stress by increasing membrane permeability, which in turn, facilitates water and solute transport across cell membranes.[14] As these CPAs were successfully assessed for the preservation of a wide range of mixed microbial communities,[1–4,7–9] both DMSO (5 % (v/v)) and GLY (10 % (v/v)) were tested for the CRYO of an AS microbial community. The preservation medium in which each CPA was diluted was identical to the growth medium that was later used to resuspend concentrated biomass for biotransformation assays, i.e. either filtered activated sludge supernatant (SN), artifical wastewater (AW), or artifical effluent (AE). These preservation media were chosen since a carbon-rich medium, for e.g. trypticase soy broth supplemented with trehalose (TT), was previously suggested to be more successful at preserving oligotrophic and/or fastidious microorganisms.[2,4,8] AS biomass was mixed with the cryoprotective solution (combination of preservation medium and CPA) at an identical dosage to the two sole studies which specified this detail (5 mL of cryoprotective solution per gram wet weight of biomass).[2,7] The duration between cryoprotective solution addition and freezing of samples was dependent on the type of CPA. Whereas the biomass was left to equilibrate with the added GLY solution for at least 60 min to allow for cellular uptake,[1–3] DMSO treated samples were immediately frozen after addition of the cryoprotective solution as a preventive measure against its potential cytotoxicity. At high concentrations, DMSO was indeed reported to disintegrate the bilayer structure of lipid membranes[14] - an adverse effect which becomes greater as temperature increases.[15]

S1.3 Experimental Resuspension Media

S1.3.1 Artificial Media Composition

Artificial wastewater (AW) and artificial effluent (AE) were synthetically produced from salts, proteins, yeast and carbohydrates according to the composition detailed in Table Tbl. S3. AW composition was adopted from Seller et al.,[16] AE composition adjusted accordingly to mimic native supernatant (SN).

Tbl. S3: Table of composition of the artifical media used in the experiments.

**substance**	**Abb.**	**Conc. AW** [$mg\,L^{-1}$]	**Conc. AE** [$mg\,L^{-1}$]
Sodium bicarbonate	$NaHCO_3$	1050	1050
Sodium acetate	$C_2H_3NaO_2$	196	196
Peptone		196	-
Hydrolyzed yeast extract		196	-
Ammonium chloride	NH_4Cl	174	230
Starch		70	-
Dipotassium hydrogen phosphate	K_2HPO_4	59	60
Potassium dihydrogen phosphate	KH_2PO_4	37	-
Magnesium sulfate	$MgSO_4$	16.5	22

Abb: abbreviation, Conc: concentration, AW: artifical wastewater, AE: artifical effluent.

S1.3.2 Physico-Chemical Characterization of Resuspension Media

Filtered SN (thawed after storage at $-20\,°C$), AW, and AE were analyzed by the AuA laboratory at Eawag. Samples were filtered ($0.45\,\mu m$) prior to the evaluation to remove turbidity and precipitation.

Tbl. S4: Table of chemical parameters for the media used in the experiments.

**Parameter**	**Abb**	**Unit**	**SN**	**AW**	**AE**
Conductivity		$\mu S\,cm^{-1}$, 20 °C	959.4	1910.9	1381
pH			8.92	8.12	8.14
alkalinity		$mmol\,L^{-1}$	4.907	17.216	8.92
sodium	Na^{+}	$mg\,L^{-1}$	129.489	353.336	174
magnesium	$Mg_2{}^{+}$	$mg\,L^{-1}$	17.32	13.411	17.8
calcium	$Ca_2{}^{+}$	$mg\,L^{-1}$	61.483	43.751	32.8
potassium	K^{+}	$mg\,L^{-1}$	19.829	46.594	21.7
fluoride	F^{-}	$mg\,L^{-1}$	0.124	*NA*	*NA*
chloride	Cl^{-}	$mg\,L^{-1}$	181.693	136.098	165
bromide	Br^{-}	$mg\,L^{-1}$	*NA*	*NA*	0.07
nitrate	$NO_3{}^{-}$	$mg\,L^{-1}$	0.144	3.2	2.729
sulfate	$SO_4{}^{2-}$	$mg\,L^{-1}$	37.992	27.84	38
ammonium	$NH_4{}^{+}$	$mg\,L^{-1}$	0.838	63	55.45
phosphate	$PO_4{}^{3-}$	$mg\,L^{-1}$	1.254	17.8825	1.321
dissolved phosphorus	P	$mg\,L^{-1}$	1.315	20.725	8.1
total phosphorus	P	$mg\,L^{-1}$	2.345	20.725	8.1
silicic acid	H_4SiO_4	$mg\,L^{-1}$	14.57	11.774	8.2
dissolved organic carbon	DOC, C	$mg\,L^{-1}$	1.208	32	9.5
total organic carbon	TOC, C	$mg\,L^{-1}$	1.446	32	5
total nitrogen	N	$mg\,L^{-1}$	*NA*	55	*NA*
total inorganic carbon	TIC, C	$mg\,L^{-1}$	58.884	206.592	62.9

Abb: Abbreviation, SN: supernatant, AW: artifical wastewater, AE: artifical effluent, *NA*: not applicable, e.g., below level of quantification.

S2 Biotransformation Experiment

S2.1 Test Substances

Tbl. S5: List of the 36 study substances characterized by their Chemical Abstracts Service (CAS) number, substance class, and assigned stable-isotope-labeled internal standard (SIL-IS).

Substance	CAS	Class	Sub-class	SIL-IS
Acemetacin	53164-05-9	PH	Analgesic, anti-inflammatory	Diclofenac-D4
Acesulfame	33665-90-6	HI	Artificial sweetener	Acesulfame-D4
Alachlor	15972-60-8	PE	Herbicide	Alachlor-D13
Albuterol	18559-94-9	PH	Bronchodilator	Albuterol-D4
Atenolol	29122-68-7	PH	β-blocker	Atenolol-D7
Atorvastatin	134523-00-5	PH	Lipid-lowering agent	Atorvastatin-D5
Atrazine	1912-24-9	PE	Herbicide	Atrazine-D5
Bezafibrate	41859-67-0	PH	Lipid-lowering agent	Bezafibrate-D4
Carbendazim	10605-21-7	PE	Fungicide	Carbendazim-D4
Chlorotoluron	15545-48-9	PE	Herbicide	Chlorotoluron-D6
Climbazole	38083-17-9	PE	Fungicide	Climbazole-D4
DEET	134-62-3	PE	Insect repellent	DEET-D10
Dextromethorphan	125-71-3	PH	Cough suppressant	Eprosartan-D3
Diclofenac	15307-86-5	PH	Analgesic, anti-inflammatory	Diclofenac-D4
Dimethenamid	87674-68-8	PE	Herbicide	Dimethenamid-D3
Emtricitabine	143491-57-0	PH	Nucleoside reverse-transcriptase inhibitor	Emtricitabine-13C,15N2
Furosemide	54-31-9	PH	Diuretic	Furosemide-D5
Ketamine	6740-88-1	PH	Anesthetic	Ketamine-D4
Ketoprofen	22071-15-4	PH	Analgesic, anti-inflammatory	Atrazine-D5
Losartan	114798-26-4	PH	Anti-hypertensive	Losartan-D4
Mecoprop	93-65-2	PE	Herbicide	Mecoprop-D6
Metoprolol	37350-58-6	PH	β-blocker	Metoprolol-D7
Mexiletine	31828-71-4	PH	Anti-arrhythmic	Metoprolol-D7
Pravastatin	81093-37-0	PH	Lipid-lowering agent	Pravastatin-D3
Propachlor	1918-16-7	PE	Herbicide	Atrazine-D5
Ranitidine	66357-35-5	PH	Gastric antacid	Ranitidine-D6
Rufinamide	106308-44-5	PH	Anticonvulsant	Pirimicarb-D6
Sitagliptin	486460-32-6	PH	Anti-diabetic	Sitagliptin-D4
Sulfadiazine	68-35-9	PH	Sulfonamide antibiotic	Sulfadiazine-D4
Sulfamethazine	57-68-1	PH	Sulfonamide antibiotic	Sulfamethazine-13C6
Sulfamethoxazole	723-46-6	PH	Sulfonamide antibiotic	Sulfamethoxazole-D4
Sulfapyridine	144-83-2	PH	Sulfonamide antibiotic	Sulfapyridine-D4

Continued on next page

**Substance**	**CAS**	**Class**	**Sub-class**	**SIL-IS**
Sulfathiazole	72-14-0	PH	Sulfonamide antibiotic	Sulfathiazole-D4
Tramadol	27203-92-5	PH	Analgesic	Tramadol-D6
Valsartan	137862-53-4	PH	Anti-hypertensive	Valsartan-13C5-15N
Venlafaxine	93413-69-5	PH	Antidepressant	Venlafaxine-D6

HI: household and industrial chemical, PE: pesticide, PH: pharmaceutical.

Tbl. S6: Chemical structures of the 36 test substances

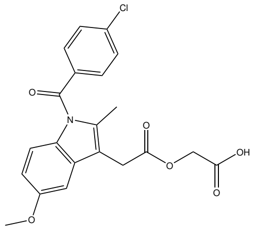 acemetacin	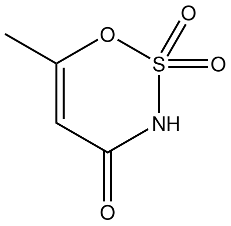 acesulfame	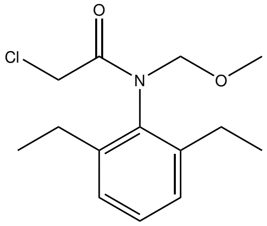 alachlor
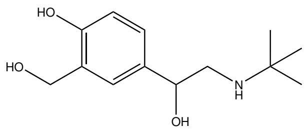 albuterol	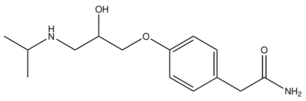 atenolol	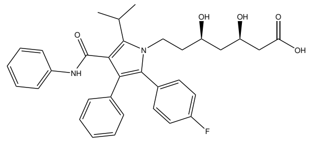 atorvastatin
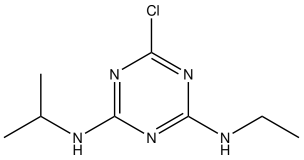 atrazine	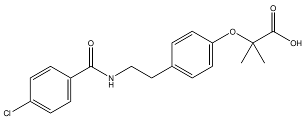 bezafibrate	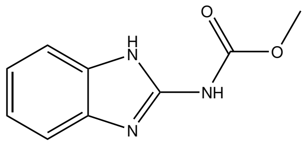 carbendazim
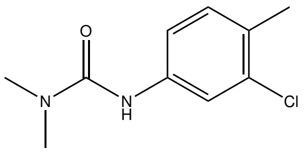 chlorotoluron	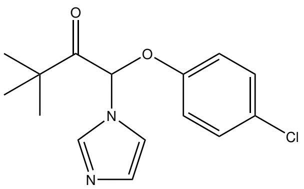 climbazole	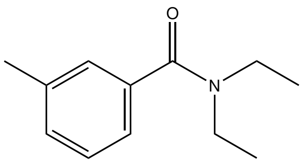 DEET

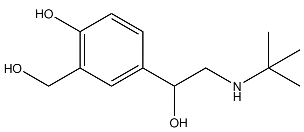 albuterol	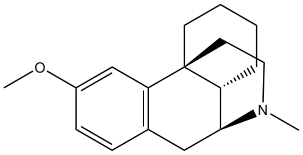 dextromethorphan	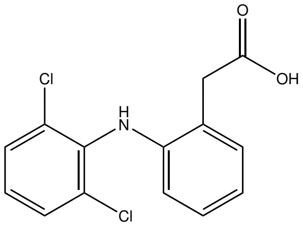 diclofenac
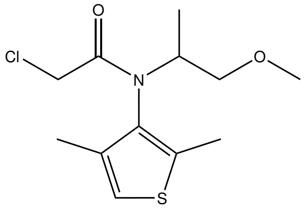 dimethenamid	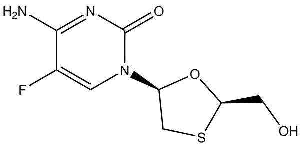 emtricitabine	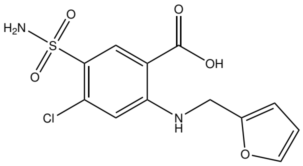 furosemide
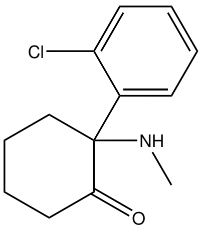 ketamine	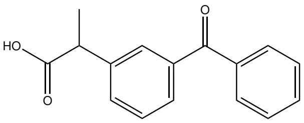 ketoprofen	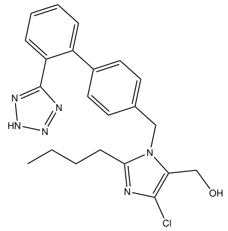 losartan
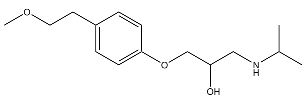 metoprolol	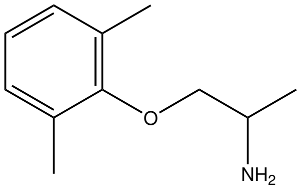 mexiletine	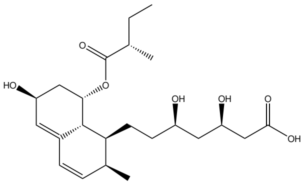 pravastatin
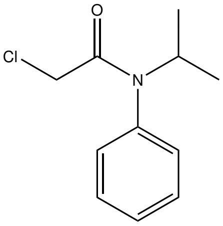 propachlor	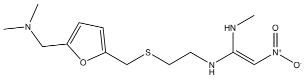 ranitidine	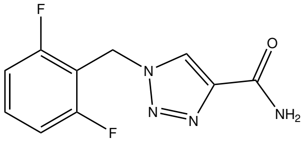 rufinamide

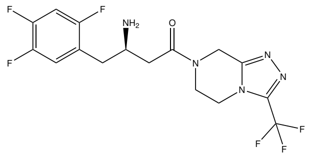 sitagliptin	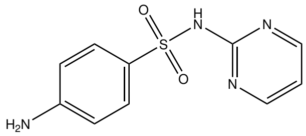 sulfadiazine	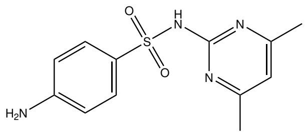 sulfamethazine
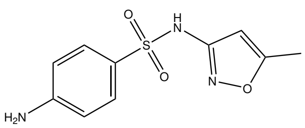 sulfamethoxazole	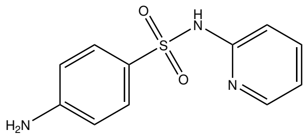 sulfapyridine	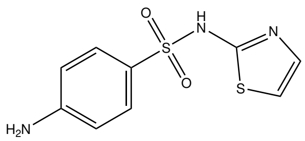 sulfathiazole
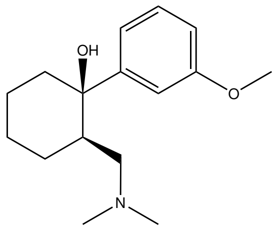 tramadol	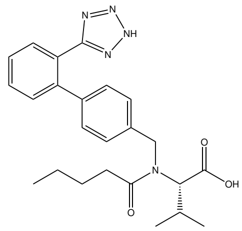 valsartan	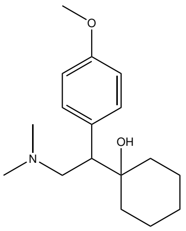 venlafaxine

S2.2 Preparation of Test Reactors

S2.2.1 Sampling of Activated Sludge

AS was collected on 8 May 2023 from the aerated nitrifying treatment basin of ARA Neugut (Dübendorf, ZH, Switzerland), a wastewater treatment plant (WWTP) performing biological phosphate elimination, nitrification and denitrification. This WWTP treats the wastewater of approximately 105,000 inhabitant equivalents, with domestic and industrial inputs respectively accounting for approximately 48 % and 52 % of the inflow. After sampling, AS was transported in under 30 min to the laboratory. Until being aliquoted for preservation or for the first biotransformation experiment, native activated sludge was stirred with an open lid at a maximum speed of 150 rpm in order to avoid grinding the AS flocs. Besides pH

measurement, samples were taken for determination of total suspended solids (TSS), volatile suspended solids (VSS), viability of cells and community structure.

S2.2.2 Pre-processing of Native Activated Sludge

As AS conservation required mixing concentrated biomass with protective solutions prior to CRYO and LYO, the preservation procedure was initiated by centrifuging native AS (4000 rcf, 5 min) and setting aside the supernatant for later resuspension use. This preliminary step was also included in the preparation of fresh AS suspensions to maintain handling similarity and to be able to test different growth media in incubation assays. The centrifugation of AS was directly performed in aliquots of 50 mL to have one individual falcon tube for the seeding of each batch reactor for biotransformation experiments. As the determination of TSS, VSS, cell viability and community structure jointly required a large sampling volume relative to the vessel content, each treatment was prepared in quadruplicates: three intended for incubation assays and an additional one solely prepared for pre-experimental sampling.

S2.2.3 Reactivation of Preserved Activated Sludge

The preserved samples were reactivated for the second and third biotransformation experiments after one week and 17 weeks of storage, respectively. Cryopreserved samples were thawed at ambient temperature. Because of the potential cytotoxicity of DMSO, mixtures were directly centrifuged upon thawing (4000 rcf, 5 min) to discard the cryoprotective solution. Concentrated biomass was resuspended in 50 mL of autoclaved groundwater, homogenized by shaking vigorously and centrifuged again (4000 rcf, 5 min) before discarding the supernatant. This rinsing procedure was performed two times to ensure a thorough removal of the cryoprotective solution. The lyophilized samples were rehydrated by adding 50 mL of autoclaved groundwater. Post 1 h at RT, the lyophilized suspensions were centrifuged (4000 rcf, 5 min). After supernatant removal, activated sludge pellets were rinsed to remove any potential residual lyoprotective solution by following the same procedure as for the

resuscitation of cryopreserved samples.

S2.2.4 Preparation of Activated Sludge Suspensions in Batch Reactors

Final AS suspensions for incubation assays were prepared by resuspending fresh and preserved AS pellets in 50 mL corresponding growth media: filtered activated sludge supernatant (SN), artificial wastewater (AW), and artificial effluent (AE). SN was retrieved from the centrifugation of native AS and was passed through a 0.2 µm membrane filter to remove any potential remaining biomass. Whereas fresh SN was added to fresh biomass for the first biotransformation assay, preserved AS pellets were mixed with SN stored at −20 °C from the pre-processing of native AS until the reactivation of preserved AS suspensions. After thorough homogenization, each 50 mL AS suspension was transferred to a 100 mL Schott bottle placed on an orbital shaker operating at 150 rpm at RT. Batch reactors were let to equilibrate with perforated lids overnight (20 h).

S2.3 Sampling for Test Substance Concentration-Time Series

1.5 mL of homogenized AS suspension was sampled from the batch reactors using a muffled glass Pasteur pipette. After centrifugation (21 130 rcf, 15 min, 4 °C), 1000 µL of supernatant was transferred to an amber glass HPLC vial into which 20 µL of ISTD mixture was subsequently spiked (final concentration 2 $\mu g\,L^{-1}$). The mixture was shaken vigorously and immediately stored at −20 °C. Shortly before measuring, the samples were thawed and centrifuged (4000 rcf, 15 min, 20 °C). A volume of 900 µL of supernatant was transferred into a new HPLC vial for analysis.

S2.4 Quantification of Test Substance Concentrations

Test substance quantification was performed by a Thermo Scientific™ UltiMate™ 3000 UHPLC System (Thermo Fisher Scientific) coupled to a Thermo Scientific™ Q Exactive™ Plus Hybrid 11 Quadrupole-Orbitrap™ mass spectrometer (Thermo Fisher Scientific). A volume

of 100 µL of sample was injected in a mobile phase constituted of ultrapure water (Arium® Pro, Sartorius) and methanol (MeOH, Optima™ LC/MS Grade, Fisher Scientific), each supplemented with 0.1 % formic acid (98 – 100 %, Merck KGaA). The eluate was injected to an Atlantis T3 column (150 x 3.0 mm, C18, particle size 3 µm, Waters) with pre-column (3.9 x 5.0 mm, 3 µm, Waters) at a flow rate of 0.3 mL min^{-1} under gradient conditions (30 min total gradient time). The evolution of the MeOH:ultrapure water ratio is detailed in Tbl. S7. The interface between LC and MS was composed of electrospray ionization (ESI) performed at a spray voltage of 4 kV and at a capillary temperature of 320 °C, sheath gas 32 L min^{-1}, s-lens RF level 50.0; full scan MS with data dependent MS2; mass calibration < 0.5 ppm accuracy in (+/−)-ESI with in-house calibration solution. Full mass spectra were acquired in positive/negative switching mode in the range of 100 – 1,000 m/z at a resolution of 75,000. Data dependent MS2 spectra were recorded at a resolution of 17,500 based on an inclusion list. Eleven calibration standards with concentrations of 0.01, 0.05, 0.1, 0.5, 1, 3, 5, 8, 10, 20, and 50 nM were prepared in Evian water. Evian water was used for its higher ionic strength compared to nanopure water to avoid sorption of polar compounds to glass vials. An internal standard mix of micropollutants (ISTD) was produced from stable-isotope-labeled internal standards (SIL-IS, purity mostly above 99 %, main suppliers: Sigma-Aldrich, TCI Europe, LGC standards, HPC standards, and Lipomed AG) combined in EtOH at final concentrations of 100 µg L^{-1}. SIL-IS assigned to each test substance are specified in Tbl. S5. Activated sludge samples, calibration standards and blind samples (Evian water) were spiked with the ISTD at final analyte concentrations of 2 µg L^{-1}. Blank samples (ultrapure water) were additionally interposed in the measurement sequence. LC-MS measurements were evaluated using TraceFinder™ 5.1 (Thermo Fisher Scientific, 2021). Automatic integrations of chromatographic peaks were manually validated by reviewing peak resolution, peak shape, retention time and identification of MS/MS fragments. For each test substance, the most diluted calibration standard exhibiting a sufficiently resolved chromatographic peak was regarded as limit of quantification (LOQ).

Tbl. S7: Contribution of methanol (MeOH) and ultrapure water to the mobile phase of the liquid chromatography of HPLC-MS/MS for micropollutant quantification (constant flow rate of 0.3 mL min^{-1}).

**Time** [min]	**0**	**1**	**17**	**25**	**25.1**	**30**
**Ultrapure water** [%]	95	95	5	5	95	95
**MeOH** [%]	5	5	95	95	5	5

S2.5 Sorption Control

S2.5.1 Methods and Sampling of Sorption Control Samples

For the sorption control (SC) experiment, six batch reactors with fresh AS were prepared. Triplicates of AS pellets were resuspended in 50 mL SN or AW, respectively, in 100 mL Schott bottles and incubated together with the fresh biotransformation experiment overnight with perforated lids on an orbital shaker operating at 150 rpm at RT. The next morning, the AS suspensions prepared for SCs were tightly closed with non-perforated lids and autoclaved (121 °C, 2 bar, 20 min). The batch reactors were then allowed to cool to ambient temperature and spiked with the same mix of test substances as the biotransformation experiments at an initial concentration of 10 nM. Samples were collected from the reactors at −1 h (before spiking), 0 h (immediately after spiking), and at 4, 24, 48, and 72 h. The samples were centrifuged (21 130 g, 15 min, 4 °C), and 1000 µL of the supernatant was transferred to an amber glass HPLC off-line vial, into which 20 µL of ISTD mixture was subsequently spiked (final concentration: 2 µg L^{-1}). The mixture was shaken vigorously and immediately stored at −20 °C until analysis. The analysis was performed following the measuring protocol S2.4.

S2.5.2 Parameter Derivation of Sorption Control Samples

As the sorption reactors might have been contaminated during sampling, only the points in time up to 48 h were taken into account. If a test substance showed more than 20 % depletion in the aqueous phase of the SC experiment at any sampling point up to 48 h — meaning the concentration of the test substance fell below 8 nM (80 % of the initial spike

concentration) — the substance was excluded from further kinetic evaluation.

S2.5.3 Results of Sorption Control Samples

Included as a sorbing control substance, climbazole reached an average depletion greater than 25 % after 48 h in SC experiments. The same behaviour was detected for acemetacin in the experiments using AW as resuspension media. As such, the corresponding biotransformation rate was not further evaluated. None of the other test substances showed significant sorption behaviour according to the above criteria in the experimental matrices.

S3 Assessed Experimental Variables and Measurement Protocols

S3.1 pH Evolution During Biotransformation Assay

Tbl. S8: Measurements of pH during biotransformation assays

**Treatment**	**Replicate**	0 h	24 h	48 h
fresh_fresh_SN	1	7.52	8.00	8.13
	2	7.73	8.18	8.34
	3	7.57	7.99	8.15
fresh_fresh_AW	1	7.90	8.51	8.63
	2	7.93	8.52	8.65
	3	7.96	8.49	8.62
short_lyo_SN	1	7.72	7.76	8.08
	2	7.69	7.72	8.06
	3	7.68	7.89	8.06
short_lyo_AW	1	8.09	8.71	8.62
	2	8.09	8.69	8.58
	3	8.11	8.74	8.63
short_DMSO_SN	1	7.56	8.00	8.06
	2	7.54	7.95	7.96
	3	7.54	7.96	7.95
short_DMSO_AW	1	8.31	8.81	8.84
	2	8.31	8.82	8.85
	3	8.06	8.63	8.68
short_gly_SN	1	7.67	8.02	8.10
	2	7.61	8.00	8.04
	3	7.75	8.13	8.15
short_gly_AW	1	8.05	8.66	8.70
	2	8.03	8.67	8.70
	3	8.02	8.65	8.68
long_lyo_SN	1	7.89	8.08	8.12
	2	7.69	8.06	8.23
	3	7.87	8.06	8.31
long_lyo_AE	1	8.16	8.71	8.69
	2	8.09	8.69	8.71

Continued on next page

**Treatment**	**Replicate**	0 h	24 h	48 h
	3	8.27	8.69	8.69
long_DMSO_SN	1	7.64	8.01	8.06
	2	7.54	7.95	7.96
	3	7.56	7.98	7.95
long_DMSO_AE	1	8.14	8.26	8.34
	2	8.25	8.34	8.24
	3	8.24	8.54	8.61
long_gly_SN	1	7.56	8.02	8.10
	2	7.61	8.00	8.04
	3	7.64	8.13	8.24
long_gly_AE	1	8.06	8.66	8.54
	2	8.09	8.67	8.64
	3	8.02	8.65	8.59
fresh_sorption_SN	1	8.64	8.66	8.44
	2	8.63	8.60	7.39
	3	8.64	8.62	8.56
fresh_sorption_AW	1	9.43	9.60	7.25
	2	9.44	9.59	9.49
	3	9.43	9.58	9.53

S3.2 Ambient Temperature

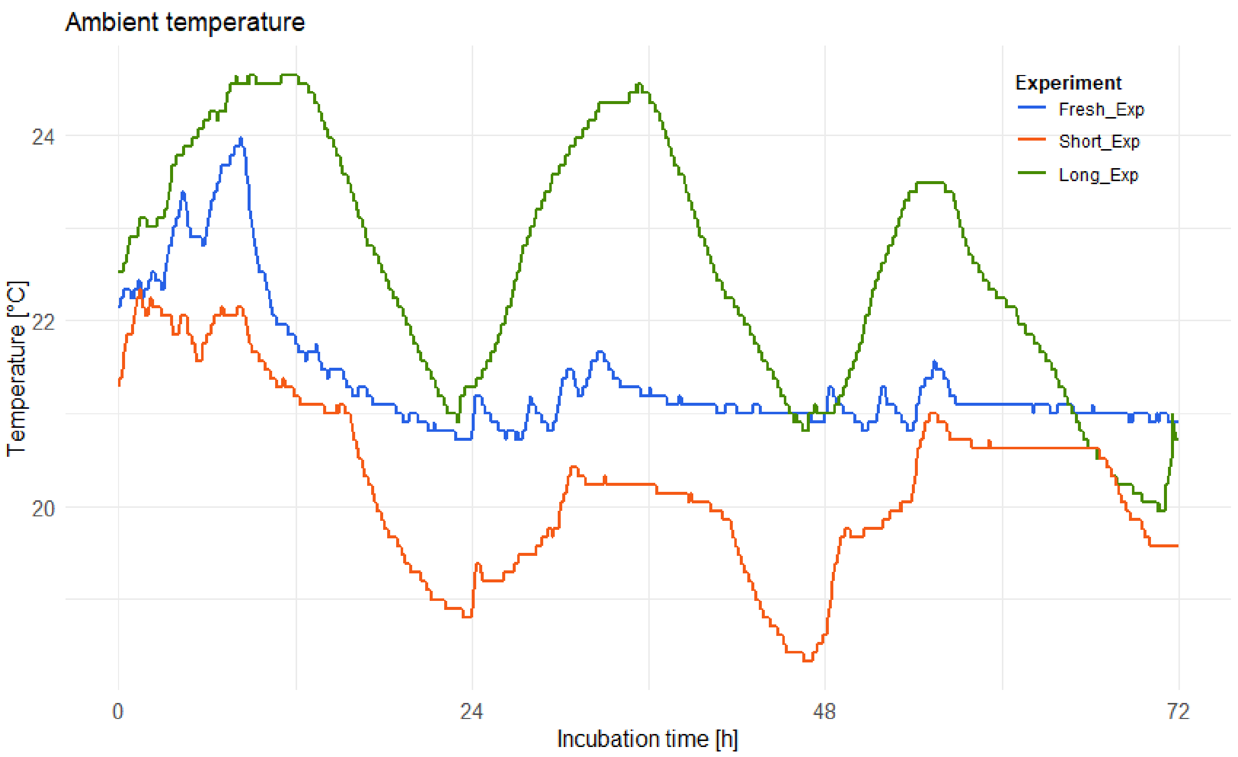

Fig. S1: Ambient temperature recorded throughout biotransformation assays every 5 min.

S3.3 VSS and TSS

S3.3.1 Sampling and Measurement for VSS and TSS

55 mm diameter filters (0.45 µm pore size) were equilibrated in a desiccator. A defined volume of AS suspension (10 mL) was withdrawn from each batch reactor and filtered through the pre-weighed filter. Filters were dried in an oven (105 °C, 1 h) and left to equilibrate in the desiccator before being weighed again (dry mass of organic and mineral residues). Filters were wrapped in aluminum foil and muffled (1 h 20 min heating up to 450 °C followed by 1 h at 450 °C). After another equilibration step in the desiccator, filters were weighed a third time (dry mass of mineral residues).

S3.3.2 Parameter Derivation for VSS and TSS

The first biomass drying step determined the concentration of organic and mineral particles in solution (total suspended solids, TSS). Subsequently, the concentration of undissolved organic matter (volatile suspended solids, VSS) was derived by subtracting the dry mass of mineral residues form the dry sample mass:

$$TSS = \frac{m_d - m_f}{V} \quad (1)$$

$$VSS = \frac{(m_d - m_f) - (m_m - m_f)}{V} \quad (2)$$

where m_f denotes the dry mass of filter alone, m_d the dry mass of filter and organic and mineral residues, m_m the dry mass of filter and mineral residues and V the volume of filtered solution.

S3.3.3 Results for VSS and TSS

Tbl. S9: Measurements of total suspended solids (TSS) and volatile suspended solids (VSS) before ($-1\,h$) and after ($72\,h$) the biotransformation assay as a mean of triplicates.

**Treatment**	**TSS** $-1\,\mathrm{h}$ $[\mathrm{g\,L^{-1}}]$	**TSS** $72\,\mathrm{h}$ $[\mathrm{g\,L^{-1}}]$	**VSS** $-1\,\mathrm{h}$ $[\mathrm{g\,L^{-1}}]$	**VSS** $72\,\mathrm{h}$ $[\mathrm{g\,L^{-1}}]$
fresh_fresh_SN	2.72	2.48 ± 0.08	2.36	2.16 ± 0.05
fresh_fresh_AW	3.17	2.94 ± 0.06	2.68	2.44 ± 0.04
short_lyo_SN	3.08	2.95 ± 0.14	2.41	2.95 ± 0.03
short_lyo_AW	3.13	3.00 ± 0.07	2.62	2.45 ± 0.03
short_DMSO_SN	2.85	2.62 ± 0.03	2.45	2.32 ± 0.03
short_DMSO_AW	3.38	2.73 ± 0.10	2.84	2.33 ± 0.07
short_gly_SN	2.65	2.60 ± 0.08	2.32	2.34 ± 0.06
short_gly_AW	3.18	2.92 ± 0.09	2.74	2.55 ± 0.08
long_lyo_SN	2.83	2.48 ± 0.02	2.30	2.19 ± 0.04
long_lyo_AE	2.98	2.92 ± 0.2	2.46	2.54 ± 0.17
long_DMSO_SN	3.09	2.77 ± 0.12	2.41	2.44 ± 0.10
long_DMSO_AE	3.07	2.86 ± 0.25	2.45	2.49 ± 0.21
long_gly_SN	3.17	2.88 ± 0.07	2.54	2.51 ± 0.08
long_gly_AE	3.01	3.07 ± 0.20	2.54	2.51 ± 0.08
fresh_sorption_SN	2.07	2.03 ± 0.03	1.71	1.70 ± 0.03
fresh_sorption_AW	2.47	2.39 ± 0.16	1.92	1.93 ± 0.07

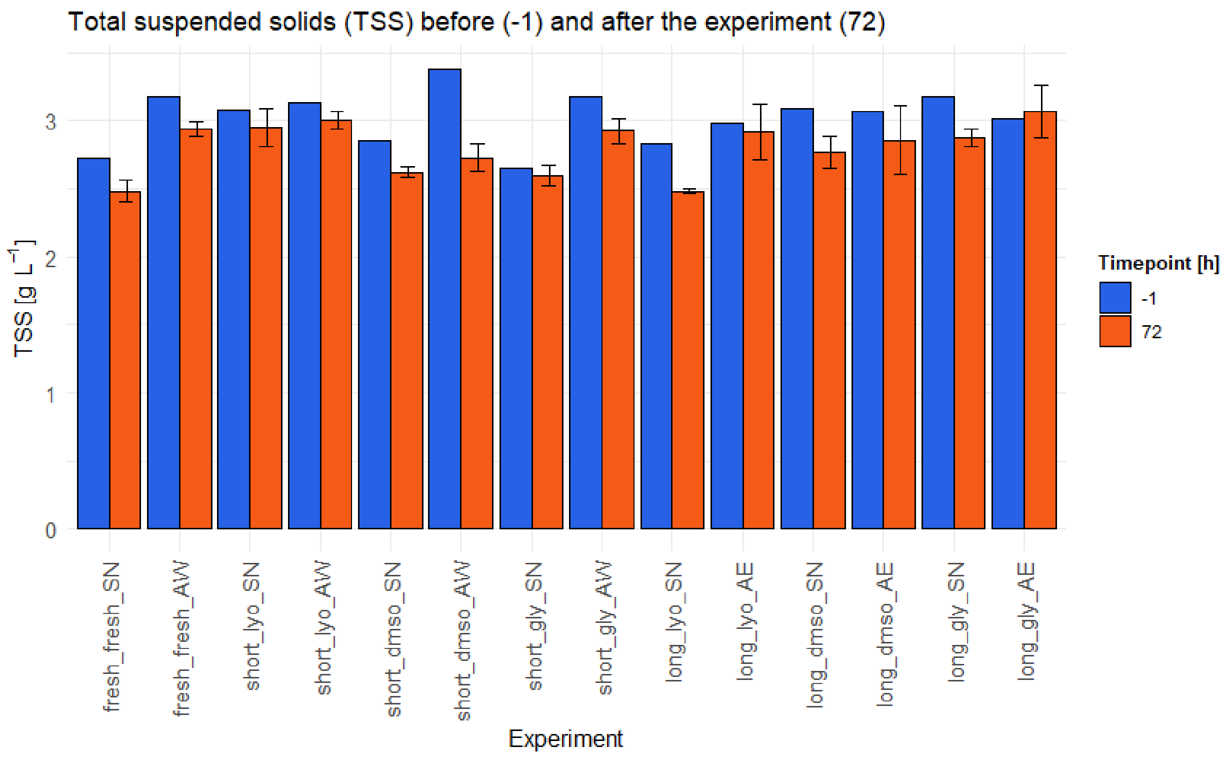

Fig. S2: TSS evolution [g L^{-1}] over the time span of the biotransformation experiment (72 h) as a mean of triplicates.

S3.4 Viable Cell Counts from Flow Cytometry

S3.4.1 Sampling for Flow Cytometry

0.5 mL of AS suspension was added to 0.5 mL of buffer solution (4 % paraformaldehyde, 0.2 % sodium pyrophosphate in ultrapure water) in a glass vial. Samples were stored at 4 °C until measurement.

S3.4.2 Measurement with Live/Dead Flow Cytometry

Samples were shaken vigorously, placed in crushed ice and sonicated with an ultrasonic probe sonicator for two rounds of 5 s in order to detach bacterial cells from AS flocs. After thorough homogenization, samples were further diluted 18,500 fold in 0.2 µm filtered Evian water in order to reach an optimal event rate during FC measurements (below 3000 events s^{-1}). A working SYBR® Green I (SG) staining solution was prepared by diluting stock

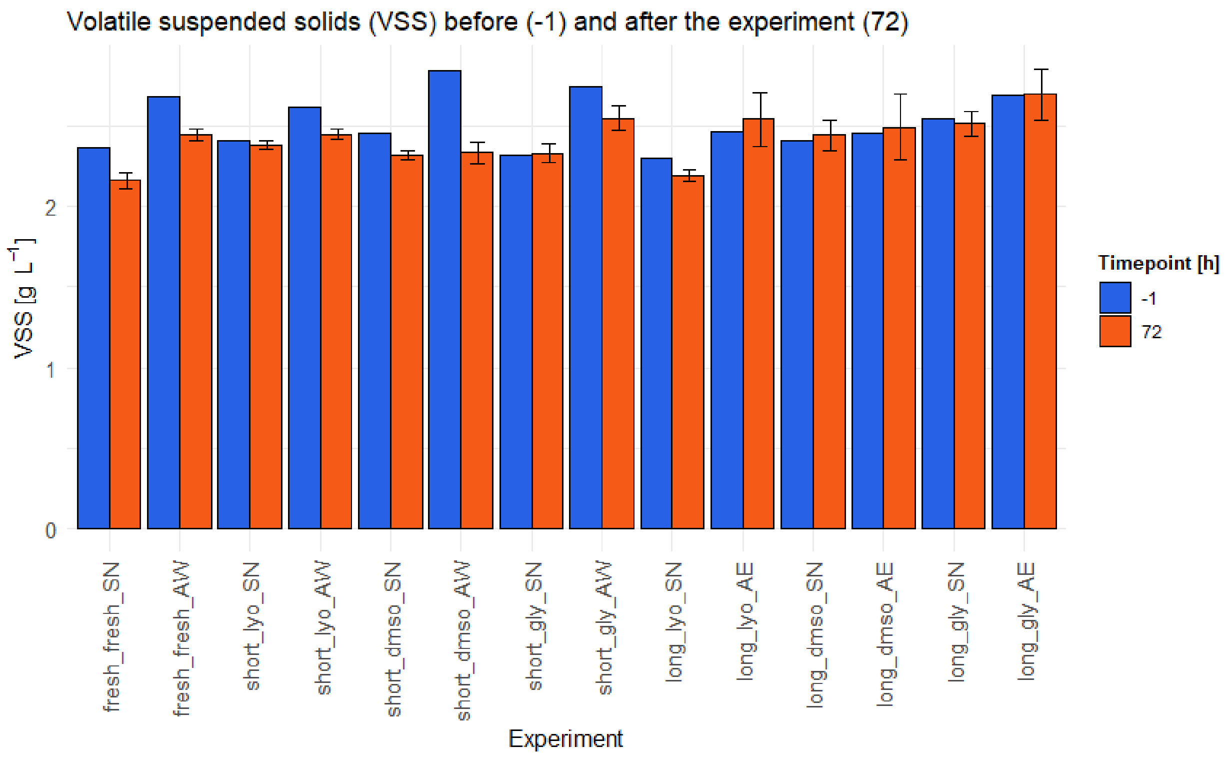

Fig. S3: VSS evolution [$g\,L^{-1}$] over the time span of the biotransformation experiment (72 h) as a mean of triplicates.

dye (Invitrogen AG, Basel, Switzerland) 100 fold in DMSO. A propidium iodide – SYBR® Green I (PISG) staining solution was additionally produced by supplementing this working SG solution with PI at a final concentration of 0.3 mM. A volume of 0.5 mL of final diluted sample was added to 5 µL of SG solution (final dilution of SG: 1:10,000), vortexed, and incubated at 37 °C for 10 min to promote dye penetration. This procedure was repeated for each sample using the PI-SG solution as the staining agent (final concentration of PI: 3 µM). After another vortexing step, SG and PI-SG stained samples were analyzed using a BD Accuri™ C6 flow cytometer (BD Accuri™ cytometers, Belgium). This instrument was equipped with a 20 mW solid-state laser emitting at a fixed wavelength of 488 nm. Green and red fluorescence intensities were respectively collected at 533 ± 30 nm (FL1) and > 670 nm (FL3). Sideward and forward scattered light intensities were measured as well. For each sample, a total volume of 50 µL was analyzed with fluidics set on fast and a lower threshold on the green fluorescence (FL1) set at 800.

S3.4.3 Parameter Derivation from Flow Cytometry

FC measurements were processed with the BD Accuri™ C6 analysis software. Sample analysis was performed without compensation. For each sample, recorded events were represented on a two-dimensional density plot of green fluorescence intensity against red fluorescence intensity. Intact (viable) and damaged (dead) cells were distinguished thanks to the differential penetration abilities of the two dyes: whereas SG (green fluorescence) penetrates both intact and damaged cells, PI (red fluorescence) only stains nucleic acids contained in destroyed membranes. Hence, FC measurements of SG and PISG stained samples respectively allowed to derive the concentration of total and viable cells:

$$C_{tot} = \frac{F_d * N_{SG}}{V_i} \tag{3}$$

$$C_{viable} = \frac{F_d * N_{PISG}}{V_i} \tag{4}$$

where F_d denotes the dilution factor (37,000), N_{SG} and N_{PISG} [cells] the count of events in the green fluorescence gate of SG and PISG stained samples, respectively, and V_i the injection volume (50 µL).

S3.4.4 Intact Cell Counts from Flow Cytometry

Tbl. S10: Measurements of intact cells concentration [cells nL^{-1}] before (-1 h) and after (72 h) the biotransformation assay as a mean of triplicates.

**Treatment**	**-1 [cells** nL^{-1}**]**	**72 [cells** nL^{-1}**]**
fresh_fresh_SN	601	783 ± 45
fresh_fresh_AW	563	851 ± 61
short_lyo_SN	179	739 ± 124
short_lyo_AW	204	918 ± 70
short_DMSO_SN	609	894 ± 134
short_DMSO_AW	662	1006 ± 109
short_gly_SN	712	932 ± 98
short_gly_AW	514	1032 ± 178
long_lyo_SN	231	731 ± 96
long_lyo_AE	203	890 ± 32
long_DMSO_SN	812	951 ± 79
long_DMSO_AE	890	1043 ± 47
long_gly_SN	751	1009 ± 136
long_gly_AE	854	1207 ± 120
fresh_sorption_SN	66	101 ± 89
fresh_sorption_AW	71	589 ± 953

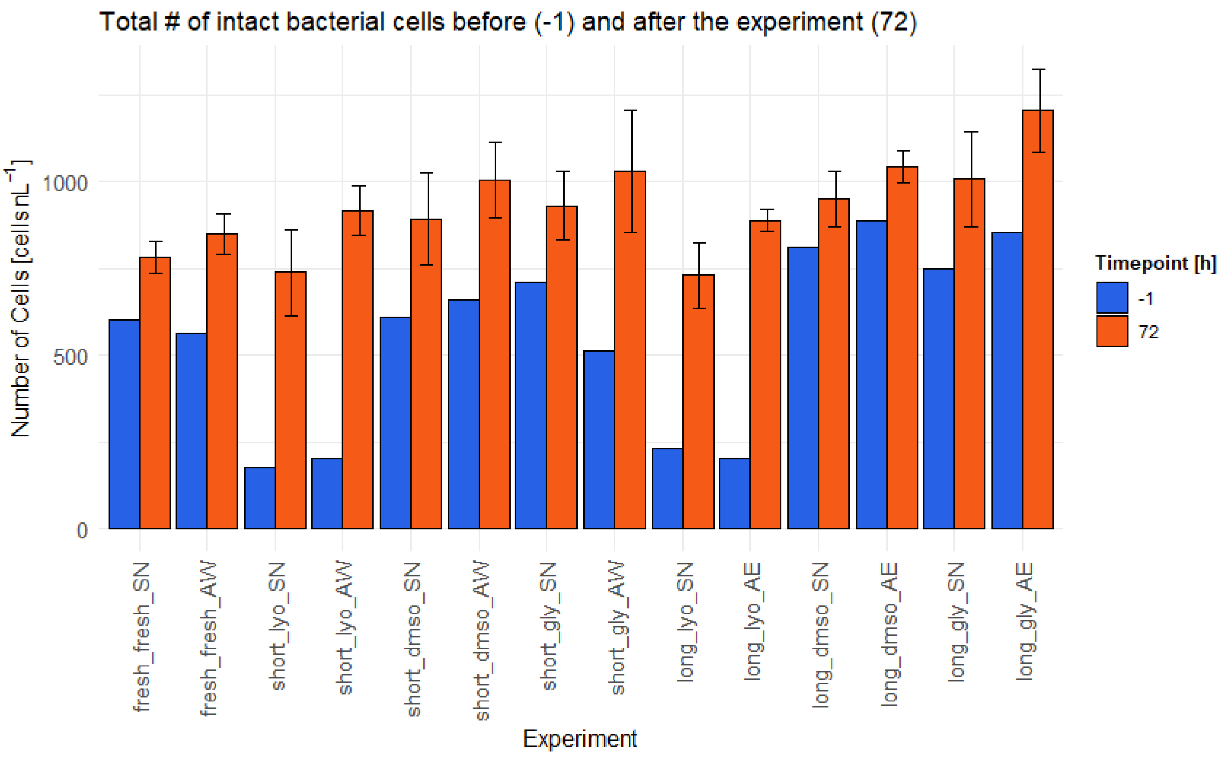

Fig. S4: Total growth of intact bacterial cells [cells nL] over the time span of the biotransformation experiment (72 h).

S3.5 Community Structure from 16S rRNA Amplicon Gene Sequencing

S3.5.1 Sampling for 16S rRNA Amplicon Gene Sequencing from Biotransformation Experiment

1 mL of homogenized AS at point in time −1 h and 72 h were taken from the batch reactors and centrifuged (21 130 rcf, 10 min). The supernatant was discarded and pellets were frozen at −20 °C until extraction.

S3.5.2 DNA Extraction

Genomic DNA was extracted using the DNeasy PowerBiofilm Kit (QIAGEN) according to manufacturer's instructions. The DNA quality and concentrations were checked using NanoDrop (Thermo Fisher Scientific) and Qubit 4 Fluorometer (Thermo Fisher Scientific)

before sending the samples for sequencing.

S3.5.3 Amplification, Library Preparation, and 16S rRNA Gene Amplicon Sequencing

The amplification, library preparation and sequencing using the Illumina NovaSeq 6000 (PE250) platform was performed by Novogene. Briefly, the 16S V3-V4 regions were amplified using the primers 341F (CCTAYGGGRBGCASCAG) and 806R (GGACTACNNGGGTATCTAAT) with the barcode. All PCR reactions were carried out with 15 µL of Phusion High-Fidelity PCR Master Mix (New England Biolabs), 2 µM of forward and reverse primers, and 10 ng template DNA. Thermal cycling consisted of initial denaturation at 98 °C for 1 min, followed by 30 cycles of denaturation at 98 °C for 10 s, annealing at 50 °C for 30 s, elongation at 72 °C for 30 s, and finally 72 °C for 5 min. Sequencing libraries were generated using TruSeq DNA PCR-Free Sample Preparation Kit (Illumina, USA) following the manufacturer's recommendations, and index codes were added. The library quality was assessed using the Qubit 2.0 Fluorometer (Thermo Scientific) and Agilent Bioanalyzer 2100 system before sequencing.

S3.5.4 16S rRNA Gene Sequencing Analysis

Data analysis was performed on the Easy Microbiome Analysis Platform (EasyMAP).[17] This included demultiplexing, quality control, taxonomy assignment, microbial composition, heatmap generation and determination of alpha and beta diversity . After screening, the sequences were trimmed at 400 bp and a quality score $\geq$25 was ensured. The sequences were then filtered using DADA2,[18,19] and later a Greengenes-V34 classifier reference[20] was used with a 70 % confidence threshold for taxonomic analysis.[21,22] To assess alpha diversity, the observed species richness[23] as well as the Shannon diversity index were calculated.[24] After normalizing the data, pairwise t-tests[25] were performed between the post-biotransformation samples to detect statistically significant differences in alpha diversity. principal coordinate

analysis (PCoA) was performed using the weighted UniFrac distances and the scikit-bio python package[26] for beta diversity analysis. The sequencing raw reads were deposited to the National Center for Biotechnology Information (NCBI) SRA database under accession No. PRJNA1132939.

S3.5.5 Alpha Diversity of the Samples

Alpha diversity of the samples was assessed using sample richness, represented by the number of observed species per sample, as well as diversity and evenness, represented by the Shannon diversity index (H'). For the number of observed OTUs per sample, the lowest observed value was 400 OTUs in the long_lyo_SN_72 samples, and the highest was 956 in the short_gly_SN_72 samples. In the native AS sample, 792 species were identified. The number of observed species for CRYO samples was in the range of 750 to 950, whereas for LYO samples, the number ranged from 400 to 750. The results of the pairwise t-tests determining the significance of the diversity shift throughout the biotransformation experiment by comparing the −1 h sample with the (72 h) sample showed significant differences only for the samples subjected to LYO treatment (Tbl. S12 and Tbl. S11).

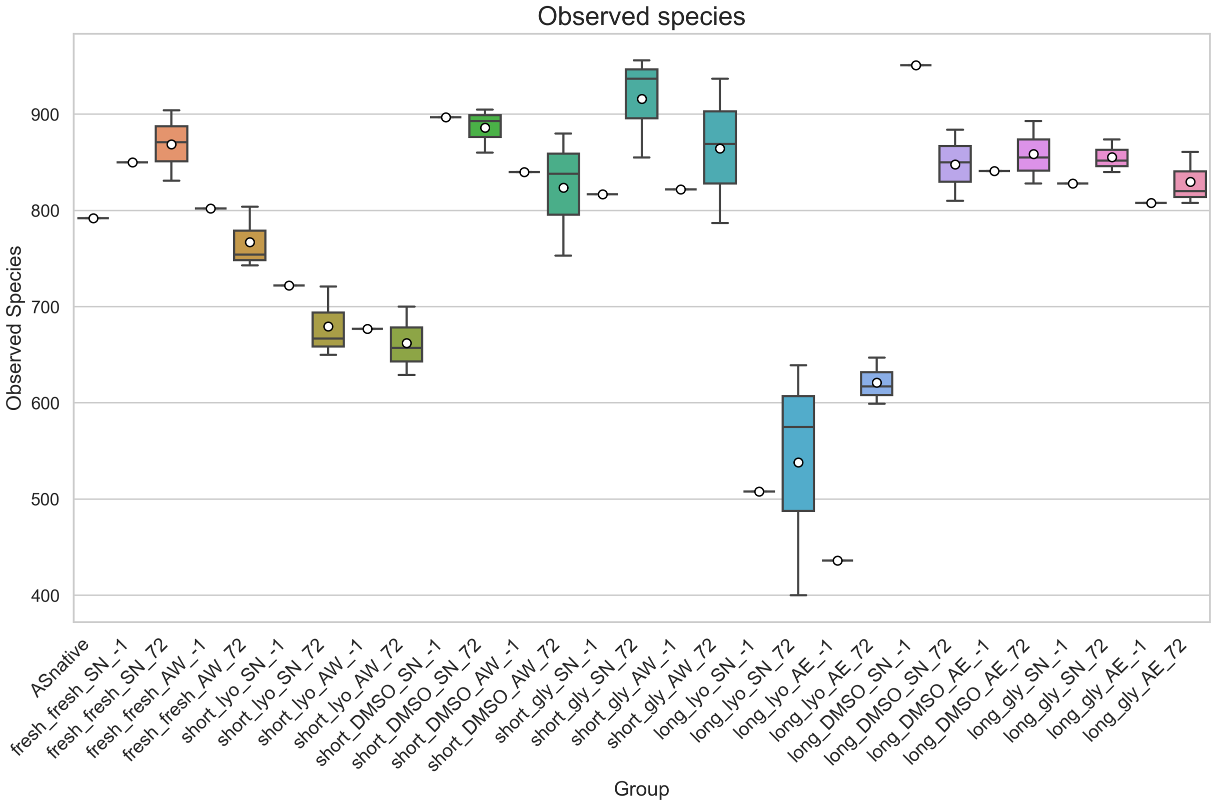

Fig. S5: OTUs, point in time −1 h as unique, point in time 72 h as triplicates.

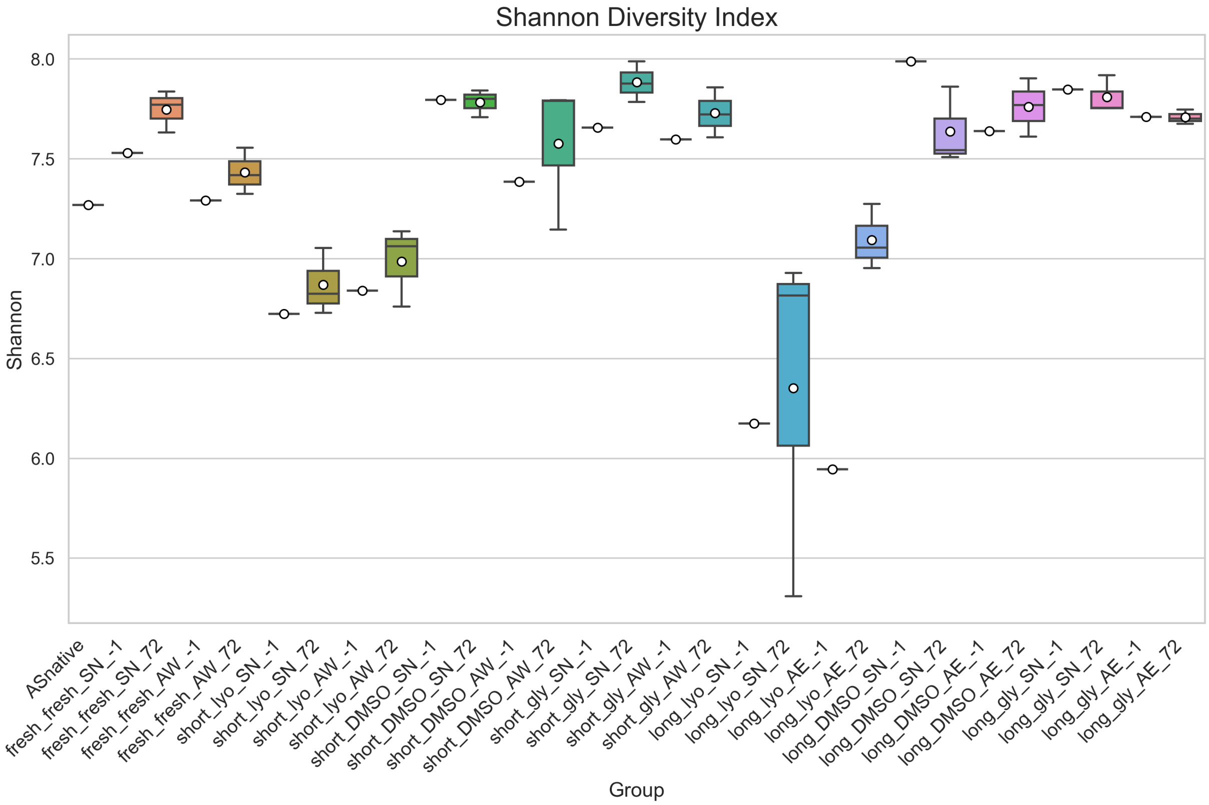

Fig. S6: Shannon diversity, point in time -1 h as unique, point in time 72 h as triplicates.

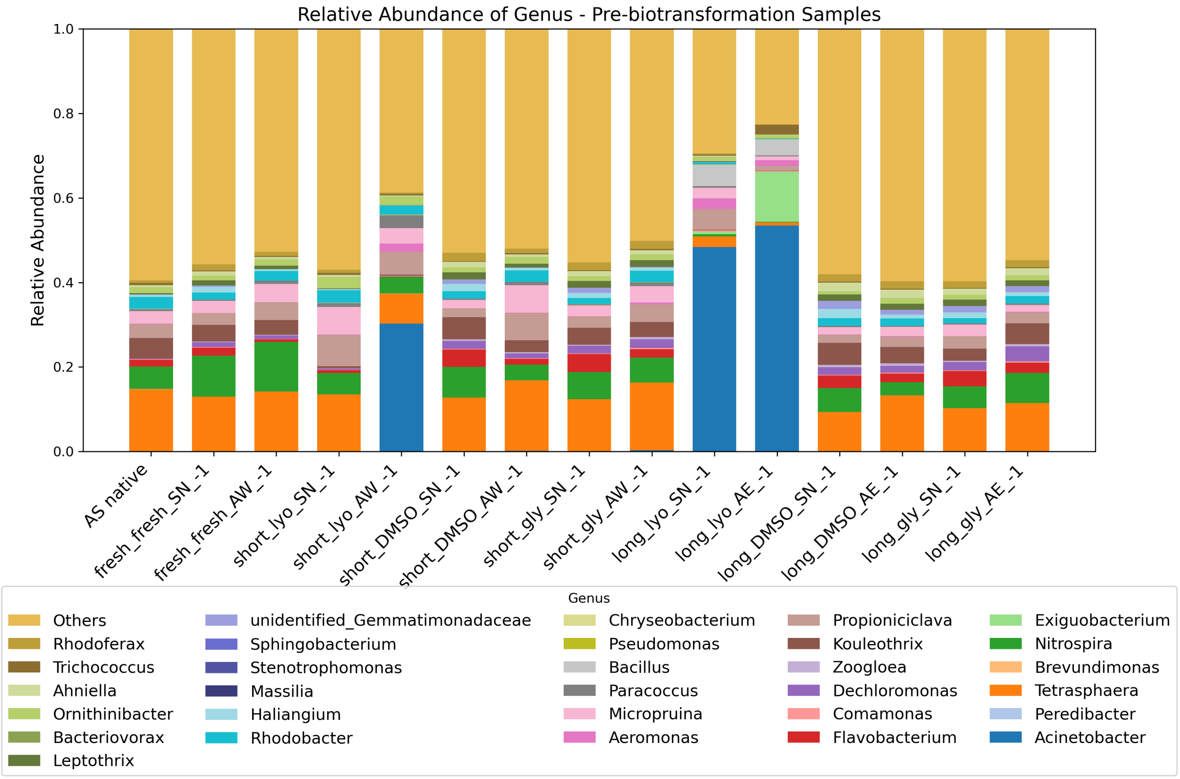

Fig. S7: Genus diversity for native fresh AS and for all treatment samples at time-Point -1 h.

Tbl. S11: Results of the pairwise t-tests on the operational taxonomic units (OTUs) for the samples of time-point 72 h.

Groups	long_DMSO_AE_72	short_DMSO_AW_72	long_DMSO_SN_72	short_DMSO_SN_72	fresh_fresh_AW_72	fresh_fresh_SN_72	long_gly_AE_72	short_gly_AW_72	long_gly_SN_72	short_gly_SN_72	long_lyo_AE_72	short_lyo_AW_72	long_lyo_SN_72
**short_DMSO_AW_72**	1												
**long_DMSO_SN_72**	1	1											
**short_DMSO_SN_72**	1	1	1										
**fresh_fresh_AW_72**	1	1	1	0.509611									
**fresh_fresh_SN_72**	1	1	1	1	1								
**long_gly_AE_72**	1	1	1	1	1	1							
**short_gly_AW_72**	1	1	1	1	1	1	1						
**long_gly_SN_72**	1	1	1	1	1	1	1	1					
**short_gly_SN_72**	1	1	1	1	0.078066	1	1	1	1				
**long_lyo_AE_72**	0.00038	0.003459	0.000771	8.46E-05	0.095938	0.0002	0.002456	0.000302	0.000474	1.11E-05			
**short_lyo_AW_72**	0.004222	0.03686	0.008522	0.000942	0.868502	0.002286	0.025721	0.003363	0.005263	0.000125	1		
**long_lyo_SN_72**	2.87E-06	2.46E-05	5.64E-06	6.98E-07	0.000765	1.56E-06	1.72E-05	2.29E-06	3.53E-06	1.07E-07	1	0.4222	
**short_lyo_SN_72**	0.011853	0.10008	0.024052	0.002753	1	0.006283	0.073227	0.009331	0.014728	0.000361	1	1	0.156029

Tbl. S12: Results of the pairwise t-tests on the shannon diversity index for the samples of time-point 72 h.

Groups	long_DMSO_AE_72	short_DMSO_AW_72	long_DMSO_SN_72	short_DMSO_SN_72	fresh_fresh_AW_72	fresh_fresh_SN_72	long_gly_AE_72	short_gly_AW_72	long_gly_SN_72	short_gly_SN_72	long_lyo_AE_72	short_lyo_AW_72	long_lyo_SN_72
**short_DMSO_AW_72**	1												
**long_DMSO_SN_72**	1	1											
**short_DMSO_SN_72**	1	1	1										
**fresh_fresh_AW_72**	0.76867	1	1	0.54051									
**fresh_fresh_SN_72**	1	1	1	1	1								
**long_gly_AE_72**	1	1	1	1	1	1							
**short_gly_AW_72**	1	1	1	1	1	1	1						
**long_gly_SN_72**	1	1	1	1	0.30206	1	1	1					
**short_gly_SN_72**	1	1	1	1	0.04859	1	1	1	1				
**long_lyo_AE_72**	0.00821	0.19259	0.10318	0.00543	1	0.01218	0.03134	0.01743	0.00282	0.00043			
**short_lyo_AW_72**	0.00251	0.05985	0.03241	0.00165	1	0.00374	0.00964	0.00538	0.00087	0.00013	1		
**long_lyo_SN_72**	0.000054	0.00143	0.00075	3.60E-05	0.03903	8.20E-05	0.00021	0.00012	1.90E-05	2.80E-06	1	1	
**short_lyo_SN_72**	0.00074	0.01802	0.00964	0.00049	0.4556	0.0011	0.00282	0.00157	0.00025	3.70E-05	1	1	1

S4 Test Substance Concentration-Time Series

Linearized concentration-time series per treatment (fresh: yellow, LYO: purple, GLY: blue, and DMSO: green) averaged over the experimental triplicates, separated in columns for the three experiment (fresh, short, and long) and into rows for the use of native media (SN, top) and artificial media (AW or AE, bottom). Time-point linking line is depicted in red, if the derived rate constant does not fulfill the quality requirements (non-valid is TRUE), else black.

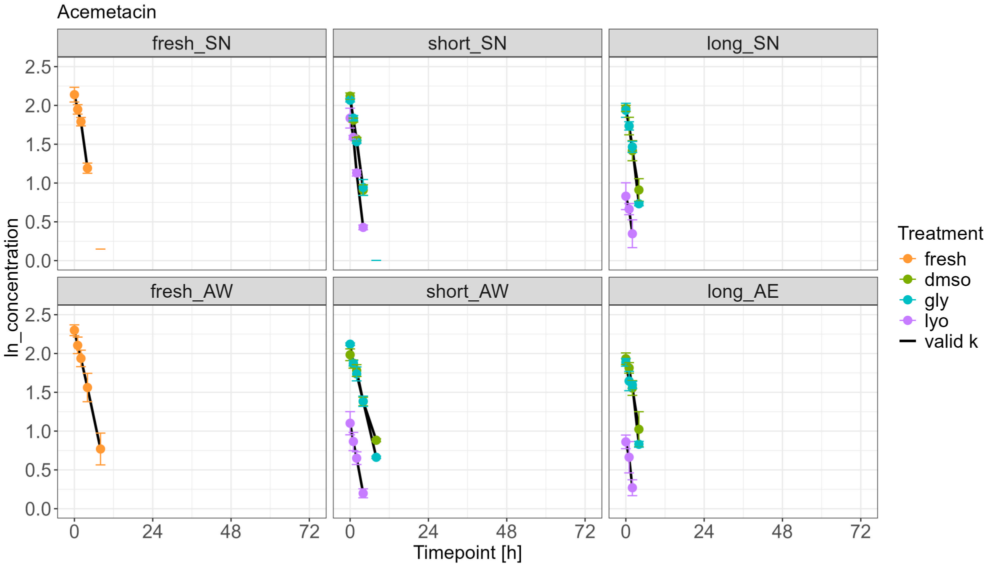

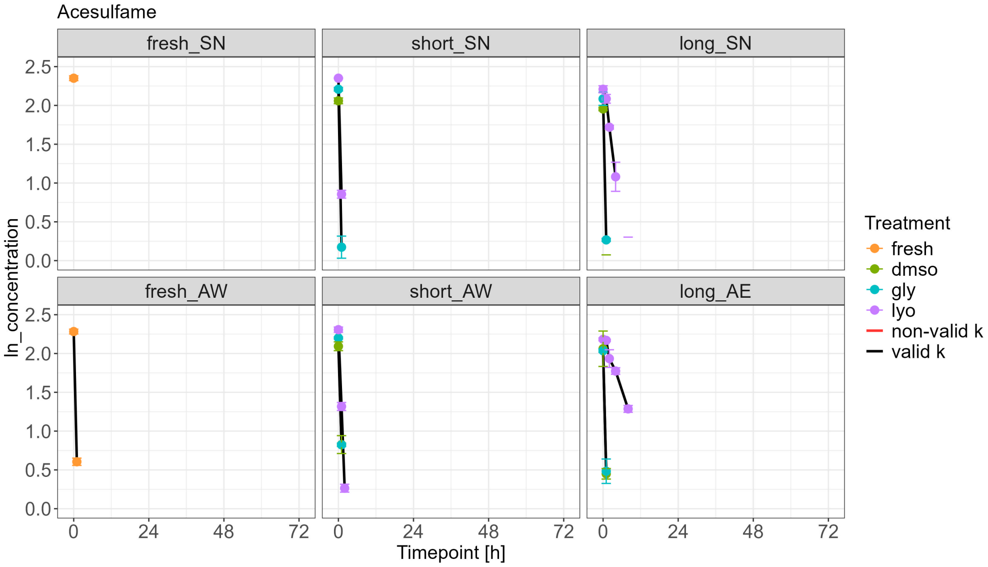

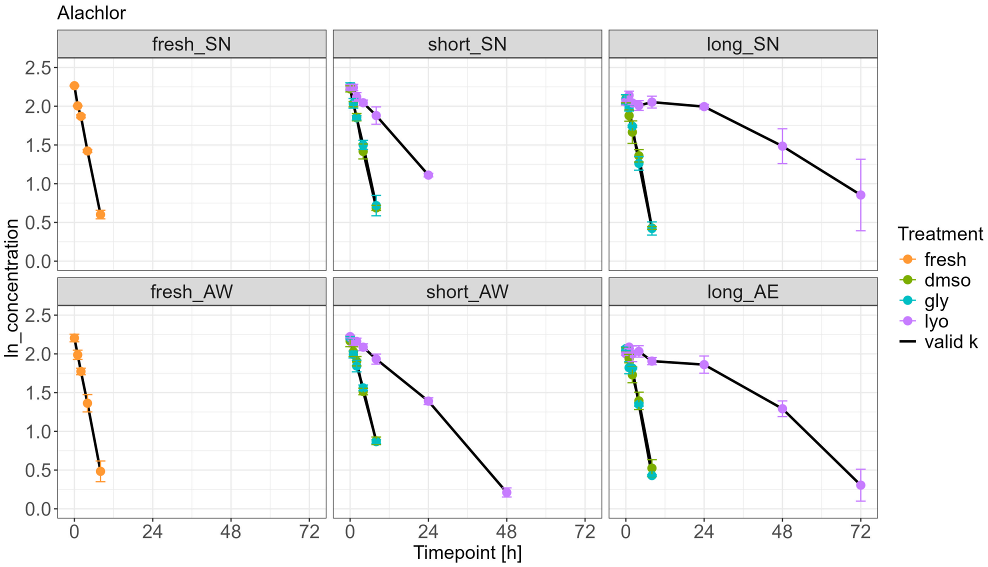
Alachlor
fresh_SN
short_SN
long_SN
fresh_AW
short_AW
long_AE
ln_concentration
Timepoint [h]
Treatment
fresh
dmso
gly
lyo
valid k

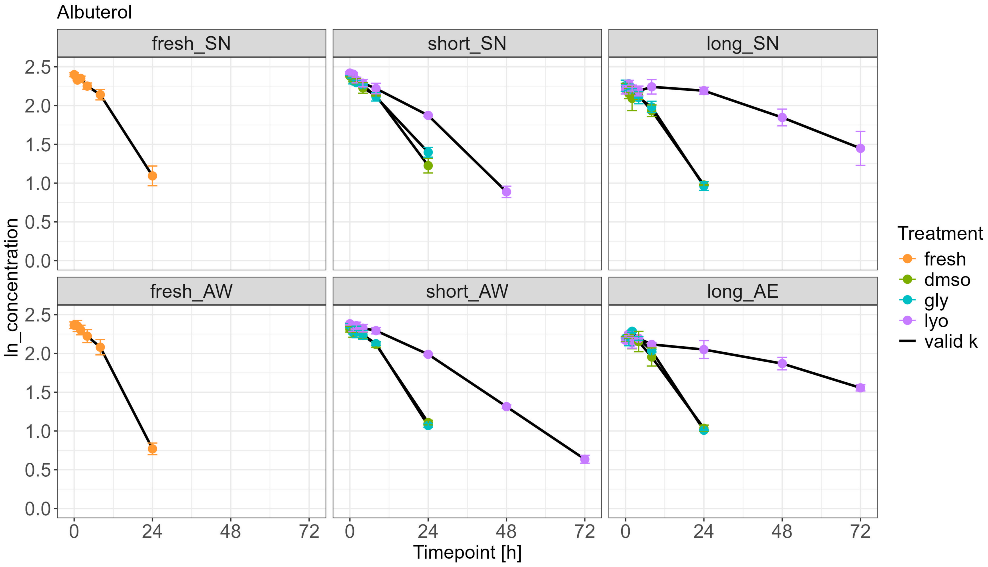
Albuterol
fresh_SN
short_SN
long_SN
fresh_AW
short_AW
long_AE
ln_concentration
Timepoint [h]
Treatment
fresh
dmso
gly
lyo
valid k

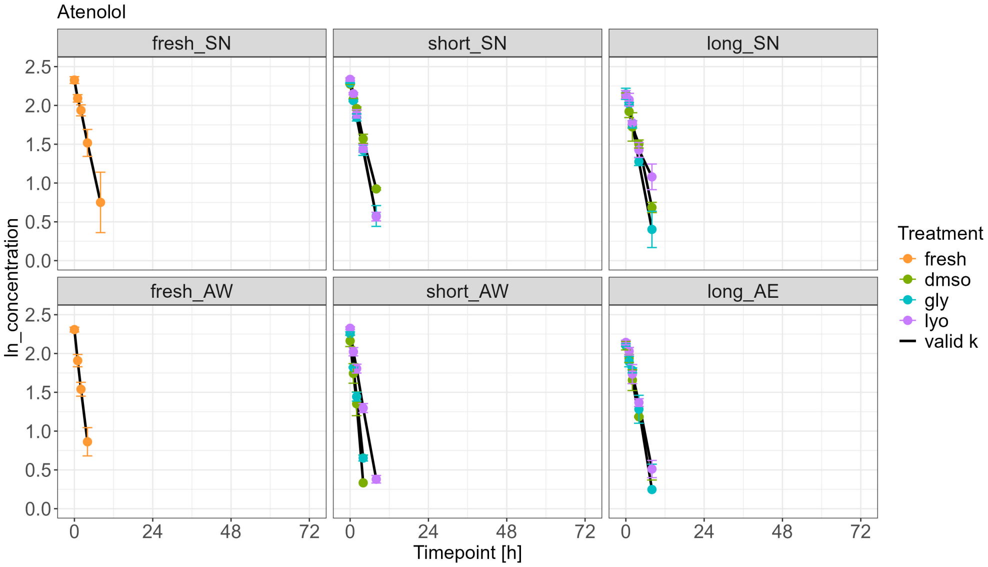
Atenolol
fresh_SN
short_SN
long_SN
fresh_AW
short_AW
long_AE
ln_concentration
Timepoint [h]
Treatment
fresh
dmso
gly
lyo
valid k

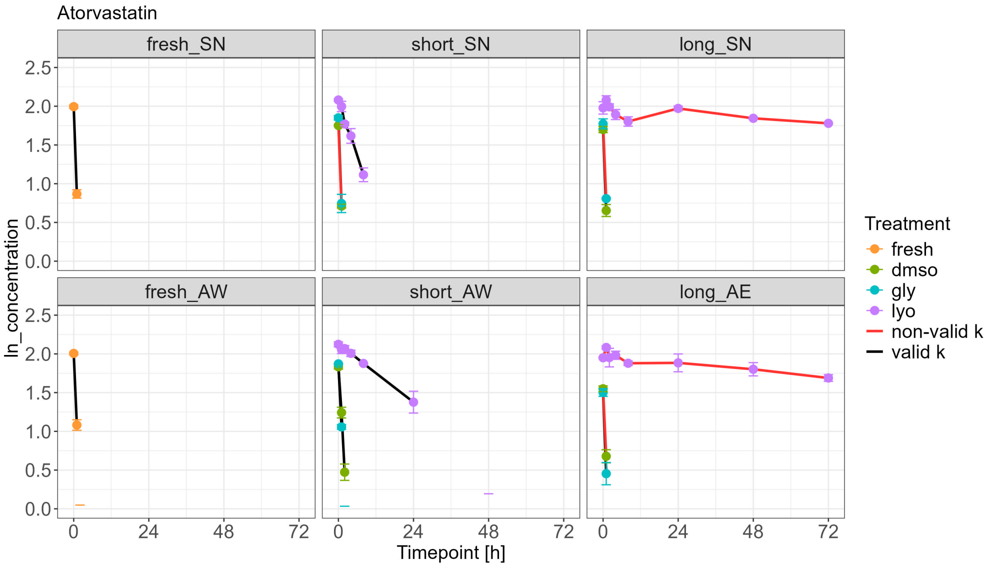
Atorvastatin
fresh_SN
short_SN
long_SN
fresh_AW
short_AW
long_AE
ln_concentration
Timepoint [h]
0.0
0.5
1.0
1.5
2.0
2.5
0
24
48
72
Treatment
fresh
dmso
gly
lyo
non-valid k
valid k

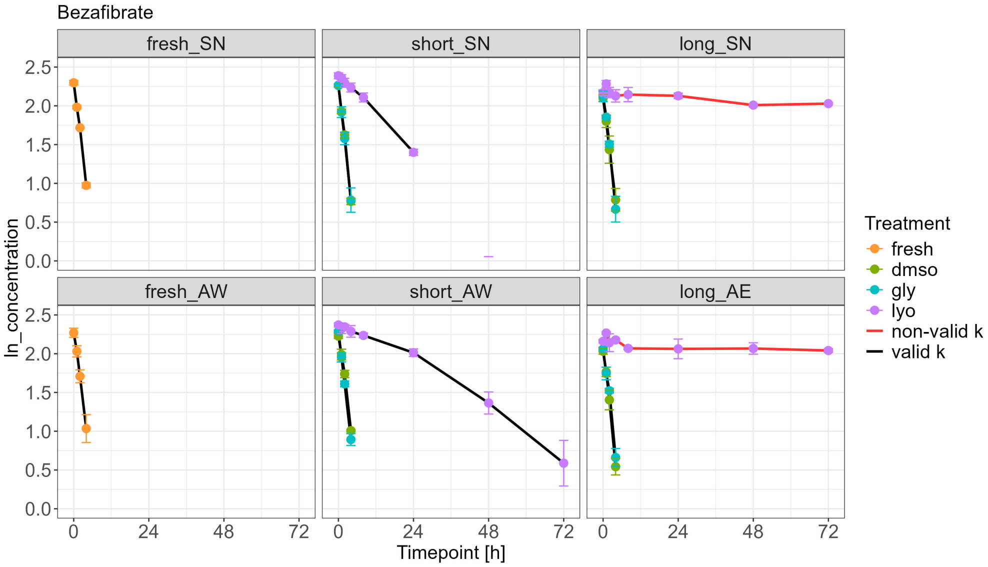
Bezafibrate
fresh_SN
short_SN
long_SN
fresh_AW
short_AW
long_AE
ln_concentration
Timepoint [h]
0.0
0.5
1.0
1.5
2.0
2.5
0
24
48
72
Treatment
fresh
dmso
gly
lyo
non-valid k
valid k

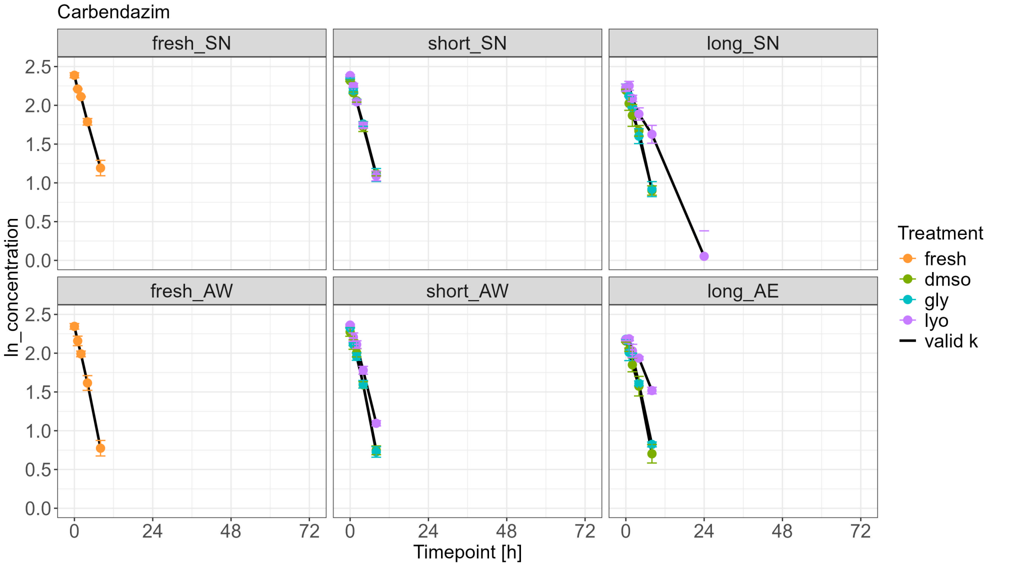
Carbendazim
fresh_SN
short_SN
long_SN
fresh_AW
short_AW
long_AE
ln_concentration
Timepoint [h]
0.0
0.5
1.0
1.5
2.0
2.5
0
24
48
72
Treatment
fresh
dmso
gly
lyo
valid k

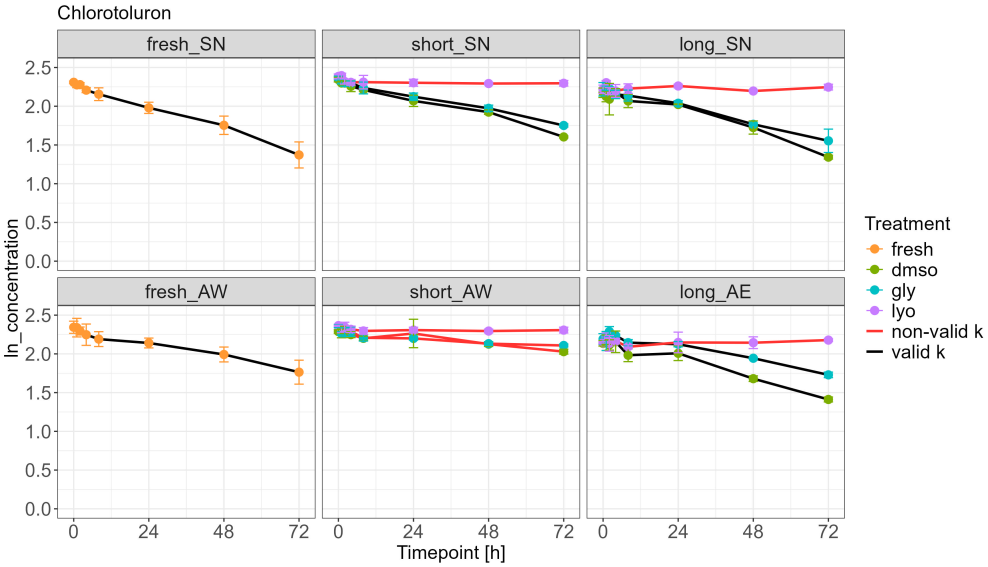
Chlorotoluron
fresh_SN
short_SN
long_SN
fresh_AW
short_AW
long_AE
ln_concentration
Timepoint [h]
0.0
0.5
1.0
1.5
2.0
2.5
0
24
48
72
Treatment
fresh
dmso
gly
lyo
non-valid k
valid k

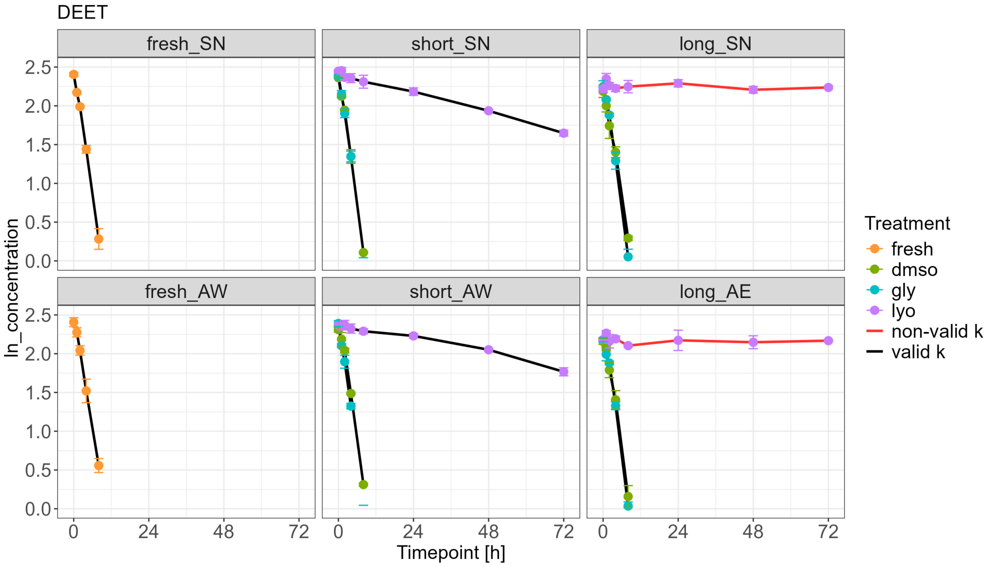
DEET
fresh_SN
short_SN
long_SN
fresh_AW
short_AW
long_AE
ln_concentration
Timepoint [h]
0.0
0.5
1.0
1.5
2.0
2.5
0
24
48
72
Treatment
fresh
dmso
gly
lyo
non-valid k
valid k

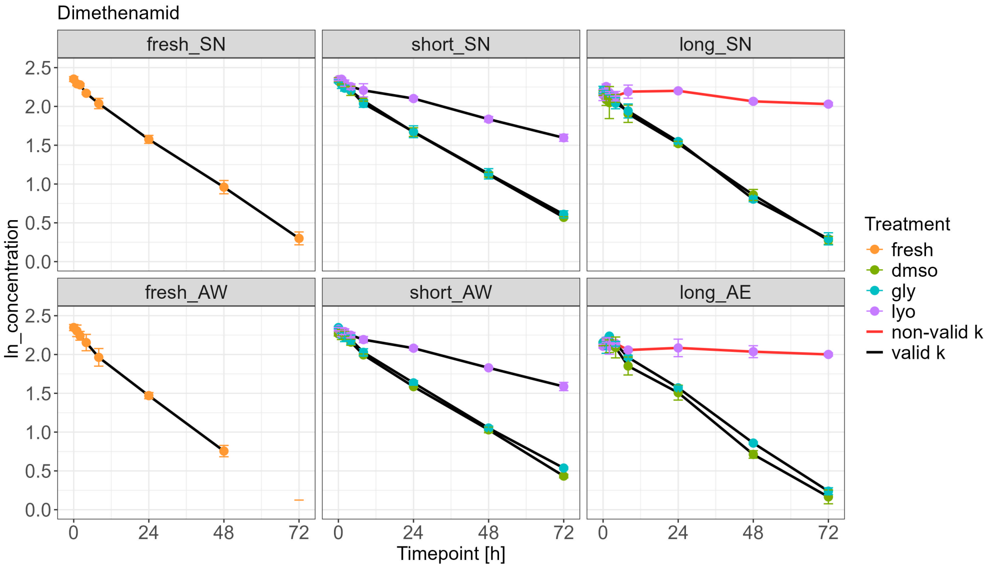
Dimethenamid
fresh_SN
short_SN
long_SN
fresh_AW
short_AW
long_AE
ln_concentration
Timepoint [h]
0.0
0.5
1.0
1.5
2.0
2.5
0
24
48
72
Treatment
fresh
dmso
gly
lyo
non-valid k
valid k

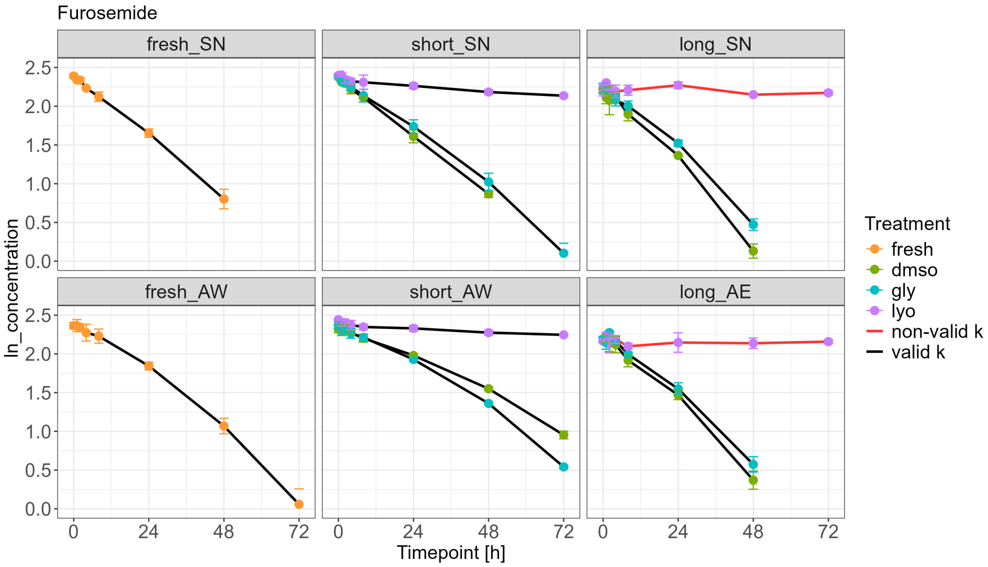
Furosemide
fresh_SN
short_SN
long_SN
fresh_AW
short_AW
long_AE
ln_concentration
Timepoint [h]
Treatment
fresh
dmso
gly
lyo
non-valid k
valid k

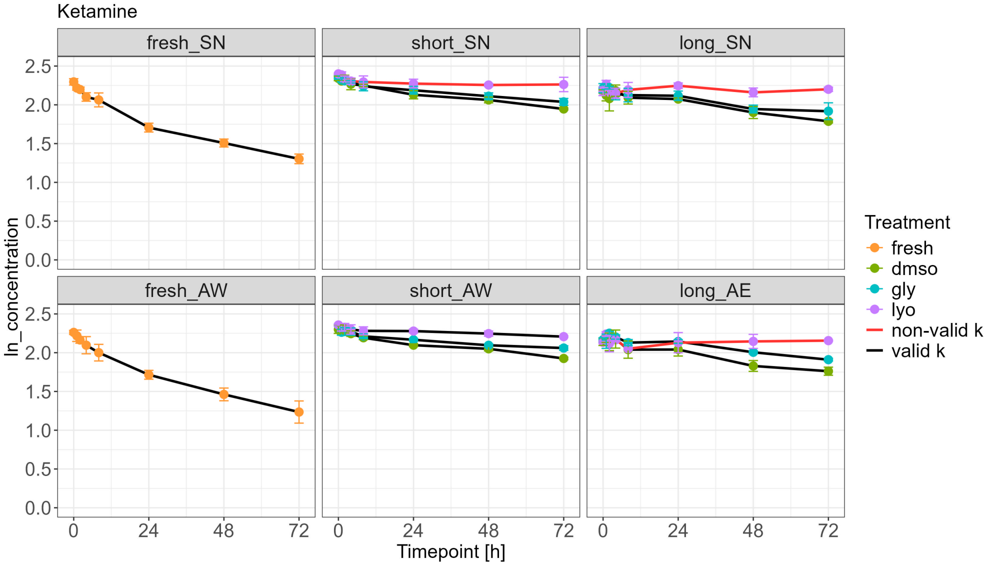
Ketamine
fresh_SN
short_SN
long_SN
fresh_AW
short_AW
long_AE
ln_concentration
Timepoint [h]
Treatment
fresh
dmso
gly
lyo
non-valid k
valid k

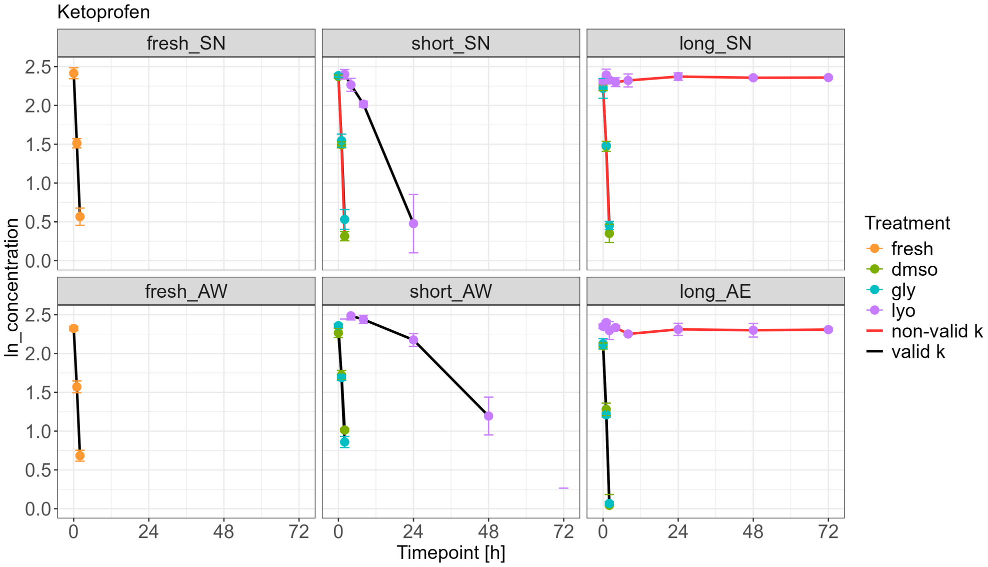
Ketoprofen
fresh_SN
short_SN
long_SN
fresh_AW
short_AW
long_AE
ln_concentration
Timepoint [h]
Treatment
fresh
dmso
gly
lyo
non-valid k
valid k

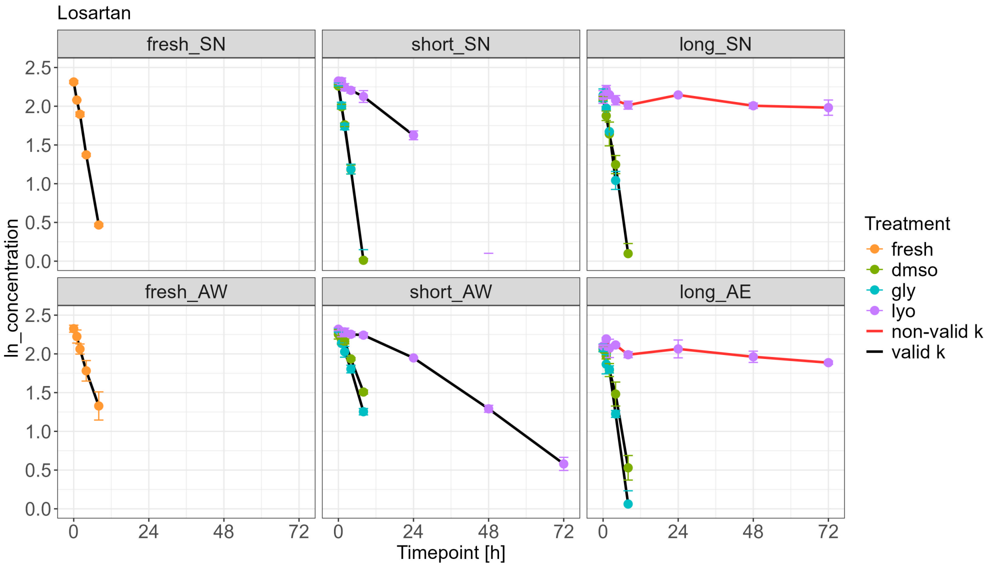
Losartan
fresh_SN
short_SN
long_SN
fresh_AW
short_AW
long_AE
ln_concentration
Timepoint [h]
Treatment
fresh
dmso
gly
lyo
non-valid k
valid k

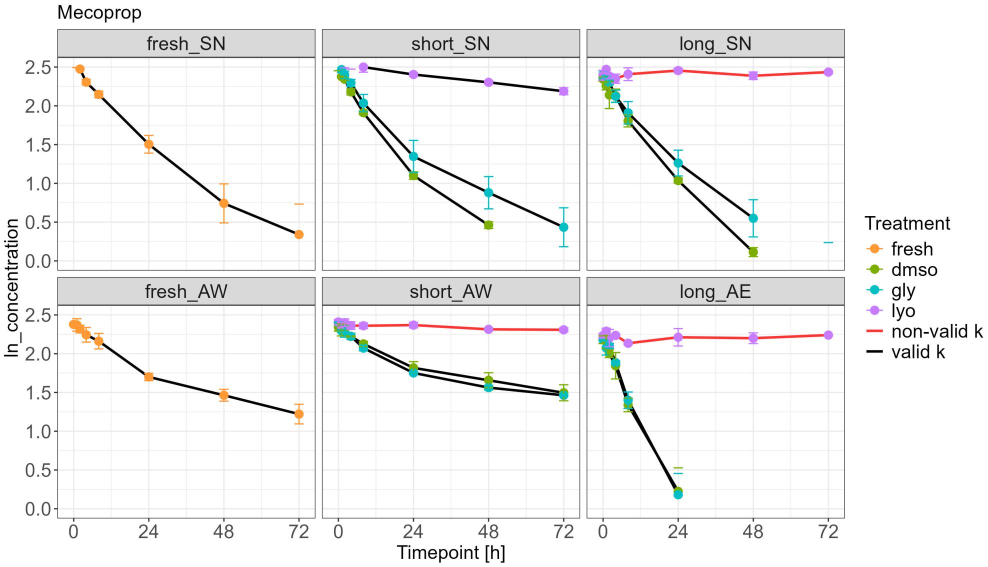
Mecoprop
fresh_SN
short_SN
long_SN
fresh_AW
short_AW
long_AE
ln_concentration
Timepoint [h]
Treatment
fresh
dmso
gly
lyo
non-valid k
valid k

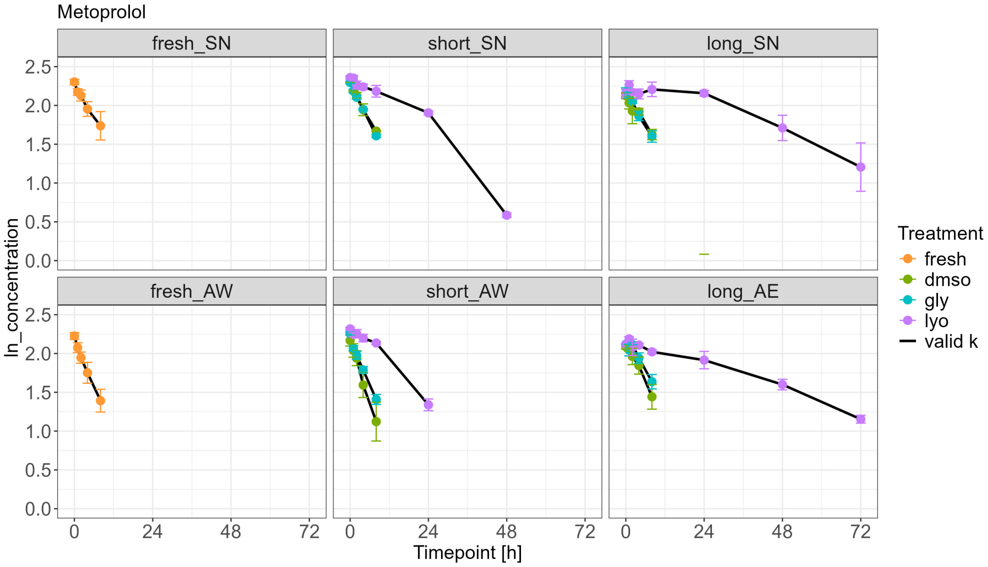
Metoprolol
fresh_SN
short_SN
long_SN
fresh_AW
short_AW
long_AE
ln_concentration
Timepoint [h]
Treatment
fresh
dmso
gly
lyo
valid k

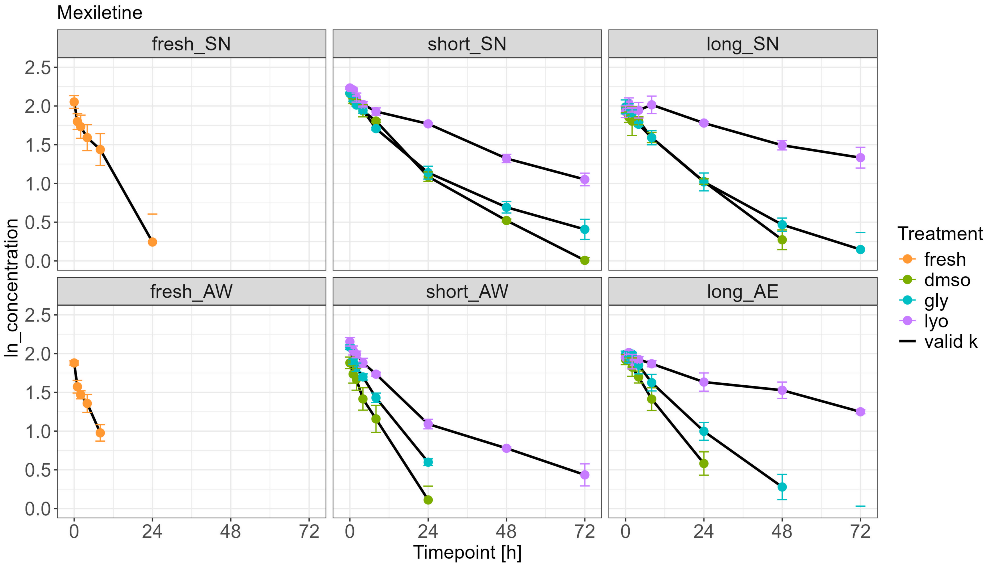

Mexiletine
fresh_SN
short_SN
long_SN
fresh_AW
short_AW
long_AE
ln_concentration
Timepoint [h]
Treatment
fresh
dmso
gly
lyo
valid k

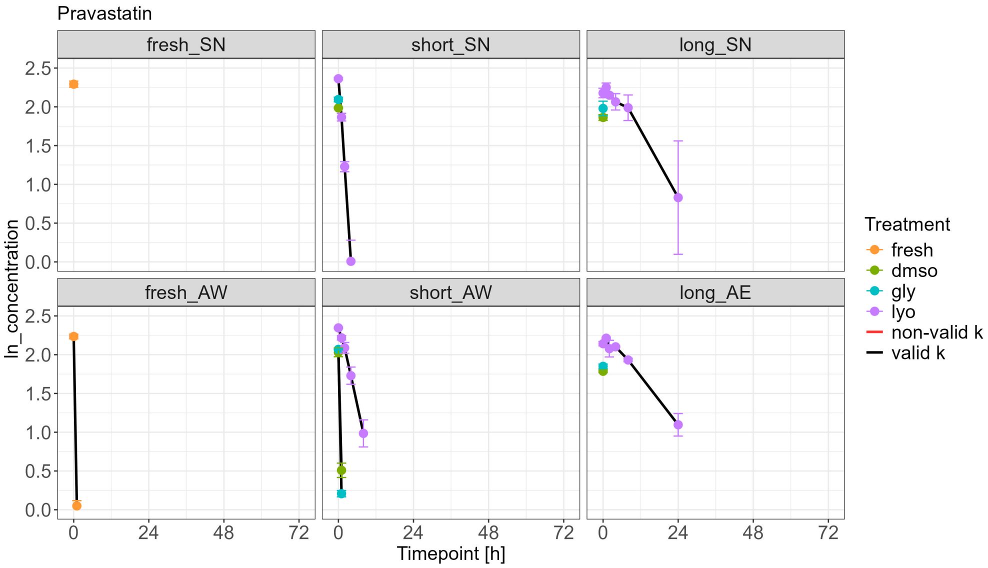

Pravastatin
fresh_SN
short_SN
long_SN
fresh_AW
short_AW
long_AE
ln_concentration
Timepoint [h]
Treatment
fresh
dmso
gly
lyo
non-valid k
valid k

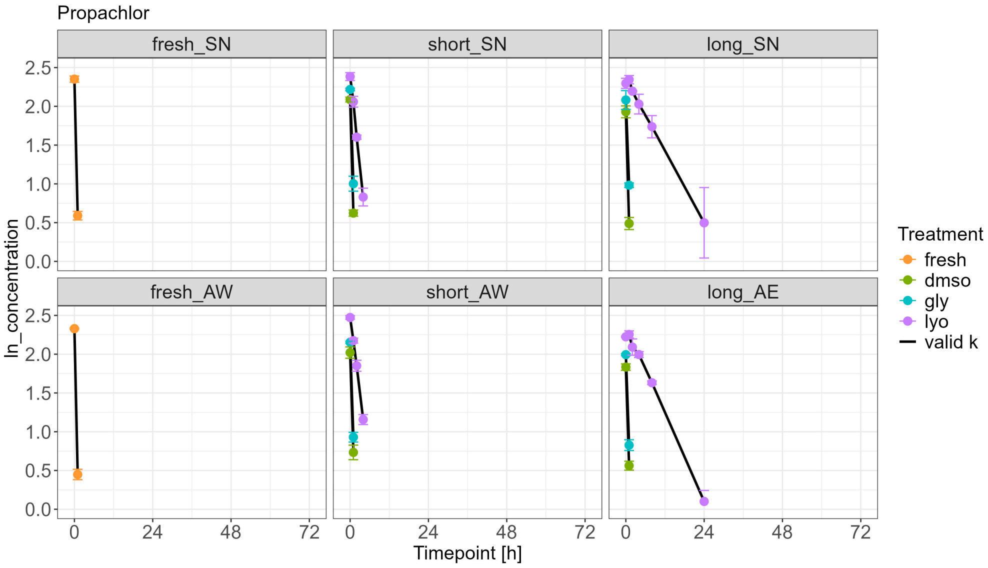

Propachlor
fresh_SN
short_SN
long_SN
fresh_AW
short_AW
long_AE
ln_concentration
Timepoint [h]
Treatment
fresh
dmso
gly
lyo
valid k

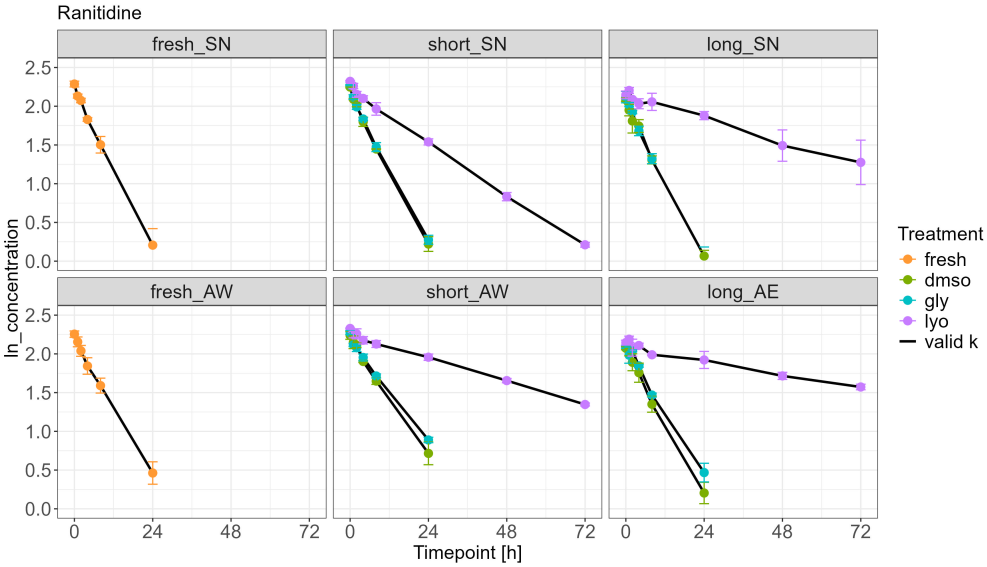
Ranitidine
fresh_SN
short_SN
long_SN
fresh_AW
short_AW
long_AE
ln_concentration
Timepoint [h]
Treatment
fresh
dmso
gly
lyo
valid k

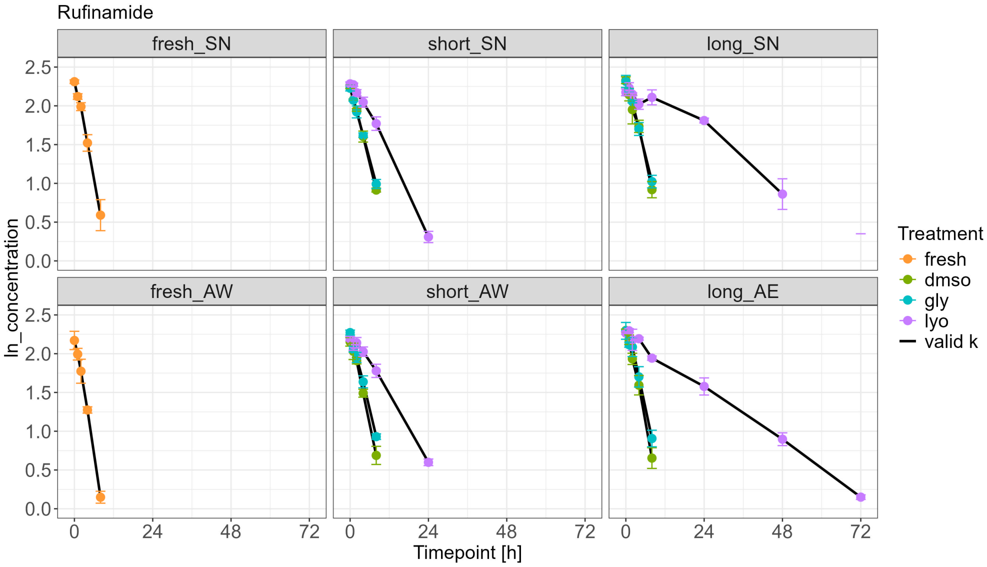
Rufinamide
fresh_SN
short_SN
long_SN
fresh_AW
short_AW
long_AE
ln_concentration
Timepoint [h]
Treatment
fresh
dmso
gly
lyo
valid k

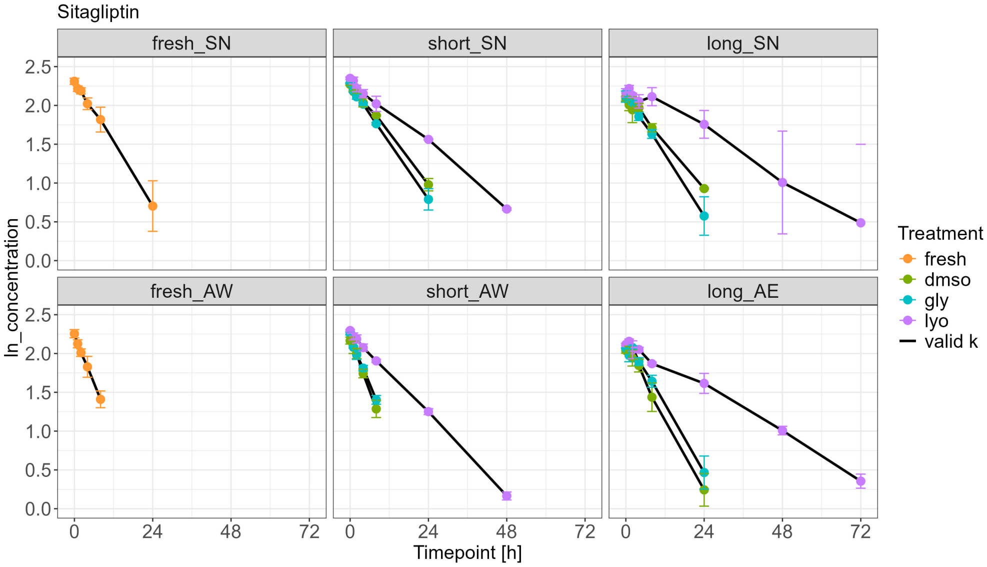
Sitagliptin
fresh_SN
short_SN
long_SN
fresh_AW
short_AW
long_AE
ln_concentration
Timepoint [h]
Treatment
fresh
dmso
gly
lyo
valid k

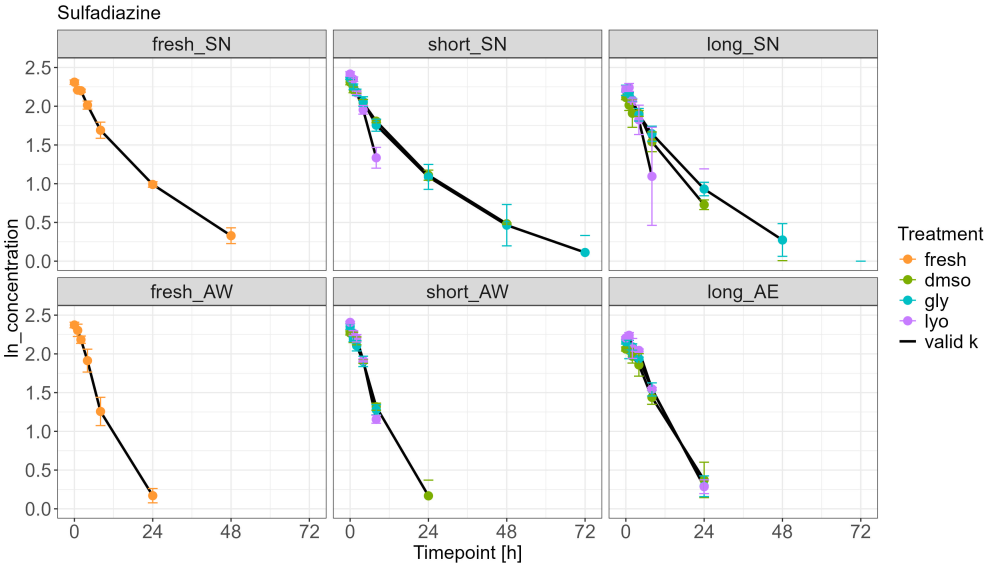

Sulfadiazine
fresh_SN
short_SN
long_SN
fresh_AW
short_AW
long_AE
ln_concentration
Timepoint [h]
Treatment
fresh
dmso
gly
lyo
valid k

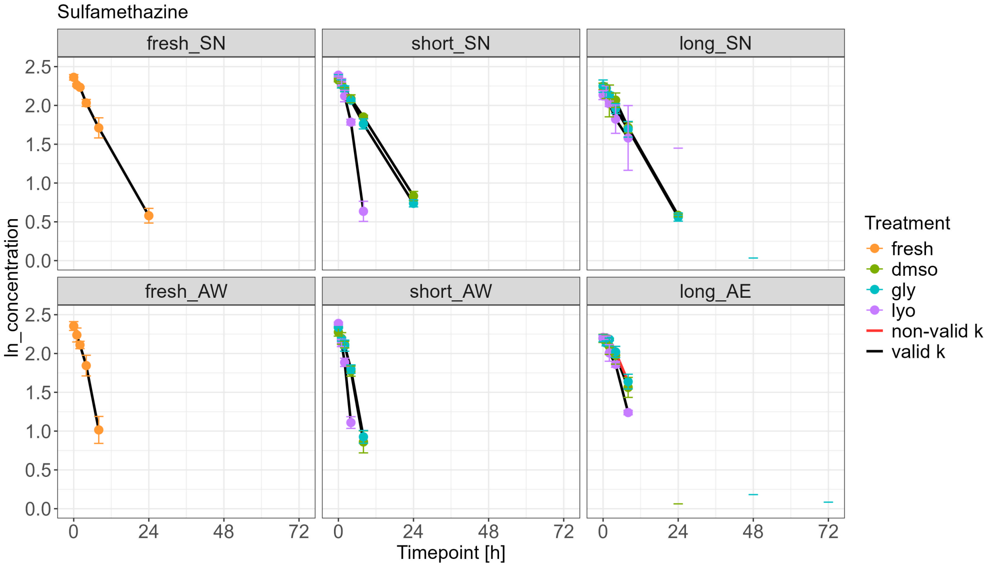

Sulfamethazine
fresh_SN
short_SN
long_SN
fresh_AW
short_AW
long_AE
ln_concentration
Timepoint [h]
Treatment
fresh
dmso
gly
lyo
non-valid k
valid k

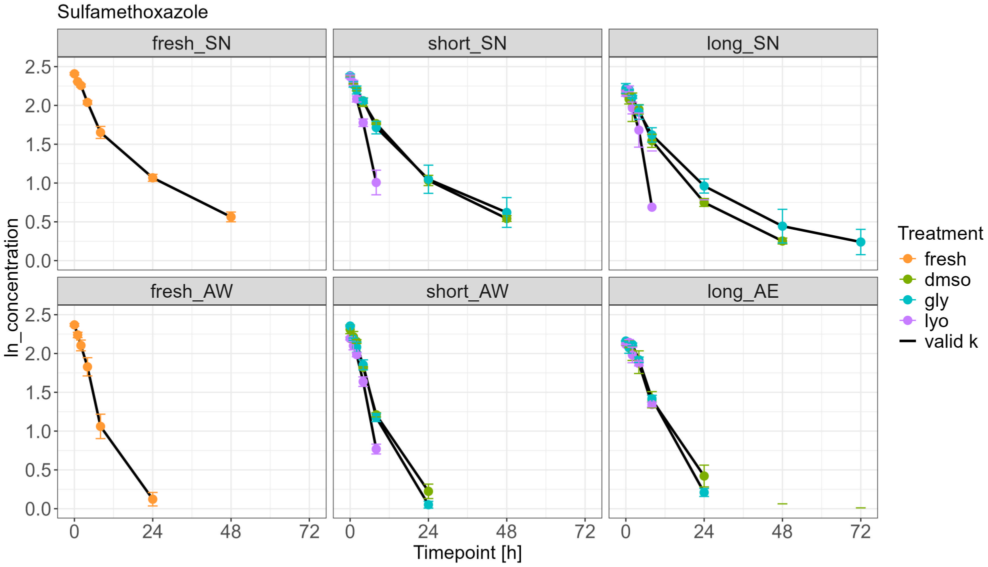

Sulfamethoxazole
fresh_SN
short_SN
long_SN
fresh_AW
short_AW
long_AE
ln_concentration
Timepoint [h]
Treatment
fresh
dmso
gly
lyo
valid k

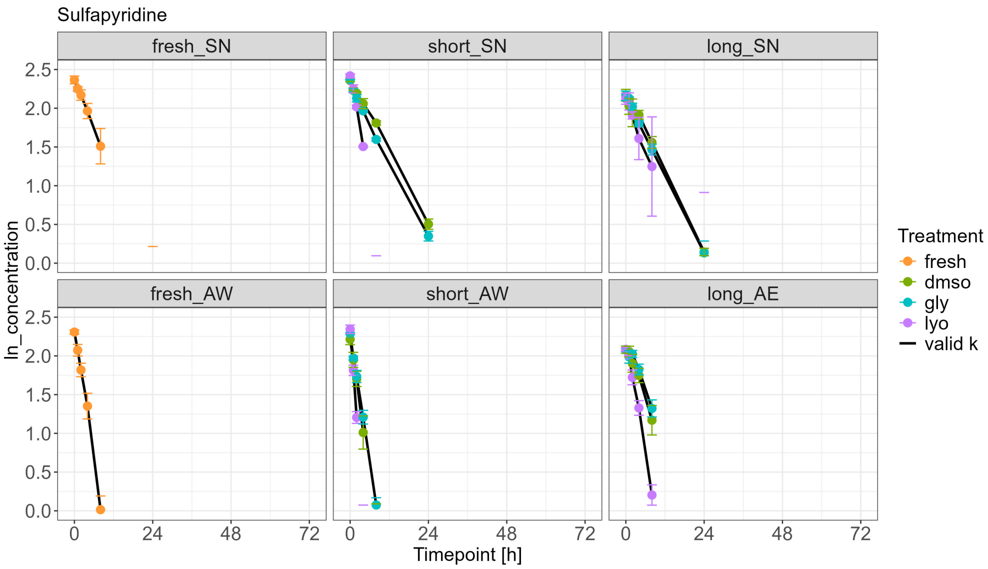
Sulfapyridine
fresh_SN
short_SN
long_SN
fresh_AW
short_AW
long_AE
ln_concentration
Timepoint [h]
Treatment
fresh
dmso
gly
lyo
valid k

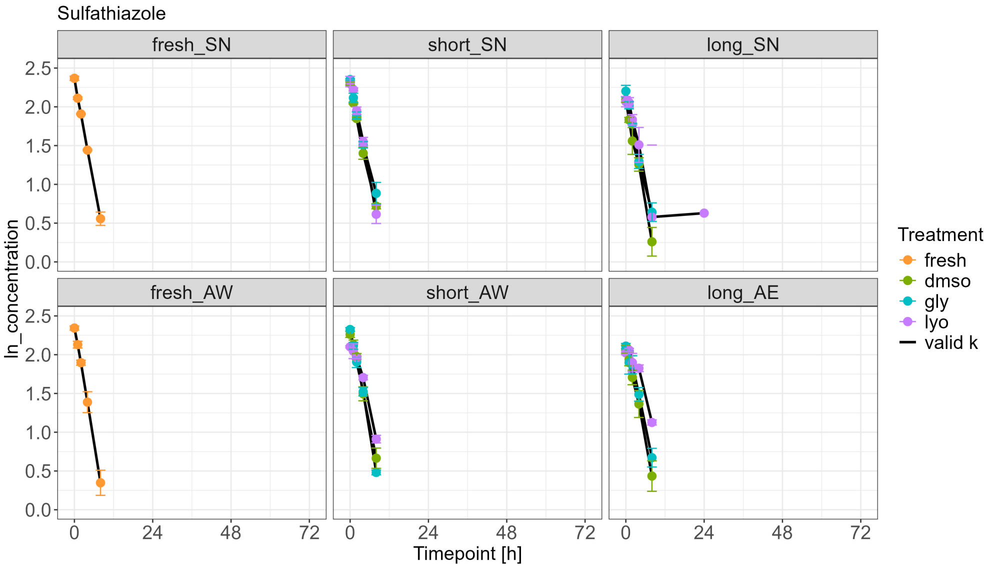
Sulfathiazole
fresh_SN
short_SN
long_SN
fresh_AW
short_AW
long_AE
ln_concentration
Timepoint [h]
Treatment
fresh
dmso
gly
lyo
valid k

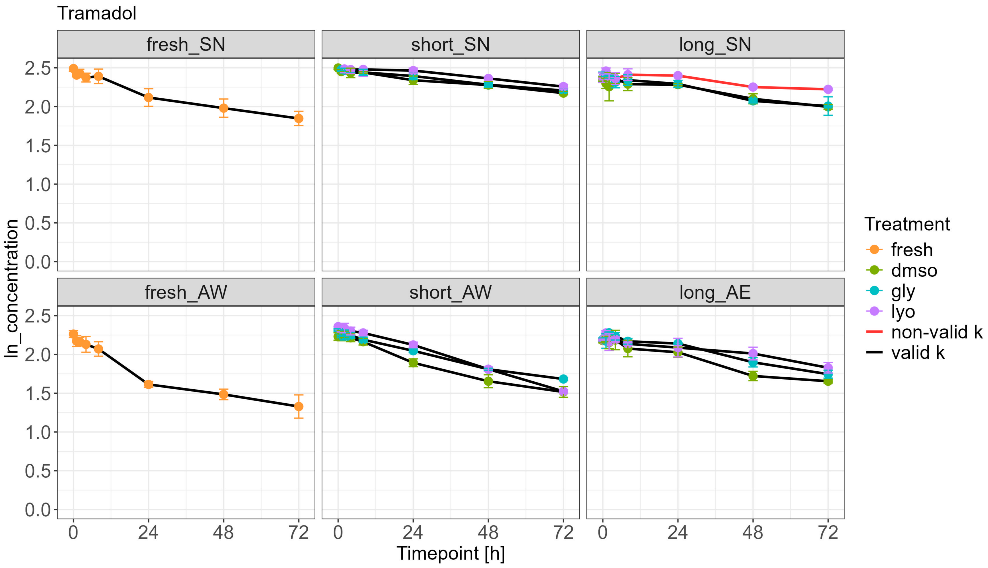
Tramadol
fresh_SN
short_SN
long_SN
fresh_AW
short_AW
long_AE
ln_concentration
Timepoint [h]
Treatment
fresh
dmso
gly
lyo
non-valid k
valid k

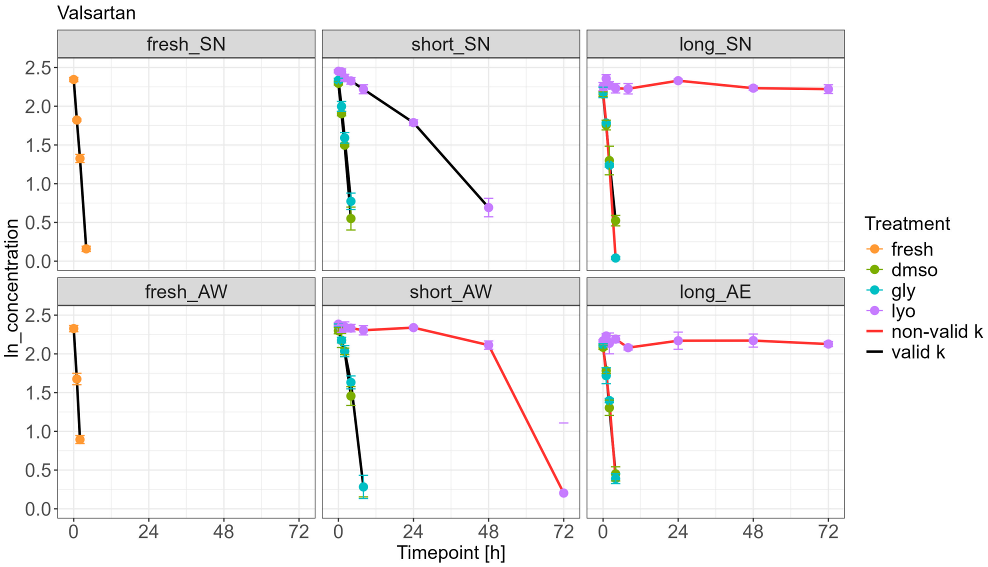

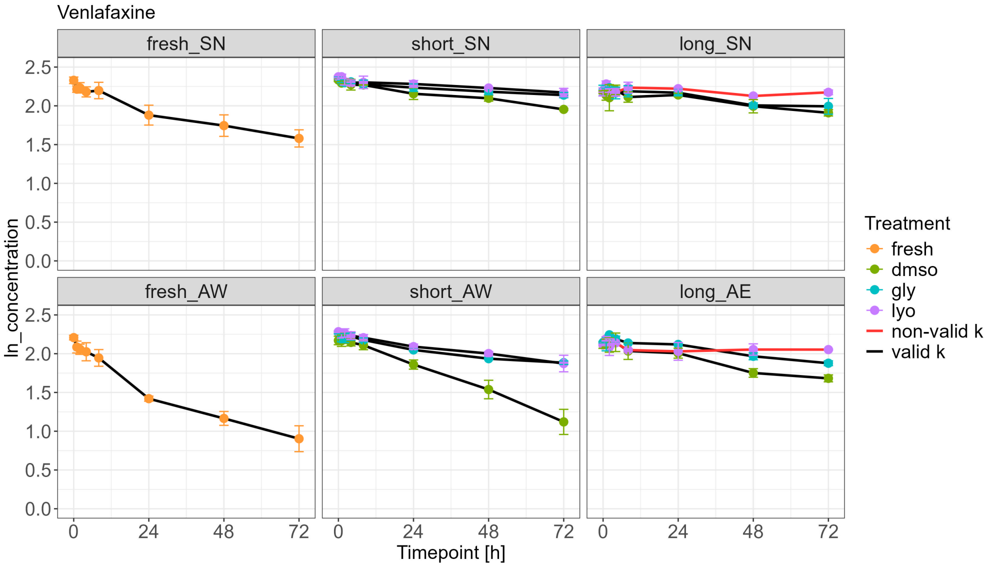

Fig. S8: Linearized concentration-time series per treatment (fresh: yellow, LYO: purple, GLY: blue, and DMSO: green) averaged over the experimental triplicates, separated in columns for the three experiment (fresh, short, and long) and into rows for the use of native media (SN, top) and artificial media (AW or AE, bottom). Time-point linking line is depicted in red, if the derived rate constant does not fulfill the quality requirements (non-valid is TRUE), else black.

S5 Biotransformation Profiling

S5.1 Validation Parameters for First-Order Rate Constants Derivation

Measurement issues were excluded by visual inspection of each data point and $C_t \geq$ LOQ verification. At the test substance level, it was confirmed visually that all spiked C_0 were measured closely to 10 nM, and the test substances did not exhibit strong sorption behavior (dissipation < 20 % in SC experiments over 48 h). Visual inspection of each concentration-time series of each micropollutant (MP) did not reveal any peculiar concentration evolution over time (e.g., lag phase, poor goodness of fit). For the applicability of the kinetic model on the linearized data (Equ. 5), at least three points in time were quantifiable. Model validation was performed with the following criteria: $R^2 \geq 0.85$, $p \leq 0.05$, and $k > k_{\mathrm{lim}}$. At a treatment level, averaging was allowed if the number of replicates with valid rate constants ≥ 2.

$$ln(C_t) = -kt + ln(C_0) \tag{5}$$

S5.2 Results of Biotransformation Profiling

With an average coefficient of variation of 6 %, analytical measurements of test substance concentrations were fairly precise. The corresponding minimal rate constant that could reliably be quantified given experimental, instrumental and measurement evaluation uncertainty was set at $\mathrm{k_{lim}} = 0.0015\,\mathrm{h^{-1}}$, corresponding to less than 10 % dissipation over the experimental time of 72 h. An indicative maximal quantifiable rate constant was estimated based on the average LOQ of test substances (0.5 nM), and was determined to be $\mathrm{k_{max}} = 1.1513\,\mathrm{h^{-1}}$. Concentration-time series of the test substances are illustrated in the S4. All chosen test substances were known to not degrade abiotically.[27,28]

Among the 36 test substances, five MPs the biotransformation kinetics were not further evaluated (Tbl. S13). Included as a positive control for sorption, climbazole showed an av-

erage depletion of more than 25 % after 48 h in SC experiments. Similar sorption behavior was observed only for acemetacin in experiments using AW as the resuspension medium; therefore, this biotransformation rate was not further evaluated. No other sorption behavior for the other test substances was detected in our experimental matrices. Further, the initial spiked concentration of dextrometorphan was significantly lower than the nominal spiked concentration (5.7 ± 1.6 nM against 10 nM). The concentration-time series of emtricitabine and levetiracetam suggested a lag-phase preceding a fast depletion which was not well captured by the sampling scheme resolution. Last, atrazine, which was included as a control substance for persistence, was removed from further evaluation, as it never achieved a higher rate constant than k_{lim}. Tbl. S13 summarizes substances exluded from biotransformation kinetics analysis.

Tbl. S13: MPs excluded from biotransformation kinetics evaluation

**Substance**	**Exclusion criteria**
Atrazine[a]	Persistent
Climbazole[a]	Strongly sorbing
Dextromethorphan	Initial concentration much lower than nominal spiked concentration
Emtricitabine	Concentration-time series inconsistent with first-order kinetics
Levetiracetam	Concentration-time series inconsistent with first-order kinetics

[a] Substance included as control.

Summarized, all of these validation criteria had to be met for each replicate for further consideration:

- Initial spiked concentration C_0 close to 10 nM.
- Dissipation < 20 % in SC experiments over 48 h.
- Faster degradation than $k_{lim} = 0.0015\,h^{-1}$ (= not persistent).
- Biotransformation well captured by experimental time resolution (no lag phase, visual inspection of concentration-time series and goodness of fit).

- $R^2 \geq 0.85$ (sufficiently strong relationship between measured and predicted concentrations).
- $p \leq 0.05$ (statistically significant relationship between concentration and time).
- $k \geq k_{\text{lim}}$ (data uncertainty is not the sole factor explaining depletion, dissipation > 10 % over 72 h).
- $k \leq k_{\text{max}} = 1.1513\,\text{h}^{-1}$ (at least three data points retrieved before LOQ is reached and dissipation < 90 % within 2 h).

The resulting rate constants averaged over three replicates for each micropollutant and each treatment are summarized in the following table Tbl. S14 and heatmap Fig. S9.

Tbl. S14: Absolute rate constants averaged over three replicates for each micropollutant and each treatment.

Substance	Preservation method	k [h^{-1}]	R^2	p	$t_{0.5}$ [d^{-1}]	k_{max} [a]	k_{min} [b]	non-valid [c]
Acemetacin	f_fresh_SN	0.273	0.992	***	2.54	FALSE	FALSE	FALSE
	f_fresh_AW	0.1894	0.999	***	3.66	FALSE	FALSE	FALSE
	short_lyo_SN	0.3619	0.994	**	1.92	FALSE	FALSE	FALSE
	short_lyo_AW	0.1846	0.986	***	3.76	FALSE	FALSE	FALSE
	short_dmso_SN	0.2817	0.998	***	2.46	FALSE	FALSE	FALSE
	short_dmso_AW	0.1418	0.991	***	4.89	FALSE	FALSE	FALSE
	short_gly_SN	0.279	0.999	***	2.48	FALSE	FALSE	FALSE
	short_gly_AW	0.1788	0.998	***	3.88	FALSE	FALSE	FALSE
	long_lyo_SN	0.2292	0.988	***	3.02	FALSE	FALSE	FALSE
	long_lyo_AE	0.327	0.998	***	2.12	FALSE	FALSE	FALSE
	long_dmso_SN	0.3114	0.993	***	2.23	FALSE	FALSE	FALSE
	long_dmso_AE	0.2783	0.989	***	2.49	FALSE	FALSE	FALSE
	long_gly_SN	0.3072	0.996	***	2.26	FALSE	FALSE	FALSE
	long_gly_AE	0.2972	0.985	***	2.33	FALSE	FALSE	FALSE
Acesulfame	f_fresh_SN	2.4066	1	*NA*	0.29	TRUE	FALSE	FALSE
	f_fresh_AW	1.6776	1	*NA*	0.41	TRUE	FALSE	FALSE
	short_lyo_SN	1.4945	1	*NA*	0.46	TRUE	FALSE	FALSE
	short_lyo_AW	1.021	1	*	0.68	FALSE	FALSE	FALSE
	short_dmso_SN	2.2562	1	*NA*	0.31	TRUE	FALSE	FALSE
	short_dmso_AW	1.2635	1	*NA*	0.55	TRUE	FALSE	FALSE
	short_gly_SN	2.0284	1	*NA*	0.34	TRUE	FALSE	FALSE
	short_gly_AW	1.3767	1	*NA*	0.5	TRUE	FALSE	FALSE
	long_lyo_SN	0.2251	0.991	***	3.08	FALSE	FALSE	FALSE
	long_lyo_AE	0.1026	0.999	***	6.76	FALSE	FALSE	FALSE
	long_dmso_SN	1.1846	0.849	*ns*	0.59	TRUE	FALSE	TRUE
	long_dmso_AE	1.1007	0.912	*	0.63	FALSE	FALSE	FALSE
	long_gly_SN	2.0329	0.996	*	0.34	TRUE	FALSE	FALSE

Continued on next page

Tbl. S14: Rate constants averaged over three replicates for each micropollutant and each treatment. (continued)

**Substance**	**Preservation method**	**k** $[h^{-1}]$	$\mathbf{R^2}$	**p**	$\mathbf{t_{0.5}}$ $[d^{-1}]$	$\mathbf{k_{max}}$ [a]	$\mathbf{k_{min}}$ [b]	**non-valid** [c]
	long_gly_AE	1.5728	1	**	0.44	TRUE	FALSE	FALSE
Alachlor	f_fresh_SN	0.206	0.998	***	3.37	FALSE	FALSE	FALSE
	f_fresh_AW	0.2138	1	***	3.24	FALSE	FALSE	FALSE
	short_lyo_SN	0.0474	0.997	***	14.63	FALSE	FALSE	FALSE
	short_lyo_AW	0.0411	0.993	***	16.88	FALSE	FALSE	FALSE
	short_dmso_SN	0.1932	0.999	***	3.59	FALSE	FALSE	FALSE
	short_dmso_AW	0.164	0.997	***	4.23	FALSE	FALSE	FALSE
	short_gly_SN	0.1893	0.999	***	3.66	FALSE	FALSE	FALSE
	short_gly_AW	0.1639	0.998	***	4.23	FALSE	FALSE	FALSE
	long_lyo_SN	0.0151	0.927	***	45.94	FALSE	FALSE	FALSE
	long_lyo_AE	0.0219	0.924	***	31.66	FALSE	FALSE	FALSE
	long_dmso_SN	0.2055	0.995	***	3.37	FALSE	FALSE	FALSE
	long_dmso_AE	0.1932	0.994	***	3.59	FALSE	FALSE	FALSE
	long_gly_SN	0.2146	0.996	***	3.23	FALSE	FALSE	FALSE
	long_gly_AE	0.2043	0.984	***	3.39	FALSE	FALSE	FALSE
Albuterol	f_fresh_SN	0.0603	0.998	***	11.49	FALSE	FALSE	FALSE
	f_fresh_AW	0.0652	0.997	***	10.63	FALSE	FALSE	FALSE
	short_lyo_SN	0.0353	0.983	***	19.61	FALSE	FALSE	FALSE
	short_lyo_AW	0.0239	0.988	***	28.95	FALSE	FALSE	FALSE
	short_dmso_SN	0.0632	0.993	***	10.97	FALSE	FALSE	FALSE
	short_dmso_AW	0.0533	0.997	***	13.02	FALSE	FALSE	FALSE
	short_gly_SN	0.0557	0.993	***	12.44	FALSE	FALSE	FALSE
	short_gly_AW	0.0522	0.995	***	13.27	FALSE	FALSE	FALSE
	long_lyo_SN	0.0102	0.918	***	68.23	FALSE	FALSE	FALSE
	long_lyo_AE	0.0083	0.954	***	83.22	FALSE	FALSE	FALSE
	long_dmso_SN	0.0734	0.991	***	9.44	FALSE	FALSE	FALSE
	long_dmso_AE	0.0639	0.995	***	10.84	FALSE	FALSE	FALSE

Continued on next page

Tbl. S14: Rate constants averaged over three replicates for each micropollutant and each treatment. (continued)

**Substance**	**Preservation method**	**k** [h^{-1}]	$\mathbf{R^2}$	**p**	$\mathbf{t_{0.5}}$ [d^{-1}]	$\mathbf{k_{max}}$ [a]	$\mathbf{k_{min}}$ [b]	**non-valid** [c]
	long_gly_SN	0.0701	0.995	$***$	9.89	FALSE	FALSE	FALSE
	long_gly_AE	0.0605	0.995	$***$	11.45	FALSE	FALSE	FALSE
Atenolol	f_fresh_SN	0.1899	0.999	$***$	3.65	FALSE	FALSE	FALSE
	f_fresh_AW	0.3791	0.999	$***$	1.83	FALSE	FALSE	FALSE
	short_lyo_SN	0.2228	0.999	$***$	3.11	FALSE	FALSE	FALSE
	short_lyo_AW	0.2407	0.999	$***$	2.88	FALSE	FALSE	FALSE
	short_dmso_SN	0.169	0.999	$***$	4.1	FALSE	FALSE	FALSE
	short_dmso_AW	0.4628	0.999	$***$	1.5	FALSE	FALSE	FALSE
	short_gly_SN	0.2126	1	$***$	3.26	FALSE	FALSE	FALSE
	short_gly_AW	0.3952	1	$***$	1.75	FALSE	FALSE	FALSE
	long_lyo_SN	0.1079	0.997	$***$	6.42	FALSE	FALSE	FALSE
	long_lyo_AE	0.2071	0.997	$***$	3.35	FALSE	FALSE	FALSE
	long_dmso_SN	0.1755	0.992	$***$	3.95	FALSE	FALSE	FALSE
	long_dmso_AE	0.2627	0.996	$***$	2.64	FALSE	FALSE	FALSE
	long_gly_SN	0.2219	0.997	$***$	3.12	FALSE	FALSE	FALSE
	long_gly_AE	0.2303	0.993	$***$	3.01	FALSE	FALSE	FALSE
Atorvastatin	f_fresh_SN	1.2321	0.998	$*$	0.56	TRUE	FALSE	FALSE
	f_fresh_AW	1.0205	0.997	$*$	0.68	FALSE	FALSE	FALSE
	short_lyo_SN	0.12	0.989	$***$	5.77	FALSE	FALSE	FALSE
	short_lyo_AW	0.0456	0.97	$***$	15.21	FALSE	FALSE	FALSE
	short_dmso_SN	1.2404	0.991	*ns*	0.56	TRUE	FALSE	TRUE
	short_dmso_AW	0.6784	0.994	$*$	1.02	FALSE	FALSE	FALSE
	short_gly_SN	1.2194	0.997	$*$	0.57	TRUE	FALSE	FALSE
	short_gly_AW	0.937	0.994	$*$	0.74	FALSE	FALSE	FALSE
	long_lyo_SN	0.0026	0.44	*ns*	266.23	FALSE	FALSE	TRUE
	long_lyo_AE	0.0041	0.822	$**$	170.66	FALSE	FALSE	TRUE
	long_dmso_SN	1.1939	0.995	$*$	0.58	TRUE	FALSE	FALSE

Continued on next page

Tbl. S14: Rate constants averaged over three replicates for each micropollutant and each treatment. (continued)

**Substance**	**Preservation method**	**k** [h^{-1}]	R^2	**p**	$t_{0.5}$ [d^{-1}]	k_{max} [a]	k_{min} [b]	**non-valid** [c]
	long_dmso_AE	1.0418	0.991	*ns*	0.67	FALSE	FALSE	TRUE
	long_gly_SN	1.1625	0.991	*ns*	0.6	TRUE	FALSE	TRUE
	long_gly_AE	1.1193	0.998	*	0.62	FALSE	FALSE	FALSE
Bezafibrate	f_fresh_SN	0.3555	0.998	***	1.95	FALSE	FALSE	FALSE
	f_fresh_AW	0.3518	0.995	***	1.97	FALSE	FALSE	FALSE
	short_lyo_SN	0.0528	0.986	***	13.13	FALSE	FALSE	FALSE
	short_lyo_AW	0.0234	0.981	***	29.63	FALSE	FALSE	FALSE
	short_dmso_SN	0.4067	0.997	***	1.7	FALSE	FALSE	FALSE
	short_dmso_AW	0.3429	0.995	***	2.02	FALSE	FALSE	FALSE
	short_gly_SN	0.3689	0.999	***	1.88	FALSE	FALSE	FALSE
	short_gly_AW	0.3752	0.999	***	1.85	FALSE	FALSE	FALSE
	long_lyo_SN	0.0027	0.688	*	258.71	FALSE	FALSE	TRUE
	long_lyo_AE	0.002	0.497	*ns*	342.27	FALSE	FALSE	TRUE
	long_dmso_SN	0.3933	0.992	***	1.76	FALSE	FALSE	FALSE
	long_dmso_AE	0.47	0.988	***	1.47	FALSE	FALSE	FALSE
	long_gly_SN	0.3928	0.997	***	1.76	FALSE	FALSE	FALSE
	long_gly_AE	0.4452	0.984	***	1.56	FALSE	FALSE	FALSE
Carbendazim	f_fresh_SN	0.1484	0.999	***	4.67	FALSE	FALSE	FALSE
	f_fresh_AW	0.1961	0.998	***	3.54	FALSE	FALSE	FALSE
	short_lyo_SN	0.1629	0.999	***	4.26	FALSE	FALSE	FALSE
	short_lyo_AW	0.1588	0.996	***	4.36	FALSE	FALSE	FALSE
	short_dmso_SN	0.1504	0.999	***	4.61	FALSE	FALSE	FALSE
	short_dmso_AW	0.1931	0.993	***	3.59	FALSE	FALSE	FALSE
	short_gly_SN	0.1553	0.999	***	4.46	FALSE	FALSE	FALSE
	short_gly_AW	0.1983	0.997	***	3.5	FALSE	FALSE	FALSE
	long_lyo_SN	0.0996	0.997	***	6.96	FALSE	FALSE	FALSE
	long_lyo_AE	0.1058	0.994	***	6.55	FALSE	FALSE	FALSE

Continued on next page

Tbl. S14: Rate constants averaged over three replicates for each micropollutant and each treatment. (continued)

**Substance**	**Preservation method**	**k** [h^{-1}]	$\mathbf{R^2}$	**p**	$\mathbf{t_{0.5}}$ [d^{-1}]	$\mathbf{k_{max}}$[a]	$\mathbf{k_{min}}$[b]	**non-valid**[c]
	long_dmso_SN	0.1797	0.998	$***$	3.86	FALSE	FALSE	FALSE
	long_dmso_AE	0.2166	0.997	$***$	3.2	FALSE	FALSE	FALSE
	long_gly_SN	0.1962	0.997	$***$	3.53	FALSE	FALSE	FALSE
	long_gly_AE	0.2085	0.995	$***$	3.33	FALSE	FALSE	FALSE
Chlorotoluron	f_fresh_SN	0.0122	0.991	$***$	57.01	FALSE	FALSE	FALSE
	f_fresh_AW	0.0073	0.969	$***$	94.99	FALSE	FALSE	FALSE
	short_lyo_SN	$9e-04$	0.359	*ns*	772.53	FALSE	TRUE	TRUE
	short_lyo_AW	$5e-04$	0.268	*ns*	1442.41	FALSE	TRUE	TRUE
	short_dmso_SN	0.0094	0.984	$***$	73.6	FALSE	FALSE	FALSE
	short_dmso_AW	0.0033	0.841	$**$	210.91	FALSE	FALSE	TRUE
	short_gly_SN	0.0079	0.99	$***$	87.5	FALSE	FALSE	FALSE
	short_gly_AW	0.0028	0.793	$**$	247.89	FALSE	FALSE	TRUE
	long_lyo_SN	0	0	*ns*	542239.8	FALSE	TRUE	TRUE
	long_lyo_AE	$-1e-04$	0.01	*ns*	-4860.31	FALSE	TRUE	TRUE
	long_dmso_SN	0.0107	0.958	$***$	64.94	FALSE	FALSE	FALSE
	long_dmso_AE	0.0101	0.958	$***$	68.79	FALSE	FALSE	FALSE
	long_gly_SN	0.0093	0.992	$***$	74.76	FALSE	FALSE	FALSE
	long_gly_AE	0.0066	0.932	$***$	105.04	FALSE	FALSE	FALSE
DEET	f_fresh_SN	0.2667	0.996	$***$	2.6	FALSE	FALSE	FALSE
	f_fresh_AW	0.2373	0.996	$***$	2.92	FALSE	FALSE	FALSE
	short_lyo_SN	0.0106	0.99	$***$	65.48	FALSE	FALSE	FALSE
	short_lyo_AW	0.0078	0.976	$***$	88.62	FALSE	FALSE	FALSE
	short_dmso_SN	0.2848	0.995	$***$	2.43	FALSE	FALSE	FALSE
	short_dmso_AW	0.2576	0.985	$***$	2.69	FALSE	FALSE	FALSE
	short_gly_SN	0.3107	0.992	$***$	2.23	FALSE	FALSE	FALSE
	short_gly_AW	0.305	0.994	$***$	2.27	FALSE	FALSE	FALSE
	long_lyo_SN	$6e-04$	0.101	*ns*	1257.59	FALSE	TRUE	TRUE

Continued on next page

Tbl. S14: Rate constants averaged over three replicates for each micropollutant and each treatment. (continued)

**Substance**	**Preservation method**	**k** [h^{-1}]	$\mathbf{R^2}$	**p**	$\mathbf{t_{0.5}}$ [d^{-1}]	$\mathbf{k_{max}}$ [a]	$\mathbf{k_{min}}$ [b]	**non-valid** [c]
	long_lyo_AE	$4e-04$	0.056	*ns*	1744.54	FALSE	TRUE	TRUE
	long_dmso_SN	0.1747	0.985	∗∗∗	3.97	FALSE	FALSE	FALSE
	long_dmso_AE	0.1676	0.968	∗∗∗	4.14	FALSE	FALSE	FALSE
	long_gly_SN	0.2415	0.996	∗∗∗	2.87	FALSE	FALSE	FALSE
	long_gly_AE	0.1981	0.982	∗∗∗	3.5	FALSE	FALSE	FALSE
Dimethenamid	f_fresh_SN	0.0281	0.998	∗∗∗	24.68	FALSE	FALSE	FALSE
	f_fresh_AW	0.0323	0.998	∗∗∗	21.47	FALSE	FALSE	FALSE
	short_lyo_SN	0.0101	0.992	∗∗∗	68.35	FALSE	FALSE	FALSE
	short_lyo_AW	0.0098	0.995	∗∗∗	70.64	FALSE	FALSE	FALSE
	short_dmso_SN	0.0244	0.998	∗∗∗	28.42	FALSE	FALSE	FALSE
	short_dmso_AW	0.0257	0.997	∗∗∗	27	FALSE	FALSE	FALSE
	short_gly_SN	0.0236	0.997	∗∗∗	29.32	FALSE	FALSE	FALSE
	short_gly_AW	0.0247	0.996	∗∗∗	28.01	FALSE	FALSE	FALSE
	long_lyo_SN	0.0022	0.65	∗	310.11	FALSE	FALSE	TRUE
	long_lyo_AE	0.0018	0.686	∗	380.62	FALSE	FALSE	TRUE
	long_dmso_SN	0.0262	0.997	∗∗∗	26.44	FALSE	FALSE	FALSE
	long_dmso_AE	0.0283	0.995	∗∗∗	24.47	FALSE	FALSE	FALSE
	long_gly_SN	0.0271	0.996	∗∗∗	25.57	FALSE	FALSE	FALSE
	long_gly_AE	0.0275	0.995	∗∗∗	25.24	FALSE	FALSE	FALSE
Furosemide	f_fresh_SN	0.0352	0.996	∗∗∗	19.7	FALSE	FALSE	FALSE
	f_fresh_AW	0.031	0.986	∗∗∗	22.34	FALSE	FALSE	FALSE
	short_lyo_SN	0.0034	0.916	∗∗∗	201.52	FALSE	FALSE	FALSE
	short_lyo_AW	0.0023	0.878	∗∗∗	295.35	FALSE	FALSE	FALSE
	short_dmso_SN	0.0347	0.993	∗∗∗	20	FALSE	FALSE	FALSE
	short_dmso_AW	0.0185	0.99	∗∗∗	37.47	FALSE	FALSE	FALSE
	short_gly_SN	0.0304	0.994	∗∗∗	22.79	FALSE	FALSE	FALSE
	short_gly_AW	0.0239	0.982	∗∗∗	28.96	FALSE	FALSE	FALSE

Continued on next page

Tbl. S14: Rate constants averaged over three replicates for each micropollutant and each treatment. (continued)

**Substance**	**Preservation method**	**k** [h^{-1}]	$\mathbf{R^2}$	**p**	$\mathbf{t_{0.5}}$ [d^{-1}]	$\mathbf{k_{max}}$[a]	$\mathbf{k_{min}}$[b]	**non-valid**[c]
	long_lyo_SN	0.001	0.265	*ns*	716.13	FALSE	TRUE	TRUE
	long_lyo_AE	$3e-04$	0.045	*ns*	2194.46	FALSE	TRUE	TRUE
	long_dmso_SN	0.0464	0.991	***	14.93	FALSE	FALSE	FALSE
	long_dmso_AE	0.04	0.994	***	17.31	FALSE	FALSE	FALSE
	long_gly_SN	0.0411	0.988	***	16.85	FALSE	FALSE	FALSE
	long_gly_AE	0.037	0.991	***	18.75	FALSE	FALSE	FALSE
Ketamine	f_fresh_SN	0.0135	0.948	***	51.42	FALSE	FALSE	FALSE
	f_fresh_AW	0.014	0.963	***	49.59	FALSE	FALSE	FALSE
	short_lyo_SN	0.0015	0.542	*	461.59	FALSE	FALSE	TRUE
	short_lyo_AW	0.0016	0.856	***	422.87	FALSE	FALSE	FALSE
	short_dmso_SN	0.0052	0.961	***	132.36	FALSE	FALSE	FALSE
	short_dmso_AW	0.0051	0.938	***	137.1	FALSE	FALSE	FALSE
	short_gly_SN	0.0041	0.943	***	169.77	FALSE	FALSE	FALSE
	short_gly_AW	0.0035	0.861	***	200.26	FALSE	FALSE	FALSE
	long_lyo_SN	0	0	*ns*	21838.12	FALSE	TRUE	TRUE
	long_lyo_AE	0	0.001	*ns*	−14360.98	FALSE	TRUE	TRUE
	long_dmso_SN	0.005	0.934	***	138.93	FALSE	FALSE	FALSE
	long_dmso_AE	0.0058	0.94	***	119.02	FALSE	FALSE	FALSE
	long_gly_SN	0.0043	0.909	***	161.12	FALSE	FALSE	FALSE
	long_gly_AE	0.0039	0.883	***	178.63	FALSE	FALSE	FALSE
Ketoprofen	f_fresh_SN	0.9229	1	**	0.75	FALSE	FALSE	FALSE
	f_fresh_AW	0.8192	0.998	*	0.85	FALSE	FALSE	FALSE
	short_lyo_SN	0.0852	0.995	***	8.14	FALSE	FALSE	FALSE
	short_lyo_AW	0.0372	0.948	***	18.65	FALSE	FALSE	FALSE
	short_dmso_SN	1.0278	0.993	*ns*	0.67	FALSE	FALSE	TRUE
	short_dmso_AW	0.6267	0.994	*	1.11	FALSE	FALSE	FALSE
	short_gly_SN	0.924	0.997	*	0.75	FALSE	FALSE	FALSE

Continued on next page

Tbl. S14: Rate constants averaged over three replicates for each micropollutant and each treatment. (continued)

**Substance**	**Preservation method**	**k** [h^{-1}]	$\mathbf{R^2}$	**p**	$\mathbf{t_{0.5}}$ [d^{-1}]	$\mathbf{k_{max}}$[a]	$\mathbf{k_{min}}$[b]	**non-valid**[c]
	short_gly_AW	0.7484	0.996	$*$	0.93	FALSE	FALSE	FALSE
	long_lyo_SN	$-5e-04$	0.139	ns	−1485.98	FALSE	TRUE	TRUE
	long_lyo_AE	$5e-04$	0.081	ns	1516.2	FALSE	TRUE	TRUE
	long_dmso_SN	0.1689	0.502	ns	4.1	FALSE	FALSE	TRUE
	long_dmso_AE	0.7559	0.889	$*$	0.92	FALSE	FALSE	FALSE
	long_gly_SN	0.6691	0.911	$*$	1.04	FALSE	FALSE	FALSE
	long_gly_AE	1.2657	0.989	$**$	0.55	TRUE	FALSE	FALSE
Losartan	f_fresh_SN	0.2322	0.999	$***$	2.99	FALSE	FALSE	FALSE
	f_fresh_AW	0.1278	1	$***$	5.43	FALSE	FALSE	FALSE
	short_lyo_SN	0.0473	0.961	$***$	14.67	FALSE	FALSE	FALSE
	short_lyo_AW	0.0235	0.984	$***$	29.44	FALSE	FALSE	FALSE
	short_dmso_SN	0.2822	0.999	$***$	2.46	FALSE	FALSE	FALSE
	short_dmso_AW	0.1138	0.996	$***$	6.09	FALSE	FALSE	FALSE
	short_gly_SN	0.2871	0.999	$***$	2.41	FALSE	FALSE	FALSE
	short_gly_AW	0.1227	0.999	$***$	5.65	FALSE	FALSE	FALSE
	long_lyo_SN	0.0021	0.474	ns	326.91	FALSE	FALSE	TRUE
	long_lyo_AE	0.0031	0.714	$**$	225.34	FALSE	FALSE	TRUE
	long_dmso_SN	0.2316	0.999	$***$	2.99	FALSE	FALSE	FALSE
	long_dmso_AE	0.1984	0.998	$***$	3.49	FALSE	FALSE	FALSE
	long_gly_SN	0.3001	0.997	$***$	2.31	FALSE	FALSE	FALSE
	long_gly_AE	0.216	0.994	$***$	3.21	FALSE	FALSE	FALSE
Mecoprop	f_fresh_SN	0.0314	0.972	$***$	22.09	FALSE	FALSE	FALSE
	f_fresh_AW	0.0166	0.95	$***$	41.82	FALSE	FALSE	FALSE
	short_lyo_SN	0.0053	0.969	$***$	131.48	FALSE	FALSE	FALSE
	short_lyo_AW	0.0012	0.766	$**$	588.49	FALSE	TRUE	TRUE
	short_dmso_SN	0.038	0.975	$***$	18.24	FALSE	FALSE	FALSE
	short_dmso_AW	0.012	0.931	$***$	57.59	FALSE	FALSE	FALSE

Continued on next page

Tbl. S14: Rate constants averaged over three replicates for each micropollutant and each treatment. (continued)

**Substance**	**Preservation method**	**k** [h^{-1}]	$\mathbf{R^2}$	**p**	$\mathbf{t_{0.5}}$ [d^{-1}]	$\mathbf{k_{max}}$[a]	$\mathbf{k_{min}}$[b]	**non-valid**[c]
	short_gly_SN	0.0294	0.955	$***$	23.61	FALSE	FALSE	FALSE
	short_gly_AW	0.0129	0.897	$***$	53.7	FALSE	FALSE	FALSE
	long_lyo_SN	$-3e-04$	0.037	*ns*	−2381.36	FALSE	TRUE	TRUE
	long_lyo_AE	0	0	*ns*	41487.93	FALSE	TRUE	TRUE
	long_dmso_SN	0.0411	0.986	$***$	16.88	FALSE	FALSE	FALSE
	long_dmso_AE	0.0431	0.909	$***$	16.08	FALSE	FALSE	FALSE
	long_gly_SN	0.0339	0.98	$***$	20.46	FALSE	FALSE	FALSE
	long_gly_AE	0.0451	0.919	$***$	15.37	FALSE	FALSE	FALSE
Metoprolol	f_fresh_SN	0.1297	0.975	$***$	5.34	FALSE	FALSE	FALSE
	f_fresh_AW	0.1007	0.99	$***$	6.88	FALSE	FALSE	FALSE
	short_lyo_SN	0.0512	0.932	$***$	13.55	FALSE	FALSE	FALSE
	short_lyo_AW	0.0622	0.987	$***$	11.15	FALSE	FALSE	FALSE
	short_dmso_SN	0.1121	0.989	$***$	6.19	FALSE	FALSE	FALSE
	short_dmso_AW	0.1694	0.994	$***$	4.09	FALSE	FALSE	FALSE
	short_gly_SN	0.1122	0.993	$***$	6.18	FALSE	FALSE	FALSE
	short_gly_AW	0.1776	0.98	$***$	3.9	FALSE	FALSE	FALSE
	long_lyo_SN	0.0126	0.91	$***$	55.01	FALSE	FALSE	FALSE
	long_lyo_AE	0.013	0.975	$***$	53.23	FALSE	FALSE	FALSE
	long_dmso_SN	0.1111	0.987	$***$	6.24	FALSE	FALSE	FALSE
	long_dmso_AE	0.121	0.988	$***$	5.73	FALSE	FALSE	FALSE
	long_gly_SN	0.1289	0.994	$***$	5.38	FALSE	FALSE	FALSE
	long_gly_AE	0.1395	0.989	$***$	4.97	FALSE	FALSE	FALSE
Mexiletine	f_fresh_SN	0.0483	0.947	$***$	14.36	FALSE	FALSE	FALSE
	f_fresh_AW	0.0898	0.99	$***$	7.72	FALSE	FALSE	FALSE
	short_lyo_SN	0.0159	0.975	$***$	43.59	FALSE	FALSE	FALSE
	short_lyo_AW	0.0237	0.94	$***$	29.3	FALSE	FALSE	FALSE
	short_dmso_SN	0.0303	0.979	$***$	22.9	FALSE	FALSE	FALSE

Continued on next page

Tbl. S14: Rate constants averaged over three replicates for each micropollutant and each treatment. (continued)

**Substance**	**Preservation method**	**k** [h^{-1}]	$\mathbf{R^2}$	**p**	$\mathbf{t_{0.5}}$ [d^{-1}]	$\mathbf{k_{max}}$ [a]	$\mathbf{k_{min}}$ [b]	**non-valid** [c]
	short_dmso_AW	0.0712	0.99	***	9.73	FALSE	FALSE	FALSE
	short_gly_SN	0.0248	0.946	***	27.95	FALSE	FALSE	FALSE
	short_gly_AW	0.045	0.971	***	15.41	FALSE	FALSE	FALSE
	long_lyo_SN	0.0095	0.956	***	72.92	FALSE	FALSE	FALSE
	long_lyo_AE	0.0097	0.969	***	71.16	FALSE	FALSE	FALSE
	long_dmso_SN	0.0315	0.993	***	21.97	FALSE	FALSE	FALSE
	long_dmso_AE	0.0455	0.987	***	15.22	FALSE	FALSE	FALSE
	long_gly_SN	0.0264	0.961	***	26.24	FALSE	FALSE	FALSE
	long_gly_AE	0.0316	0.981	***	21.9	FALSE	FALSE	FALSE
Pravastatin	f_fresh_SN	2.6525	1	*NA*	0.26	TRUE	FALSE	FALSE
	f_fresh_AW	2.1839	1	*NA*	0.32	TRUE	FALSE	FALSE
	short_lyo_SN	0.5889	0.998	***	1.18	FALSE	FALSE	FALSE
	short_lyo_AW	0.1713	0.996	***	4.05	FALSE	FALSE	FALSE
	short_dmso_SN	2.3744	1	*NA*	0.29	TRUE	FALSE	FALSE
	short_dmso_AW	1.5195	1	*NA*	0.46	TRUE	FALSE	FALSE
	short_gly_SN	2.3627	1	*NA*	0.29	TRUE	FALSE	FALSE
	short_gly_AW	1.8607	1	*NA*	0.37	TRUE	FALSE	FALSE
	long_lyo_SN	0.0796	0.975	***	8.7	FALSE	FALSE	FALSE
	long_lyo_AE	0.0754	0.983	***	9.19	FALSE	FALSE	FALSE
	long_dmso_SN	1.2982	0.891	*ns*	0.53	TRUE	FALSE	TRUE
	long_dmso_AE	1.4542	0.936	*	0.48	TRUE	FALSE	FALSE
	long_gly_SN	2.2461	0.998	*	0.31	TRUE	FALSE	FALSE
	long_gly_AE	2.4735	1	**	0.28	TRUE	FALSE	FALSE
Propachlor	f_fresh_SN	1.7608	1	*NA*	0.39	TRUE	FALSE	FALSE
	f_fresh_AW	1.8797	1	*NA*	0.37	TRUE	FALSE	FALSE
	short_lyo_SN	0.3933	0.998	**	1.76	FALSE	FALSE	FALSE
	short_lyo_AW	0.3303	0.999	***	2.1	FALSE	FALSE	FALSE

Continued on next page

Tbl. S14: Rate constants averaged over three replicates for each micropollutant and each treatment. (continued)

**Substance**	**Preservation method**	**k** [h^{-1}]	$\mathbf{R^2}$	**p**	$\mathbf{t_{0.5}}$ [d^{-1}]	$\mathbf{k_{max}}$ [a]	$\mathbf{k_{min}}$ [b]	**non-valid** [c]
	short_dmso_SN	1.4637	1	*NA*	0.47	TRUE	FALSE	FALSE
	short_dmso_AW	1.2862	1	*NA*	0.54	TRUE	FALSE	FALSE
	short_gly_SN	1.2124	1	*NA*	0.57	TRUE	FALSE	FALSE
	short_gly_AW	1.2231	1	*NA*	0.57	TRUE	FALSE	FALSE
	long_lyo_SN	0.0866	0.994	***	8	FALSE	FALSE	FALSE
	long_lyo_AE	0.102	0.996	***	6.8	FALSE	FALSE	FALSE
	long_dmso_SN	1.5474	0.998	*	0.45	TRUE	FALSE	FALSE
	long_dmso_AE	1.4445	0.995	*	0.48	TRUE	FALSE	FALSE
	long_gly_SN	1.1605	0.999	*	0.6	TRUE	FALSE	FALSE
	long_gly_AE	1.1888	1	**	0.58	TRUE	FALSE	FALSE
Ranitidine	f_fresh_SN	0.0715	0.99	***	9.69	FALSE	FALSE	FALSE
	f_fresh_AW	0.0648	0.995	***	10.7	FALSE	FALSE	FALSE
	short_lyo_SN	0.0287	0.996	***	24.15	FALSE	FALSE	FALSE
	short_lyo_AW	0.0129	0.992	***	53.94	FALSE	FALSE	FALSE
	short_dmso_SN	0.0731	0.994	***	9.48	FALSE	FALSE	FALSE
	short_dmso_AW	0.0546	0.992	***	12.7	FALSE	FALSE	FALSE
	short_gly_SN	0.0722	0.995	***	9.6	FALSE	FALSE	FALSE
	short_gly_AW	0.0493	0.994	***	14.05	FALSE	FALSE	FALSE
	long_lyo_SN	0.0121	0.98	***	57.25	FALSE	FALSE	FALSE
	long_lyo_AE	0.008	0.961	***	86.24	FALSE	FALSE	FALSE
	long_dmso_SN	0.0651	0.985	***	10.65	FALSE	FALSE	FALSE
	long_dmso_AE	0.0645	0.987	***	10.75	FALSE	FALSE	FALSE
	long_gly_SN	0.0649	0.97	***	10.67	FALSE	FALSE	FALSE
	long_gly_AE	0.0586	0.992	***	11.84	FALSE	FALSE	FALSE
Rufinamide	f_fresh_SN	0.2156	0.995	***	3.21	FALSE	FALSE	FALSE
	f_fresh_AW	0.2567	0.995	***	2.7	FALSE	FALSE	FALSE
	short_lyo_SN	0.0838	0.995	***	8.27	FALSE	FALSE	FALSE

Continued on next page

Tbl. S14: Rate constants averaged over three replicates for each micropollutant and each treatment. (continued)

**Substance**	**Preservation method**	**k** [h^{-1}]	$\mathbf{R^2}$	**p**	$\mathbf{t_{0.5}}$ [d^{-1}]	$\mathbf{k_{max}}$[a]	$\mathbf{k_{min}}$[b]	**non-valid**[c]
	short_lyo_AW	0.0673	0.991	* * *	10.3	FALSE	FALSE	FALSE
	short_dmso_SN	0.1665	0.999	* * *	4.16	FALSE	FALSE	FALSE
	short_dmso_AW	0.1872	0.993	* * *	3.7	FALSE	FALSE	FALSE
	short_gly_SN	0.1542	1	* * *	4.5	FALSE	FALSE	FALSE
	short_gly_AW	0.1649	0.997	* * *	4.2	FALSE	FALSE	FALSE
	long_lyo_SN	0.0295	0.975	* * *	23.5	FALSE	FALSE	FALSE
	long_lyo_AE	0.0292	0.996	* * *	23.74	FALSE	FALSE	FALSE
	long_dmso_SN	0.1724	0.991	* * *	4.02	FALSE	FALSE	FALSE
	long_dmso_AE	0.2076	0.994	* * *	3.34	FALSE	FALSE	FALSE
	long_gly_SN	0.1644	0.998	* * *	4.22	FALSE	FALSE	FALSE
	long_gly_AE	0.174	0.984	* * *	3.98	FALSE	FALSE	FALSE
Sitagliptin	f_fresh_SN	0.0614	0.999	* * *	11.29	FALSE	FALSE	FALSE
	f_fresh_AW	0.0972	0.999	* * *	7.13	FALSE	FALSE	FALSE
	short_lyo_SN	0.0361	0.997	* * *	19.19	FALSE	FALSE	FALSE
	short_lyo_AW	0.0421	0.999	* * *	16.45	FALSE	FALSE	FALSE
	short_dmso_SN	0.0513	0.999	* * *	13.51	FALSE	FALSE	FALSE
	short_dmso_AW	0.1041	0.999	* * *	6.66	FALSE	FALSE	FALSE
	short_gly_SN	0.061	1	* * *	11.36	FALSE	FALSE	FALSE
	short_gly_AW	0.0989	0.999	* * *	7.01	FALSE	FALSE	FALSE
	long_lyo_SN	0.0193	0.985	* * *	35.89	FALSE	FALSE	FALSE
	long_lyo_AE	0.0242	0.995	* * *	28.68	FALSE	FALSE	FALSE
	long_dmso_SN	0.0483	0.996	* * *	14.34	FALSE	FALSE	FALSE
	long_dmso_AE	0.0687	0.989	* * *	10.09	FALSE	FALSE	FALSE
	long_gly_SN	0.0602	0.994	* * *	11.52	FALSE	FALSE	FALSE
	long_gly_AE	0.0661	0.993	* * *	10.48	FALSE	FALSE	FALSE
Sulfadiazine	f_fresh_SN	0.036	0.97	* * *	19.23	FALSE	FALSE	FALSE
	f_fresh_AW	0.0928	0.969	* * *	7.47	FALSE	FALSE	FALSE

Continued on next page

Tbl. S14: Rate constants averaged over three replicates for each micropollutant and each treatment. (continued)

**Substance**	**Preservation method**	**k** $[h^{-1}]$	$\mathbf{R^2}$	**p**	$\mathbf{t_{0.5}}$ $[d^{-1}]$	$\mathbf{k_{max}}$ [a]	$\mathbf{k_{min}}$ [b]	**non-valid** [c]
	short_lyo_SN	0.1365	0.993	∗∗∗	5.08	FALSE	FALSE	FALSE
	short_lyo_AW	0.1565	0.983	∗∗∗	4.43	FALSE	FALSE	FALSE
	short_dmso_SN	0.0332	0.974	∗∗∗	20.9	FALSE	FALSE	FALSE
	short_dmso_AW	0.0891	0.98	∗∗∗	7.78	FALSE	FALSE	FALSE
	short_gly_SN	0.0315	0.95	∗∗∗	22.03	FALSE	FALSE	FALSE
	short_gly_AW	0.0986	0.987	∗∗∗	7.03	FALSE	FALSE	FALSE
	long_lyo_SN	0.0481	0.934	∗∗∗	14.41	FALSE	FALSE	FALSE
	long_lyo_AE	0.0655	0.978	∗∗∗	10.59	FALSE	FALSE	FALSE
	long_dmso_SN	0.0367	0.959	∗∗∗	18.87	FALSE	FALSE	FALSE
	long_dmso_AE	0.0399	0.921	∗∗∗	17.39	FALSE	FALSE	FALSE
	long_gly_SN	0.0331	0.956	∗∗∗	20.94	FALSE	FALSE	FALSE
	long_gly_AE	0.0443	0.931	∗∗∗	15.64	FALSE	FALSE	FALSE
Sulfamethazine	f_fresh_SN	0.0446	0.945	∗∗∗	15.53	FALSE	FALSE	FALSE
	f_fresh_AW	0.1373	0.993	∗∗∗	5.05	FALSE	FALSE	FALSE
	short_lyo_SN	0.2225	0.974	∗∗	3.12	FALSE	FALSE	FALSE
	short_lyo_AW	0.3427	0.996	∗∗∗	2.02	FALSE	FALSE	FALSE
	short_dmso_SN	0.0428	0.966	∗∗∗	16.19	FALSE	FALSE	FALSE
	short_dmso_AW	0.1354	0.983	∗∗∗	5.12	FALSE	FALSE	FALSE
	short_gly_SN	0.0424	0.943	∗∗∗	16.34	FALSE	FALSE	FALSE
	short_gly_AW	0.1418	0.991	∗∗∗	4.89	FALSE	FALSE	FALSE
	long_lyo_SN	0.0641	0.974	∗∗∗	10.81	FALSE	FALSE	FALSE
	long_lyo_AE	0.0687	0.928	∗∗∗	10.09	FALSE	FALSE	FALSE
	long_dmso_SN	0.0433	0.944	∗∗∗	16.02	FALSE	FALSE	FALSE
	long_dmso_AE	0.0483	0.889	∗∗∗	14.36	FALSE	FALSE	FALSE
	long_gly_SN	0.0401	0.925	∗∗∗	17.28	FALSE	FALSE	FALSE
	long_gly_AE	0.0414	0.808	∗∗	16.74	FALSE	FALSE	TRUE
Sulfamethoxazole	f_fresh_SN	0.0382	0.926	∗∗∗	18.16	FALSE	FALSE	FALSE

Continued on next page

Tbl. S14: Rate constants averaged over three replicates for each micropollutant and each treatment. (continued)

**Substance**	**Preservation method**	**k** [h^{-1}]	$\mathbf{R^2}$	**p**	$\mathbf{t_{0.5}}$ [d^{-1}]	$\mathbf{k_{max}}$[a]	$\mathbf{k_{min}}$[b]	**non-valid**[c]
	f_fresh_AW	0.0932	0.94	**	7.44	FALSE	FALSE	FALSE
	short_lyo_SN	0.1732	0.994	***	4	FALSE	FALSE	FALSE
	short_lyo_AW	0.182	0.983	***	3.81	FALSE	FALSE	FALSE
	short_dmso_SN	0.0384	0.947	***	18.06	FALSE	FALSE	FALSE
	short_dmso_AW	0.0876	0.958	***	7.92	FALSE	FALSE	FALSE
	short_gly_SN	0.0365	0.93	***	19.01	FALSE	FALSE	FALSE
	short_gly_AW	0.0949	0.969	***	7.31	FALSE	FALSE	FALSE
	long_lyo_SN	0.0493	0.884	***	14.07	FALSE	FALSE	FALSE
	long_lyo_AE	0.0625	0.945	***	11.08	FALSE	FALSE	FALSE
	long_dmso_SN	0.0321	0.924	***	21.61	FALSE	FALSE	FALSE
	long_dmso_AE	0.0345	0.867	***	20.1	FALSE	FALSE	FALSE
	long_gly_SN	0.0287	0.914	***	24.15	FALSE	FALSE	FALSE
	long_gly_AE	0.0374	0.852	**	18.54	FALSE	FALSE	FALSE
Sulfapyridine	f_fresh_SN	0.0983	0.999	***	7.05	FALSE	FALSE	FALSE
	f_fresh_AW	0.2852	0.992	***	2.43	FALSE	FALSE	FALSE
	short_lyo_SN	0.3072	0.983	***	2.26	FALSE	FALSE	FALSE
	short_lyo_AW	0.6185	0.998	**	1.12	FALSE	FALSE	FALSE
	short_dmso_SN	0.0614	0.983	***	11.3	FALSE	FALSE	FALSE
	short_dmso_AW	0.3029	0.999	***	2.29	FALSE	FALSE	FALSE
	short_gly_SN	0.0634	0.974	***	10.94	FALSE	FALSE	FALSE
	short_gly_AW	0.2736	0.999	***	2.53	FALSE	FALSE	FALSE
	long_lyo_SN	0.063	0.962	***	11	FALSE	FALSE	FALSE
	long_lyo_AE	0.0641	0.871	***	10.82	FALSE	FALSE	FALSE
	long_dmso_SN	0.0499	0.94	***	13.9	FALSE	FALSE	FALSE
	long_dmso_AE	0.0593	0.863	***	11.69	FALSE	FALSE	FALSE
	long_gly_SN	0.0482	0.92	***	14.37	FALSE	FALSE	FALSE
	long_gly_AE	0.0587	0.861	***	11.81	FALSE	FALSE	FALSE

Continued on next page

Tbl. S14: Rate constants averaged over three replicates for each micropollutant and each treatment. (continued)

**Substance**	**Preservation method**	**k** [h^{-1}]	$\mathbf{R^2}$	**p**	$\mathbf{t_{0.5}}$ [d^{-1}]	$\mathbf{k_{max}}$[a]	$\mathbf{k_{min}}$[b]	**non-valid**[c]
Sulfathiazole	f_fresh_SN	0.2249	1	***	3.08	FALSE	FALSE	FALSE
	f_fresh_AW	0.2503	0.999	***	2.77	FALSE	FALSE	FALSE
	short_lyo_SN	0.2183	0.996	***	3.18	FALSE	FALSE	FALSE
	short_lyo_AW	0.1525	0.966	**	4.55	FALSE	FALSE	FALSE
	short_dmso_SN	0.1962	0.995	***	3.53	FALSE	FALSE	FALSE
	short_dmso_AW	0.2044	0.995	***	3.39	FALSE	FALSE	FALSE
	short_gly_SN	0.1803	0.992	***	3.84	FALSE	FALSE	FALSE
	short_gly_AW	0.2295	0.995	***	3.02	FALSE	FALSE	FALSE
	long_lyo_SN	0.0701	0.95	***	9.89	FALSE	FALSE	FALSE
	long_lyo_AE	0.1276	0.992	***	5.43	FALSE	FALSE	FALSE
	long_dmso_SN	0.2225	0.995	***	3.12	FALSE	FALSE	FALSE
	long_dmso_AE	0.1693	0.994	***	4.09	FALSE	FALSE	FALSE
	long_gly_SN	0.1423	0.982	***	4.87	FALSE	FALSE	FALSE
	long_gly_AE	0.1517	0.995	***	4.57	FALSE	FALSE	FALSE
Tramadol	f_fresh_SN	0.0087	0.954	***	79.5	FALSE	FALSE	FALSE
	f_fresh_AW	0.0131	0.911	***	53.11	FALSE	FALSE	FALSE
	short_lyo_SN	0.0036	0.923	***	192.44	FALSE	FALSE	FALSE
	short_lyo_AW	0.0115	0.996	***	60.52	FALSE	FALSE	FALSE
	short_dmso_SN	0.0042	0.971	***	166.06	FALSE	FALSE	FALSE
	short_dmso_AW	0.0109	0.972	***	63.47	FALSE	FALSE	FALSE
	short_gly_SN	0.004	0.977	***	171.15	FALSE	FALSE	FALSE
	short_gly_AW	0.0086	0.983	***	80.21	FALSE	FALSE	FALSE
	long_lyo_SN	0.0027	0.783	**	259.03	FALSE	FALSE	TRUE
	long_lyo_AE	0.005	0.921	***	140.01	FALSE	FALSE	FALSE
	long_dmso_SN	0.0046	0.907	***	150.86	FALSE	FALSE	FALSE
	long_dmso_AE	0.008	0.959	***	86.76	FALSE	FALSE	FALSE
	long_gly_SN	0.0055	0.952	***	126.49	FALSE	FALSE	FALSE

Continued on next page

Tbl. S14: Rate constants averaged over three replicates for each micropollutant and each treatment. (continued)

**Substance**	**Preservation method**	**k** [h^{-1}]	$\mathbf{R^2}$	**p**	$\mathbf{t_{0.5}}$ [d^{-1}]	$\mathbf{k_{max}}$ [a]	$\mathbf{k_{min}}$ [b]	**non-valid** [c]
	long_gly_AE	0.0067	0.944	$***$	103.72	FALSE	FALSE	FALSE
Valsartan	f_fresh_SN	0.5463	0.999	$***$	1.27	FALSE	FALSE	FALSE
	f_fresh_AW	0.8236	0.994	$**$	0.84	FALSE	FALSE	FALSE
	short_lyo_SN	0.0475	0.96	$***$	14.59	FALSE	FALSE	FALSE
	short_lyo_AW	0.0206	0.726	$**$	33.61	FALSE	FALSE	TRUE
	short_dmso_SN	0.4413	1	$***$	1.57	FALSE	FALSE	FALSE
	short_dmso_AW	0.3167	0.967	$**$	2.19	FALSE	FALSE	FALSE
	short_gly_SN	0.417	0.999	$***$	1.66	FALSE	FALSE	FALSE
	short_gly_AW	0.2606	0.975	$**$	2.66	FALSE	FALSE	FALSE
	long_lyo_SN	$7e-04$	0.138	ns	979.74	FALSE	TRUE	TRUE
	long_lyo_AE	$4e-04$	0.055	ns	1747.51	FALSE	TRUE	TRUE
	long_dmso_SN	0.1886	0.858	$**$	3.68	FALSE	FALSE	FALSE
	long_dmso_AE	0.1944	0.86	$**$	3.57	FALSE	FALSE	FALSE
	long_gly_SN	0.1938	0.764	$*$	3.58	FALSE	FALSE	TRUE
	long_gly_AE	0.1897	0.836	$*$	3.65	FALSE	FALSE	TRUE
Venlafaxine	f_fresh_SN	0.0099	0.947	$***$	70.23	FALSE	FALSE	FALSE
	f_fresh_AW	0.0179	0.948	$***$	38.78	FALSE	FALSE	FALSE
	short_lyo_SN	0.0024	0.857	$***$	292.83	FALSE	FALSE	FALSE
	short_lyo_AW	0.0055	0.983	$***$	127	FALSE	FALSE	FALSE
	short_dmso_SN	0.0048	0.978	$***$	143.18	FALSE	FALSE	FALSE
	short_dmso_AW	0.0146	0.996	$***$	47.63	FALSE	FALSE	FALSE
	short_gly_SN	0.0027	0.915	$***$	258.28	FALSE	FALSE	FALSE
	short_gly_AW	0.0052	0.936	$***$	133.38	FALSE	FALSE	FALSE
	long_lyo_SN	$9e-04$	0.255	ns	777.48	FALSE	TRUE	TRUE
	long_lyo_AE	0.0012	0.318	ns	598.34	FALSE	TRUE	TRUE
	long_dmso_SN	0.0034	0.856	$***$	204.12	FALSE	FALSE	FALSE
	long_dmso_AE	0.0067	0.956	$***$	104.15	FALSE	FALSE	FALSE

Continued on next page

Tbl. S14: Rate constants averaged over three replicates for each micropollutant and each treatment. (continued)

**Substance**	**Preservation method**	**k** $[h^{-1}]$	$\mathbf{R^2}$	**p**	$\mathbf{t_{0.5}}$ $[d^{-1}]$	$\mathbf{k_{max}}$ [a]	$\mathbf{k_{min}}$ [b]	**non-valid** [c]
	long_gly_SN	0.0034	0.893	***	206.02	FALSE	FALSE	FALSE
	long_gly_AE	0.0042	0.889	***	163.75	FALSE	FALSE	FALSE

[a] k_{max}: if TRUE, less than 3 measurements above LOQ and/or $k > 1.151$ h^{-1}.
[b] k_{min}: if TRUE, less than 10% dissipation within 72 h ($k < 0.001$ h^{-1}).
[c] non-valid: if TRUE, k is considered non-valid, as $R^2 < 0.85$ and/or $p > 0.05$.

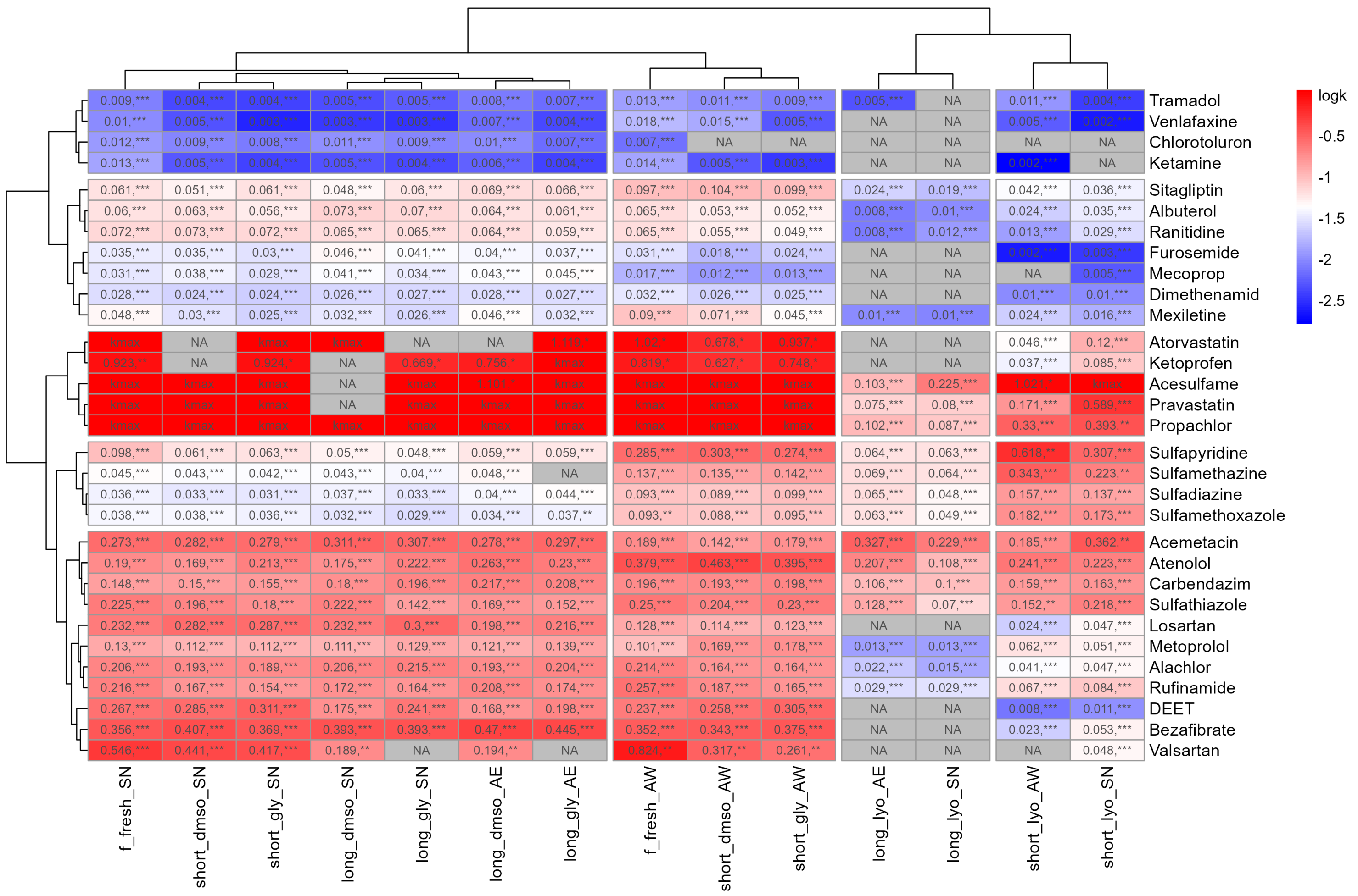

Fig. S9: Averaged rate constants [h^{-1}] with the statistically significant relationship between concentration and time: $p > 0.05$ (ns, non-significant), $p \leq 0.05$ (*), $p \leq 0.01$ (**), $p \leq 0.001$ (***). The color scale represents the decadic logarithm of the rate constants, the numbers the absolute rate constant in [h^{-1}]. Mean rate constants were not evaluated if validation criteria were not met for two out of three rate constants. NAs were omitted in the hierarchical clustering analysis. If at least two out of three rate constants were higher than $k_{\max}$, the mean rate constant was replaced by $k_{\max}$, and clustering was performed on nominal $k_{\max} = 1.1513\,h^{-1}$.

In most of the experiments and treatments four test substances (acesulfame, atorvastatin, pravastatin and propachlor) showed a faster depletion than our maximal quantifiable rate constant $k_{\mathrm{max}} = 1.1513\,\mathrm{h}^{-1}$ and were therefore excluded from further evaluation (see Fig. S9). For these four readily biotransformed MPs biotransformation kinetics appeared visually comparable between fresh and preserved AS suspensions except for LYO treatments in which depletion was slower.

Further, the rate constants k were compared by calculating the relative rate constant comparing the averaged absolute rate constant of each preservation treatment to the corresponding averaged fresh_fresh_SN absolute rate constant (Equ. 6). The significance of the relative rate constant change was further evaluated using a one-sample t-test after confirming normality with the Shapiro-Wilk test and homogeneity of variance with Bartlett's test.

$$100 * (\frac{k_{preserved}}{k_{fresh}} - 1) \tag{6}$$

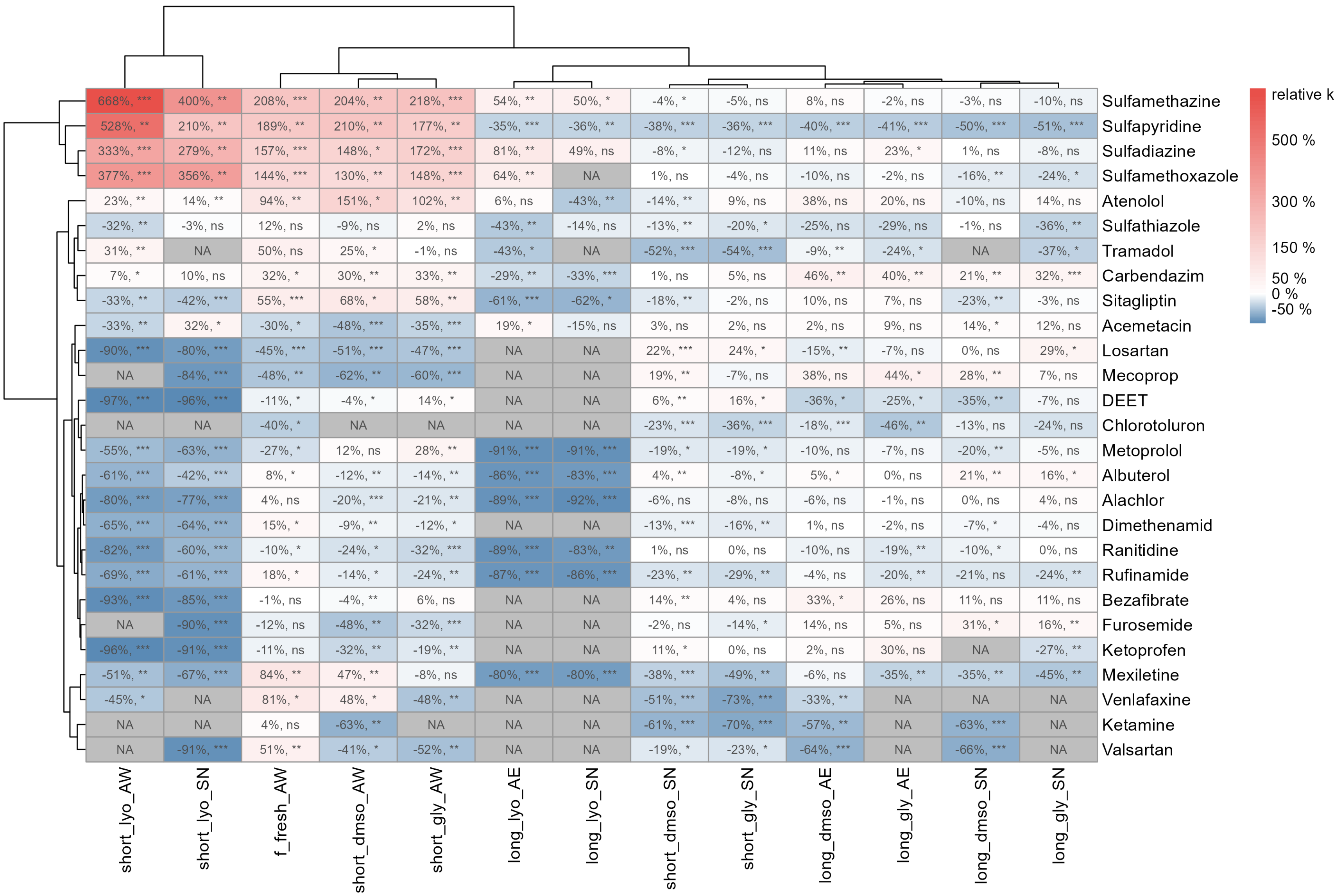

Fig. S10: Averaged relative rate constants towards the fresh_fresh_SN sample with statistical significance: $p > 0.05$ (ns, non-significant), $p \leq 0.05$ (*), $p \leq 0.01$ (**), $p \leq 0.001$ (***). The color scale represents the relative deviation to the corresponding rate constant determined in the fresh_SN experiment. The relative rate constants were not evaluated if two out of three rate constants did not meet the validation criteria (NA) and were omitted in the hierarchical clustering.

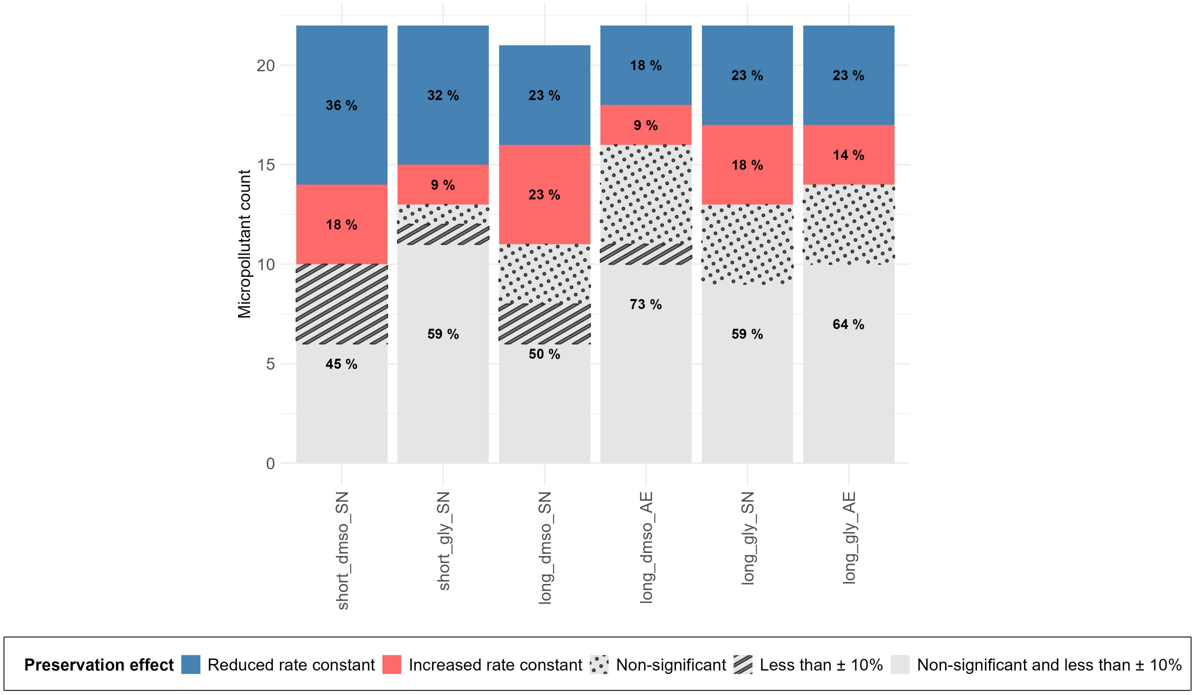

Fig. S11: Count of relative rate constants without LYO method and without treatments with AW as resuspension media, excluding substances prone to protozoic bioaccumulation. In total 22 MPs.

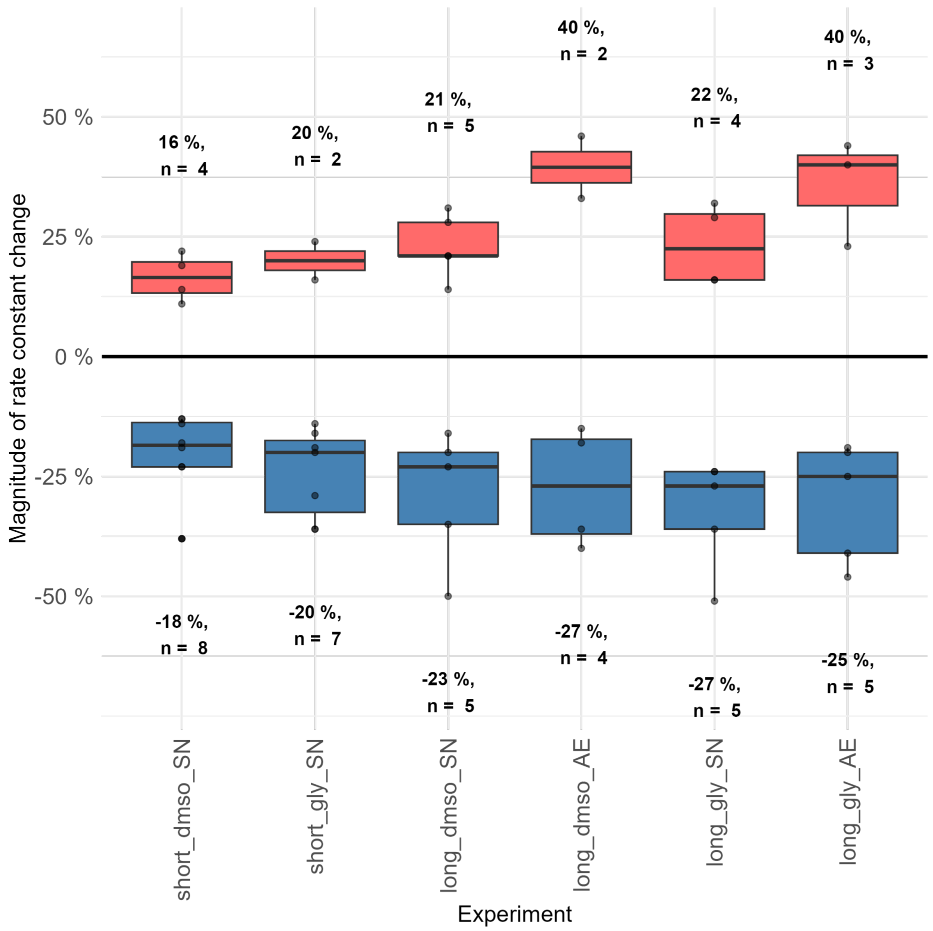

Fig. S12: Relative magnitude shifts of rate constants without LYO method and without treatments with AW as resuspension media, excluding substances prone to protozoic bioacchumulation. Red indicating increased rate constants (> 10 %), blue indicating reduced rate constants (< −10 %). Non-significantly and/or > ±10 % are not depicted.

References

(1) Yu, C.; Reddy, A. P.; Simmons, C. W.; Simmons, B. A.; Singer, S. W.; VanderGheynst, J. S. Preservation of microbial communities enriched on lignocellulose under thermophilic and high-solid conditions. *Biotechnology for Biofuels* **2015**, *8*, 206.

(2) Kerckhof, F. M.; Courtens, E. N.; Geirnaert, A.; Hoefman, S.; Ho, A.; Vilchez-Vargas, R.; Pieper, D. H.; Jauregui, R.; Vlaeminck, S. E.; Van de Wiele, T.; Vandamme, P.; Heylen, K.; Boon, N. Optimized cryopreservation of mixed microbial communities for conserved functionality and diversity. *PLoS One* **2014**, *9*, e99517.

(3) Hoefman, S.; Van Hoorde, K.; Boon, N.; Vandamme, P.; De Vos, P.; Heylen, K. Survival or revival: long-term preservation induces a reversible viable but non-culturable state in methane-oxidizing bacteria. *PLoS One* **2012**, *7*, e34196.

(4) Hoefman, S.; Pommerening-Roser, A.; Samyn, E.; De Vos, P.; Heylen, K. Efficient cryopreservation protocol enables accessibility of a broad range of ammonia-oxidizing bacteria for the scientific community. *Research in Microbiology* **2013**, *164*, 288–92.

(5) Bellali, S.; Bou Khalil, J.; Fontanini, A.; Raoult, D.; Lagier, J. C. A new protectant medium preserving bacterial viability after freeze drying. *Microbioligy Research* **2020**, *236*, 126454.

(6) Yarberry, A.; Lansing, S.; Luckarift, H.; Diltz, R.; Mulbry, W.; Yarwood, S. Effect of anaerobic digester inoculum preservation via lyophilization on methane recovery. *Waste Management* **2019**, *87*, 62–70.

(7) Rothrock, J., M. J.; Vanotti, M. B.; Szogi, A. A.; Gonzalez, M. C.; Fujii, T. Long-term preservation of anammox bacteria. *Applied Microbiology and Biotechnology* **2011**, *92*, 147–57.

(8) Heylen, K.; Ettwig, K.; Hu, Z.; Jetten, M.; Kartal, B. Rapid and simple cryopreservation of anaerobic ammonium-oxidizing bacteria. *Applied and Environmental Microbiology* **2012**, *78*, 3010–3.

(9) Laurin, V.; Labbe, N.; Juteau, P.; Parent, S.; Villemur, R. Long-term storage conditions for carriers with denitrifying biomass of the fluidized, methanol-fed denitrification reactor of the Montreal Biodome, and the impact on denitrifying activity and bacterial population. *Water Research* **2006**, *40*, 1836–40.

(10) Hubalek, Z. Protectants used in the cryopreservation of microorganisms. *Cryobiology* **2003**, *46*, 205–29.

(11) Common Access to Biological Resources and Information (CABRI) Protective suspension media for freezing or (freeze)-drying. `http://www.cabri.org/guidelines/micro-organisms/M300Ap3.html`, (accessed: 2025-02-06).

(12) Prakash, O.; Nimonkar, Y.; Shouche, Y. S. Practice and prospects of microbial preservation. *FEMS Microbiology Letters* **2013**, *339*, 1–9.

(13) Whaley, D.; Damyar, K.; Witek, R. P.; Mendoza, A.; Alexander, M.; Lakey, J. R. Cryopreservation: An Overview of Principles and Cell-Specific Considerations. *Cell Transplantation* **2021**, *30*, 963689721999617.

(14) Gurtovenko, A. A.; Anwar, J. Modulating the Structure and Properties of Cell Membranes: The Molecular Mechanism of Action of Dimethyl Sulfoxide. *The Journal of Physical Chemistry B* **2007**, *111.35*, 10453–10460.

(15) Best, B. P. Cryoprotectant Toxicity: Facts, Issues, and Questions. *Rejuvenation Research* **2015**, *18*, 422–36.

(16) Seller-Brison, C.; Brison, A.; Yu, Y.; Robinson, S. L.; Fenner, K. Adaptation towards catabolic biodegradation of trace organic contaminants in activated sludge. *Water Res* **2024**, *266*, 122431.

(17) Hung, Y.-M.; Lu, T.-P.; Tsai, M.-H.; Lai, L.-C.; Chuang, E. Y. EasyMAP: A user-friendly online platform for analyzing 16S ribosomal DNA sequencing data. *New Biotechnology* **2021**, *63*, 37–44.

(18) Caporaso, J. G. et al. QIIME allows analysis of high-throughput community sequencing data. *Nature methods* **2010**, *7*, 335.

(19) Callahan, B. J.; McMurdie, P. J.; Rosen, M. J.; Han, A. W.; Johnson, A. J. A.; Holmes, S. P. DADA2: high-resolution sample inference from Illumina amplicon data. *Nature methods* **2016**, *13*, 581.

(20) DeSantis, T. Z.; Hugenholtz, P.; Larsen, N.; Rojas, M.; Brodie, E. L.; Keller, K.; Huber, T.; Dalevi, D.; Hu, P.; Andersen, G. L. Greengenes, a chimera-checked 16S rRNA gene database and workbench compatible with ARB. *Applied and Environmental Microbiology* **2006**, *72*, 5069–5072.

(21) Bokulich, N. A.; Kaehler, B. D.; Rideout, J. R.; Dillon, M.; Bolyen, E.; Knight, R.; Huttley, G. A.; Caporaso, J. G. Optimizing taxonomic classification of marker-gene amplicon sequences with QIIME 2's q2-feature-classifier plugin. *Microbiome* **2018**, *In Press*.

(22) Pedregosa, F. et al. Scikit-learn: Machine learning in Python. *Journal of Machine Learning Research* **2011**, *12*, 2825–2830.

(23) Calle, M. L. Statistical analysis of metagenomics data. *Genomics & informatics* **2019**, *17*.

(24) Xia, Y.; Sun, J. *Bioinformatic and Statistical Analysis of Microbiome Data: From Raw Sequences to Advanced Modeling with QIIME 2 and R*; Springer, 2023; pp 289–333.

(25) Kim, T. K. T test as a parametric statistic. *Korean Journal of Anesthesiology* **2015**, *68*, 540.

(26) Namkung, J. Machine learning methods for microbiome studies. *Journal of Microbiology* **2020**, *58*, 206–216.

(27) Achermann, S.; Falas, P.; Joss, A.; Mansfeldt, C. B.; Men, Y.; Vogler, B.; Fenner, K. Trends in Micropollutant Biotransformation along a Solids Retention Time Gradient. *Environmental Science & Technology* **2018**, *52*, 11601–11611.

(28) Desiante, W. L.; Carles, L.; Wullschleger, S.; Joss, A.; Stamm, C.; Fenner, K. Wastewater microorganisms impact the micropollutant biotransformation potential of natural stream biofilms. *Water Research* **2022**, *217*, 118413.

Glossary

AE artifical effluent. S17

AS activated sludge. S5

AW artifical wastewater. S10

CPA cryoprotective agent. S8

cryo cryopreservation. S5

dmso dimethyl sulfoxide. S47

gly glycerol. S9

LPA lyoprotective agent. S8

lyo lyophilization. S8

MP micropollutant. S48

OTUs operational taxonomic units. S36

PCoA principal coordinate analysis. S31

SC sorption control. S49

SN activated sludge supernatant. S19

WWTP wastewater treatment plant. S15
